# Differentiation status determines the effects of IFNγ on the expression of PD-L1 and immunomodulatory genes in melanoma

**DOI:** 10.1186/s12964-024-01963-6

**Published:** 2024-12-31

**Authors:** Teitur Sævarsson, Adrián López García de Lomana, Ólafur Sánchez, Veerle van Esch, Gunnar Bjarni Ragnarsson, Siggeir Fannar Brynjólfsson, Eiríkur Steingrímsson, Berglind Ósk Einarsdóttir

**Affiliations:** 1 Department of Biomedical Science, Faculty of Medicine, BioMedical Center, University of Iceland, Reykjavík, Iceland; 2 Department of Biochemistry and Molecular Biology, Faculty of Medicine, BioMedical Center, University of Iceland, Reykjavík, Iceland; 3 Landspítali – The National University Hospital of Iceland, Reykjavík, Iceland; 4 Department of Immunology, Landspítali – The National University Hospital of Iceland, Reykjavík, Iceland; 5 Faculty of Medicine, University of Iceland, Reykjavík, Iceland

**Keywords:** Melanoma, Dedifferentiation, Interferon-γ, JAK/STAT pathway, PD-L1

## Abstract

**Background:**

Melanoma cells frequently dedifferentiate in response to inflammation which can increase responses to certain cytokines. Interferon-γ (IFNγ) is an integral part of the anti-tumor immune response and can directly induce both differentiational changes and expression of immunosuppressive proteins in melanoma cells. How the differentiation status of melanoma cells affects IFNγ responses remains unclear.

**Methods:**

Dedifferentiation of melanoma cells was induced via either siRNA or shRNA mediated MITF knockdown and the cells were subsequently treated with IFNγ. Effects of MITF knockdown and IFNγ treatment on gene expression were evaluated via qPCR and RNA sequencing. A Luminex assay was used to analyze the effects of dedifferentiation and IFNγ treatment on cytokine secretion. Effects on PD-L1 protein expression were analyzed via flow cytometry and western blotting. Inhibition of the JAK kinases, NF-κB and STAT3 with small molecule inhibitors, and siRNA mediated knockdown of STAT1 and IRF1 was applied to investigate the molecular mechanism behind IFNγ induced PD-L1 expression in dedifferentiated melanoma cells. The effects of inhibitor treatments and siRNA mediated knockdowns were evaluated via qPCR and western blotting. Bioinformatic analysis of publicly available RNA sequencing data, consisting of 45 patient derived melanoma cell lines, with or without IFNγ treatment, was conducted to assess the generalizability of the in vitro results.

**Results:**

Dedifferentiation renders 624Mel melanoma cells hypersensitive to IFNγ stimulation in a context-dependent manner, resulting in non-additive upregulation of IFNγ-induced genes, increased PD-L1 protein expression and amplified secretion of CCL2, CXCL10 and IL-10. Furthermore, the intensified PD-L1 protein expression occurs through the JAK-STAT1-IRF1 axis. Lastly, dedifferentiated patient derived melanoma cell lines showed enhanced inflammatory signaling in response to IFNγ compared to differentiated cells, and tended to have higher PD-L1 expression, associated with increased IRF1 expression and activity.

**Conclusions:**

Together, these findings indicate the existence of a molecular context linking dedifferentiation and IFNγ signaling in melanoma which may lead to immune evasion. Additionally, the variability in PD-L1 expression among MITF^low^ and MITF^high^ cells suggests that high IFNγ-induced PD-L1 expression associates with enhanced inflammatory gene expression. These results imply that modulating melanoma differentiation may help shape IFNγ responsiveness.

**Graphical Abstract:**

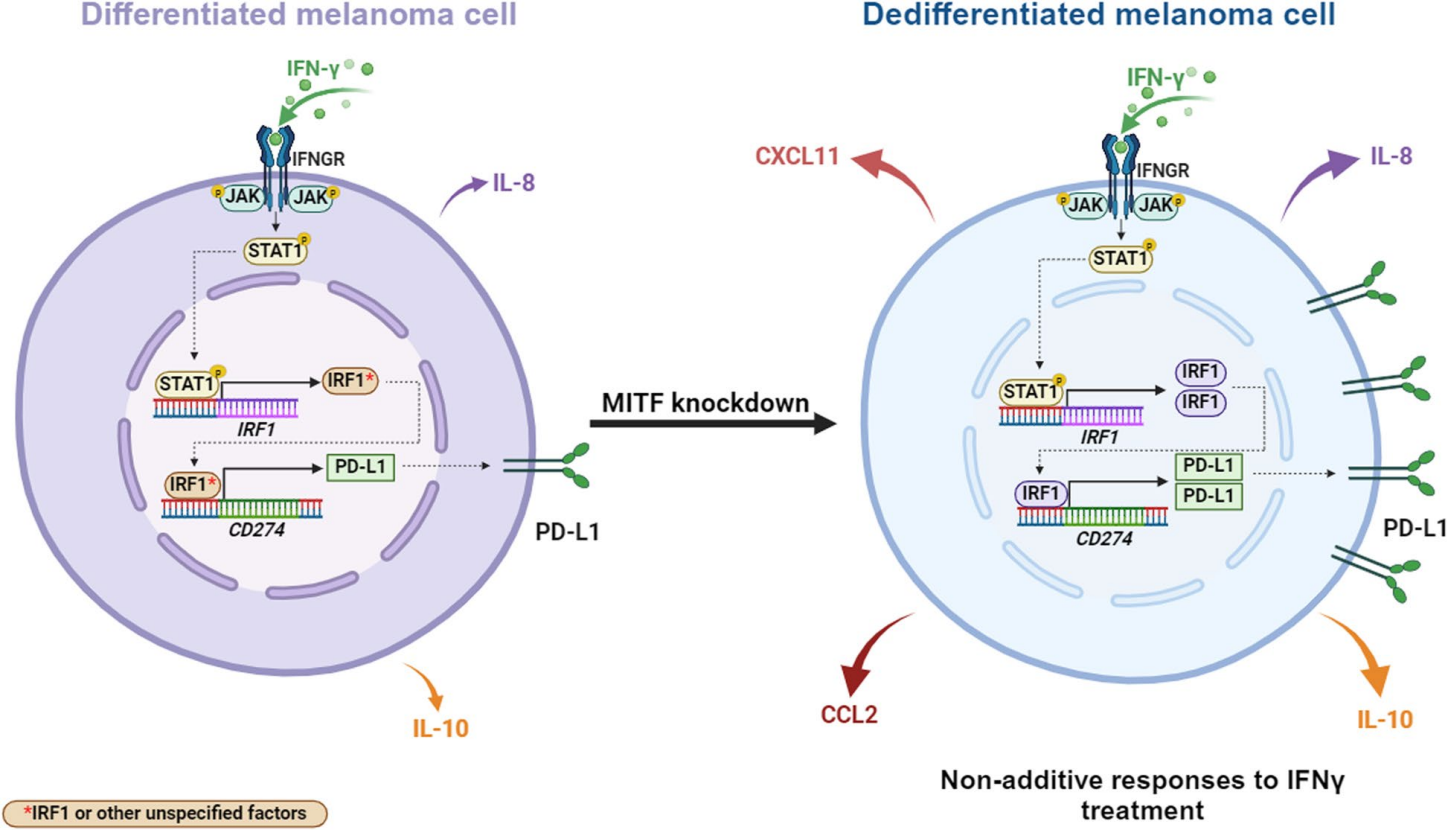

**Supplementary Information:**

The online version contains supplementary material available at 10.1186/s12964-024-01963-6.

## Background

Phenotypic plasticity allows melanoma cells to reverse their differentiation status in response to changes in the tumor microenvironment (TME) [1–6]. This process has been implicated in resistance to both targeted therapies and immunotherapies [7–10]. Melanoma dedifferentiation is a gradual process that entails a loss of melanocytic antigens and a gain of neural crest markers and can occur through reduced expression of the microphthalmia associated transcription factor (MITF), a key regulator of the melanocytic lineage [8, 11–16]. Importantly, MITF is recognized as a major determinant of phenotypic identity in melanoma cells where it promotes differentiation by both positive regulation of melanocytic genes and by repressing expression of genes that induce dedifferentiation [11, 12]. Studies on melanoma cells expressing high versus low MITF have shown that dedifferentiation has complex immunological implications [17]. In addition to decreased melanoma antigen expression and presentation, melanoma cells with low MITF expression have been reported to develop an inflammatory secretome and display altered expression of the immune checkpoint protein programmed death ligand 1 (PD-L1) [18–21]. However, our current understanding of how dedifferentiation affects PD-L1 expression is inconsistent as studies have shown both a positive and a negative correlation between MITF and PD-L1 expression. For example, Yokoyama et al. [21] demonstrated that knocking down SOX10, a positive regulator of MITF [22], leads to an increase in PD-L1 expression [21]. Supporting that, another study revealed that the aloperine derivative SA-49 triggers MITF-dependent lysosomal degradation of PD-L1 [19]. In contrast, MITF has been identified as a positive regulator of PD-L1 in melanocytes and melanoma cells and myeloid-derived suppressor cells [23, 24]. Consistent with that, another study showed that PD-L1 levels positively correlate with MITF expression in melanoma patient biopsies [20]. Lastly, a recent study reported positive regulation of PD-L1 by SOX10, resulting in impaired T cell recognition of A375 melanoma cells [25]. Similarly, it has been demonstrated that both innate and acquired resistance to targeted therapies, a process that is in part characterized by dedifferentiation, can have contrasting effects on PD-L1 expression in melanoma cell lines [15, 26]. From these studies, it is clear that shifts in PD-L1 expression during dedifferentiation are context-dependent and not universally affected across all melanomas.

The ability of melanoma cells to reversibly dedifferentiate has been studied in various different environmental contexts [1–4, 9, 18]. Among them are inflammation and stimulation with inflammatory cytokines. For example, it has been shown that melanoma cells can undergo dedifferentiation when stimulated with TNFα. This is shown both in in vitro experiments and in vivo where TNFα is secreted from T cells during adoptive T cell therapy [2, 4, 9]. This effect was mediated through increased AP-1 and NF-κB activity, which was attenuated by MITF overexpression [2]. Similarly, interferon-γ (IFNγ) stimulation has been shown to induce dedifferentiation in melanoma cells [4, 21]. IFNγ is a pluripotent pro-inflammatory cytokine known to have both pro- and anti-tumour effects [27–32]. It has a dual role in melanoma progression where conserved IFNγ signaling is essential for response to immune checkpoint inhibitor (ICI) therapies [4, 33], whilst it can also confer increased metastatic potential and promote multigenic ICI resistance [34, 35]. Importantly, IFNγ is known to induce PD-L1 expression in melanoma cells through the JAK1/2-STAT1/3-IRF1 axis [27, 36], and there are indications that some melanoma cell lines display increased IFNγ-induced PD-L1 expression when dedifferentiated [21, 37]. However, the molecular mechanism governing the effects of IFNγ on the expression of PD-L1 and other immunological genes in dedifferentiated melanoma cells remains unknown.

In this study, we investigated how the differentiation status of melanoma cells affects their response to IFNγ. Our results suggest that dedifferentiation upon MITF knockdown modifies IFNγ sensitivity, leading to a synergistic increase in the expression of genes taking part in immune regulation. Furthermore, we show that IFNγ stimulated dedifferentiated melanoma cells display increased secretion of CCL2, IL-10, CXCL10 and PD-L1 and increased PD-L1 expression on their cell membranes. Additionally, we demonstrate that the increased PD-L1 expression is mediated through the JAK-STAT1-IRF1 axis. Our findings indicate that dedifferentiation synergistically conditions canonical IFNγ signaling, rather than activating an alternative pathway leading to PD-L1 expression. Importantly, this was only observed in the 624Mel cell line, suggesting that this is context-dependent and not universal to all melanomas. However, analysis of 45 patient derived melanoma cell lines indicates a trend of high PD-L1 expression in dedifferentiated melanoma cells, associated with broadly enhanced inflammatory signaling upon IFNγ stimulation. Together, our results indicate that the differentiational status of melanoma cells may be a proxy for anti-tumor immune responses.

## Materials and methods

### Cell culture

The human melanoma cell lines 624Mel, SK-MEL-28, A375P and Malme-3M were maintained in RPMI 1640 medium supplemented with L-glutamine, 25 mM HEPES buffer and 10% fetal bovine serum. The mouse melanoma cell line B16 was maintained in DMEM with 10% FBS, whilst the mouse melanoma cell line YUMM.17 was maintained in DMEM/F-12 containing 10% FBS and 1% non-essential amino acids (MEM-NEAA). HEK-293T cells were maintained in DMEM/F-12 with 10% FBS. All cell culture medium was supplemented with 1% penicillin/streptomycin (P/S), unless stated otherwise. All cell culture medium and the corresponding supplements were acquired from Gibco. Cells were grown under stable temperature and atmospheric conditions at 37°C and 5% CO_2_, respectively, in a humidified incubator. All cell lines consistently tested negative for mycoplasma when tested on a three-month basis. The 624Mel line was authenticated based on STR profiling, through cell line authentication services provided by Eurofins Genomics.

For siRNA transfections, 100,000 cells were seeded per well in a 6-well plate in antibiotic free medium and reverse transfected using 250 μL of a transfection mixture consisting of 30 pmol siRNA and 9 μL RNAiMAX Lipofectamine (Thermo Scientific) and Opti-MEM (Gibco). The transfection mixtures were prepared by separately combining 150 μL Opti-MEM with either the RNAiMAX Lipofectamine or the corresponding siRNA. The two solutions were then combined, mixed by gently pipetting ~ 5 times and incubated for 5 min at room temperature prior to use. The siRNA constructs that were used include the Silencer Select Negative Control No. 1 siRNA, s8792 targeting human MITF, s277 targeting human STAT1, and s7501 targeting human IRF1. All siRNA constructs were from Thermo Scientific. Transfections lasted for 48–72 h. Recombinant human and mouse IFNγ/Ifnγ were acquired from R&D Systems and Abcam (ab123747), respectively, and both were used at a concentration of 5 ng/mL. JAK inhibitor I was acquired from Stemcell Technologies and used at concentrations of 300 nM and 900 nM. STAT3 inhibitor (Stattic, Selleckchem, S7024) was used at a 1 μM concentration, and NF-κB inhibitor was acquired from Stemcell Technologies (QNZ, #73,352) and was used at a 100 nM concentration.

### Generation of lentiviral particles

One Hundred and Fifty Thousand HEK-293T cells were seeded per well in a 6-well plate using antibiotic free medium. The cells were transfected 24 h post-seeding with 1.2 μg packaging plasmid (Origene) and 1 μg of either the pLV[shRNA]-EGFP:T2A:Puro-U6 > mMitf[shRNA#3] or pLV[shRNA]-EGFP:T2A:PuroU6 > Scramble_shRNA lentiviral vectors (both acquired in glycerol stocks from Vectorbuilder). The DNA constructs were diluted in 250 μL Opti-MEM, with 6.6 μL TurboFectin (Origene) added to the mixture post dilution, followed by gentle mixing and 15-min incubation at room temperature. The transfection mixtures were then added dropwise to each corresponding well and mixed via gentle rocking of the culture vessels before placing the cells back in the cell culture incubator. The cell culture medium was changed after 12–18 h of incubation. Viral supernatants were collected in two batches at 24 and 48 h after the initial medium exchange. The viral supernatants were filtered through a 0.22 μM syringe filter.

### Production of stable knockdown Mitf^low^ mouse melanoma cell lines

Fifty thousand YUMM1.7 cells were seeded per well in a 6 well plate 24 h prior to transduction. For the lentiviral transductions, the cell culture medium was exchanged for 2 mL fresh medium and 1 mL of viral supernatant. Viral supernatants contained either shRNA targeting mouse Mitf (shMitf) or a scrambled sequence as control (shCTRL). The cell culture medium was changed 24 h post transduction. At 48 h post transduction, the transduced cells were subjected to antibiotic selection using 1 μg/mL puromycin. Non-transduced YUMM1.7 cells were used as selection control. The antibiotic selection lasted until all the untreated cells in the selection control had died.

### RNA isolation and quantitative RT-PCR

Total RNA was extracted using a Quick-RNA Miniprep Kit acquired from Zymo Research. The standard protocol of the kit was followed with an additional washing step using 400 μL RNA wash buffer prior to the elution step. The columns were incubated at room temperature for 2 min after adding the DNase/RNase-Free Water to the column matrices during the elution step, prior to the final centrifugation step. Quality and concentrations of RNA samples were estimated using a Nanodrop One spectrometer. The cDNA synthesis was performed with a High-Capacity cDNA Reverse Transcription Kit with RNase Inhibitor (Thermo Scientific), using 500 ng RNA and 25 U MultiScribe Reverse Transcriptase in a 10 μL reaction. Each reaction was incubated in a thermal cycler for 10 min at 25°C, followed by 2 h at 37°C, followed by a 5-min inactivation step at 85°C. All quantitative RT-PCR reactions were run in technical triplicates (per biological replicate) in 10 μL reaction volumes consisting of 5 μL SensiFAST SYBR lo-ROX Mix (Meridian Bioscience), 4 μL cDNA and 1 μL of mixed forward and reverse primers (each primer at 2.5 μM concentration). The reactions were performed using a CFX384 Real-Time System & C1000 Touch Thermal Cycler (Bio-Rad). Results were quantified and normalized using the 2-ΔΔCT method, with *GAPDH* as housekeeping gene. All primer sequences used in this study are listed in Supplementary Table S1.

### RNA sequencing

For cDNA library preparation, each RNA sample was diluted to 20 ng/μL in 50 μL final volumes. The cDNA libraries were then made using the TruSeq RNA v2 Sample Prep Kit (Illumina) and SuperScript IV Reverse Transcriptase (Invitrogen). The cDNA libraries were quantified using LabChip GX and diluted to 3 nM prior to sequencing. The sequencing was performed on a NovaSeq 6000 sequencer, and the results were exported as FASTQ files using bclfastq2 v2.20. RNA-seq data are deposited in the Gene Expression Omnibus (GEO) repository with accession number GSE283655.

### Gene expression quantification

First, we cleaned the original FASTQ files using Trimmomatic version 0.39 using default parameters [38]. Next, we quantified gene expression from generated FASTQ files using Kallisto version 0.46.1 [39] and the Ensembl *Homo sapiens* GRCh38 reference transcriptome.

### Differential gene expression analysis

We used DESeq2 version 1.26.0 [40] to determine statistically significant differentially expressed genes (DEGs) across experimental conditions (Benjamini–Hochberg correction α = 0.1; adjusted *P *< 0.05). Next, we filtered out those DEGs that, when compared across conditions were expressed at very low levels (max expression < 2 TPM) or showed relatively small fold-change (FC) differences (abs log_2_ FC < 1). This filter resulted in a set of 1,129 response genes that we used as input for downstream non-additive response analysis.

### Non-additive response analysis

We applied the quantitative framework defined by Piggot et al. (2015) [41] to identify non-additive effects on gene expression for siMITF and IFNγ treatments. We identified a set of 148 non-additive response genes in concurrent siMITF and IFNγ treatments after applying the following rules based on significance and effect size: significant interaction term (likelihood-ratio test from DESeq2; corrected *P* < 0.05) and substantial non-additive effect (abs log_2_ FC > 1). All code to reproduce gene expression quantitative analysis is available in GitHub repository https://github.com/adelomana/nautholsvik.

### Gene set enrichment analysis (GSEA)

GSEA was performed using the GSEA v4.3.2 software (https://www.gsea-msigdb.org/gsea/downloads.jsp)(42) and four gene sets generated by Tsoi et al. (2018) [8] to categorize melanoma cells by differentiation levels. The input data consisted of z-values from log_2_(TPM + 1) transformed RNA-seq data described earlier. Genes with TPM < 3 for every sample were excluded from the analysis. The default setting of 1,000 permutations was used with gene set permutation mode. Two separate analyses were performed, comparing the untreated siCTRL and siMITF groups on the one hand, and the IFNγ treated siCTRL and siMITF groups on the other.

### Gene ontology analysis

Four lists of genes responding non-additively to siMITF and/or IFNγ, or control treatment, were separately imported to the Gene Set Enrichment Analysis v. 4.3.2 from the Broad Institute (https://www.gsea-msigdb.org/gsea/msigdb/human/annotate.jsp), to analyze “Gene Ontology: biological process” and “transcription factor targets” of the genes. *P*-values were calculated based on permutation tests, and q-values were calculated for false discovery rate (FDR) control [42, 43].

### Luminex discovery assay

Conditioned cell culture supernatants from 624Mel cells treated with either siCTRL or siMITF, with or w/o IFNγ stimulation, were loaded onto a Human Premixed Multi-Analyte Luminex Discovery Assay (R&D Systems, catalog number: LXSAHM-18), measuring the cytokines CCL2/JE/MCP-1, CCL27/CTACK, CXCL2/GRO beta/MIP-2/CINC-3, CXCL10-IP-10/CRG-2, IFN-β, IL-6, IL-8/CXCL8, IL-21, PD-L1/B7-H1, CCL5/RANTES, CXCL1/GRO alpha/KC/CINC-1, CXCL9/MIG, CXCL11/I-TAC, IL-2, IL-7, IL-10, IL-27, and VEGF. Each sample was diluted twofold with Calibrator Diluent RD6-52 according to the assay instructions. The standard protocol of the kit was followed during the assay procedure. Standards and samples were loaded in technical duplicates. Plate washes were performed using the Bio-Plex Pro Wash Station (Bio-Rad) and the assay was run using the Bio-Plex 200 System (Bio-Rad). The results were analyzed using GraphPad Prism 10 and Microsoft Excel.

### Fluorescence activated cell sorting

For FACS analysis, cells were harvested using 0.5 mL of 100 μM EDTA and stained with 0.5 μg per sample of the rabbit monoclonal Recombinant Anti-PD-L1 antibody (ab205921 [28–8], Abcam). PD-L1 protein expression was analyzed using an Attune NxT acoustic focusing cytometer (Life Technologies). FACS results were analyzed using the FlowJo software (BD Biosciences). Quantitative results for PD-L1 expression were extracted from FlowJo as geometric means and normalized using Microsoft Excel.

### Western blotting

Total cell lysates were prepared by lysing the cells in 1 × Laemmli buffer and incubating the lysates for 5 min at 95°C. The lysates were separated via 8% (for PD-L1, IRF1, STAT1, pSTAT1) or 12% (for MITF) SDS-PAGE, followed by a transfer to a PVDF membrane (Thermo Scientific) by wet blotting overnight at 20–25 V and 4°C, using PowerPac 300 (BioRad). The membranes were blocked in 5% BSA (Sigma-Aldrich) in TBS with 0.1% Tween20 (Sigma-Aldrich) for 1 h at room temperature on a shaker. Subsequently, the membranes were incubated with primary antibodies overnight at 4°C in 3% BSA in TBS with 0.1% Tween20, on a rocking platform. Target proteins were detected using Anti-mouse IgG (HHL) (Dylight 800 4 × PEG Conjugate) and Anti-rabbit IgG (HHL) (Dylight 680 Conjugate) secondary antibodies (both from Cell Signaling) and the Odyssey CLx Imaging System (LI-COR Biosciences). The antibodies used in this study include: Anti-actin (clone C4, Millipore, MAB1501, 1:5000), β-actin (D6A8, Cell Signaling, #8457, 1:2500), IRF-1 (D5E4, Cell Signaling, #8478, 1:1000), PD-L1 (E1L3N, Cell Signaling, #13,684, 1:1000), Stat1 (D1K9Y, Cell Signaling, #14,994, 1:1000), p-Stat1 (Y701) (D4A7, Cell Signaling, #7649, 1:1000) and Anti-Microphthalmia (clone c5, Millipore, MAB3747, 1:500). A PageRuler™ Prestained Protein Ladder, 10 to 180 kDa (Thermo Scientific, #26,616) was used to estimate the molecular weight of the observed bands. The results were quantified using ImageJ (NIH) and normalized using Microsoft Excel.

### Analysis of published melanoma RNA sequencing data

Gene expression data from the GSE154996 data set (*n* = 58 patient-derived melanoma cell lines, with or without 6-h stimulation with 5 ng/mL IFNγ) was downloaded from GEO as a matrix text file containing fragments per kilobase of exon per million fragments mapped (FPKM) expression values resulting from the initial data processing by the authors [33]. All genetically engineered cells and cells treated with other than IFNγ were excluded from the data set, leaving 45 wild type melanoma cell lines that were used for the analysis. FPKM expression values were converted to transcripts per million (TPM) values using the following formula:

$${TPM}_{i}=\left(\frac{{FPKM}_{i}}{{\sum }_{j}{FPKM}_{j}}\right)*{10}^{6}$$

Each gene containing TPM < 3 in every cell line was excluded from the analysis. Expression values were transformed by log_2_(TPM + 1), and z-values were subsequently generated. The IFNγ response of each cell line was determined by calculating the mean log_2_(TPM + 1) expression of all genes in the IFNγ response hallmark gene set. Association between PD-L1 mRNA expression and either IFNγ response or MITF mRNA expression was quantified by two-tailed Spearman correlation and simple linear regression analysis. Correlation matrices with unsupervised clustering were generated using the R corrr package. Principal component analyses were performed using the prcomp function in R, with the z-values as the input expression data. GSEA was performed using the same default settings described previously and the curated Hallmark gene set and C3 transcription factor target gene (TFT) set collections (BROAD molecular signature database, MSigDB, https://www.gsea-msigdb.org/gsea/msigdb/) [44], as well as the Tsoi differentiation gene sets [8]. In addition to R and GSEA v4.3.2 software, the data was processed and analyzed using Microsoft Excel and GraphPad Prism 10.

### Statistical tests

All experiments were performed in at least three independent biological replicates. Statistical analysis was performed using GraphPad Prism 10. We used unpaired Student’s t-tests for comparing two groups with normally distributed data, Mann–Whitney U tests and paired two-tailed Wilcoxon tests for non-normally distributed data. For multiple groups comparisons of normally distributed data, we used one-way ANOVA and Tukey’s test for post-hoc testing. For multiple groups comparisons of non-normally distributed data, we used Kruskal–Wallis tests and Dunn’s tests for post-hoc testing The respective statistical tests for each experiment are specified in the figure legends throughout the manuscript.

## Results

### MITF knockdown together with IFNγ stimulation results in non-additive activation of immunomodulatory genes in 624Mel melanoma cells

**Fig. 1:**
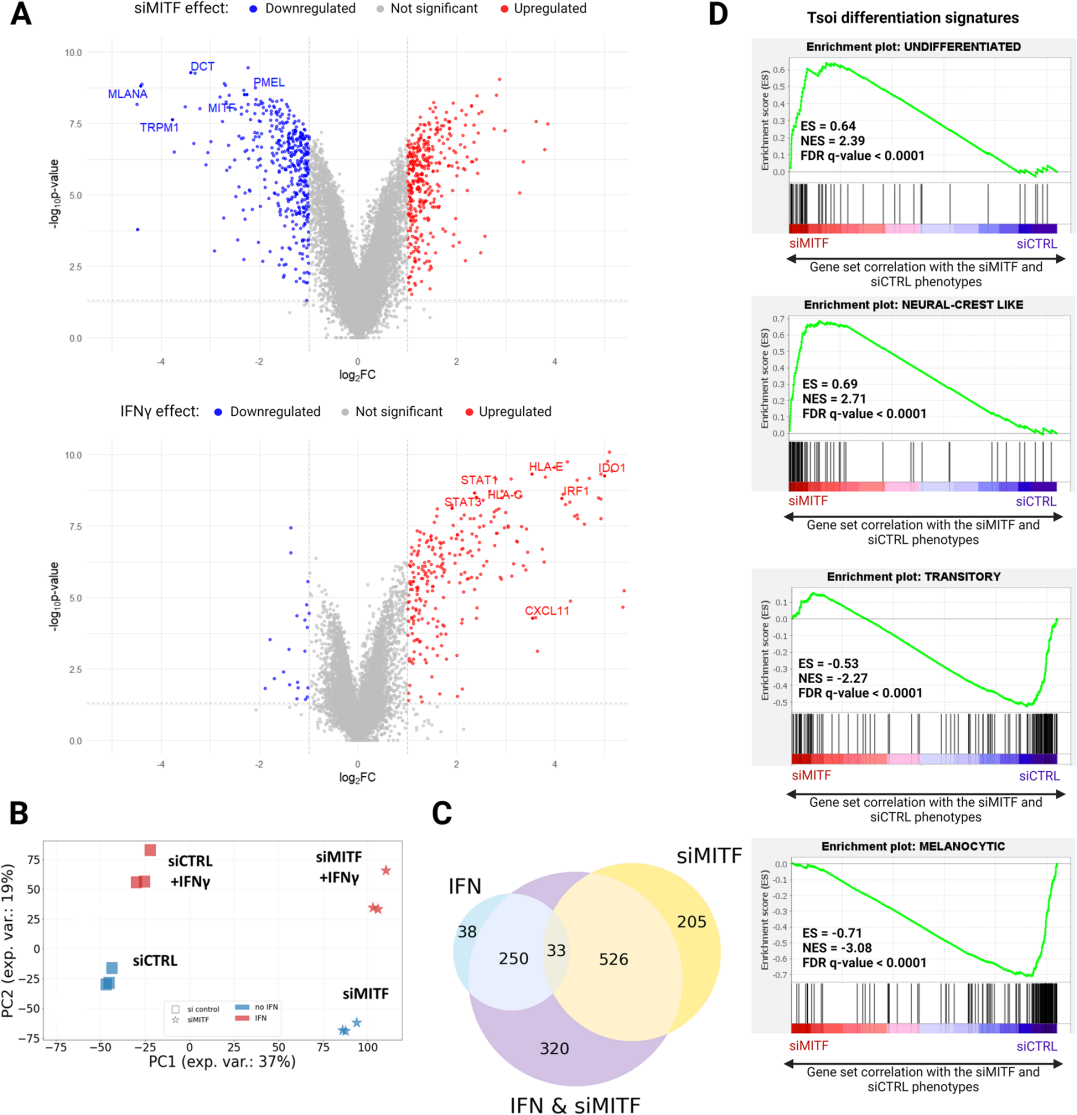
MITF knockdown induces dedifferentiation in 624Mel melanoma cells. **A** Volcano plots of differentially expressed genes, showing the independent effects of siMITF (top) and IFNγ (bottom) on 624Mel cells. **B** PCA of transcriptome profiles according to different experimental conditions. **C** Overlap of differentially expressed genes depending on responses to either MITF knockdown or IFNγ treatment, or to the concomitant factors of MITF knockdown and IFNγ treatment. **D** GSEA comparing the expression of the Tsoi differentiation gene sets in siMITF treated cells compared to the siCTRL treated cells

Dedifferentiated melanoma cells have been reported to display enhanced responses to IFNγ stimulation when compared to differentiated melanoma cells. This response is characterized by an increase in both the expression of interferon response genes and access to chromatin regions [4]. Since IFNγ induces both the expression of immunostimulatory and immunosuppressive genes [27], we set out to investigate the potential effect of melanoma dedifferentiation on IFNγ-induced gene expression, particularly on immune related genes. To do that, we induced dedifferentiation in 624Mel melanoma cells via siRNA mediated MITF knockdown and treated the cells with IFNγ and then performed bulk RNA sequencing on both differentiated (siCTRL) and dedifferentiated (siMITF) 624Mel cells, with or without IFNγ treatment. Treatment with siMITF resulted in significant downregulation of MITF itself and multiple MITF target genes, e.g., *MLANA*, *DCT*, *PMEL* and *TRPM1* (Fig. 1A). Similarly, treatment with IFNγ induced expression of a multitude of known IFN response genes, e.g., *STAT1*, *STAT3*, *IRF1*, *CXCL11*, *IDO1*, *HLA-E* and *HLA-C* (Fig. 1A). This collectively indicates that the siMITF and IFNγ treatments independently produce their intended effects in the 624Mel cells. Principal component analysis (PCA) on substantially expressed genes (top 2,000 expressed genes) confirmed that the siCTRL and siMITF treated 624Mel cells have distinct transcriptional states at baseline, and when treated with IFNγ (Fig. 1B). Notably, a subset of 320 genes respond only to the concomitant treatment of MITF knockdown and IFNγ treatment (Fig. 1C). To further characterize the effect of MITF knockdown and IFNγ treatment on the differentiation status of 624Mel cells, we performed GSEA using established gene sets that specifically associate with differentiation stages in melanoma cells (Tsoi et al. 2018)[8]. Irrespective of IFNγ treatment, the siMITF treated cells showed increased expression of the “Undifferentiated” and “Neural-Crest Like” gene sets, whereas the “Melanocytic” and “Transitory” sets were reduced compared to the siCTRL treated cells, indicating that knocking down MITF expression is sufficient to induce dedifferentiation (Fig. 1C, Supplementary Fig. S1).

**Fig. 2:**
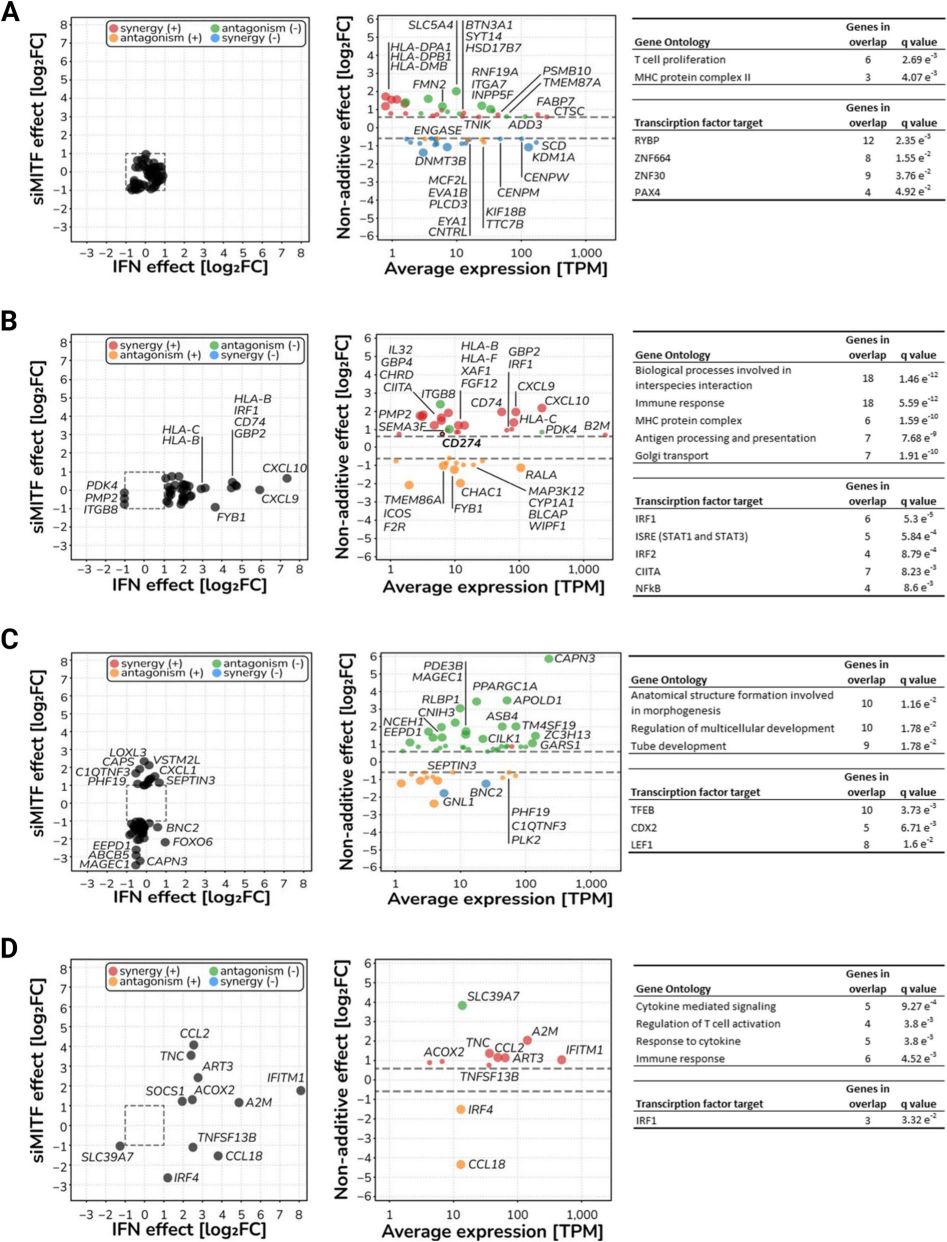
Dedifferentiation and IFNγ stimulation synergistically affect expression of immune related genes in 624Mel melanoma cells. **A** Non additive response emerging from single-effect non-responders (52 genes total). **B** Non additive response emerging from IFNγ responders (36 genes total). **C** Non additive response emerging from siMITF responders (49 genes total). **D** Non additive response emerging from IFNγ & siMITF responders (11 genes total)

To further elucidate how dedifferentiation affects the IFNγ response in melanoma cells, we applied a directional interaction classification system, conceptualized by Piggott et al. (2015) [41], and gene ontology enrichment analysis to our quantified transcriptional profiles. We identified a total of 148 genes showing significant non-additive responses upon combined treatment of siMITF and IFNγ (likelihood ratio test, corrected *P* < 0.05). This response emerges from genes that respond to neither factor (Fig. 2A), from genes that respond to IFNγ only (Fig. 2B), siMITF only (Fig. 2C) or both factors, but whose concomitant response is different from the sum of responses to each factor (Fig. 2D). Importantly, in all cases except for the siMITF single effect responders, the directional interaction of IFNγ and MITF knockdown was predominantly characterized by positive synergism, with non-additive responders showing higher expression than the sum of single effects of IFNγ and MITF knockdown. After functional enrichment analysis on this non-additive response gene set, eight out of 13 enriched GO terms represent cellular processes implicated in adaptive immunity: “T cell proliferation”, “MHC protein complex II”, “Immune response”, “MHC protein complex”, “Antigen processing and presentation”, “Cytokine mediated signaling”, “Regulation of T cell activation” and “Response to cytokine”. Furthermore, an analysis of transcription factor target enrichment revealed increased transcriptional activity for several of the JAK/STAT pathway components, namely STAT1, STAT3, IRF1, IRF2 and CIITA. Notably, among the non-additive response genes was *CD274* (Fig. 2B), the gene encoding for PD-L1. These results collectively indicate that dedifferentiation drives melanoma cells into a distinct cellular state. Upon IFNγ stimulation, this state leads to a synergistic increase in the expression of immune-related genes through enhanced JAK/STAT signaling.

### Dedifferentiated 624Mel melanoma cells simulated with IFNγ secrete greater quantities of multiple immunomodulatory cytokines

**Fig. 3:**
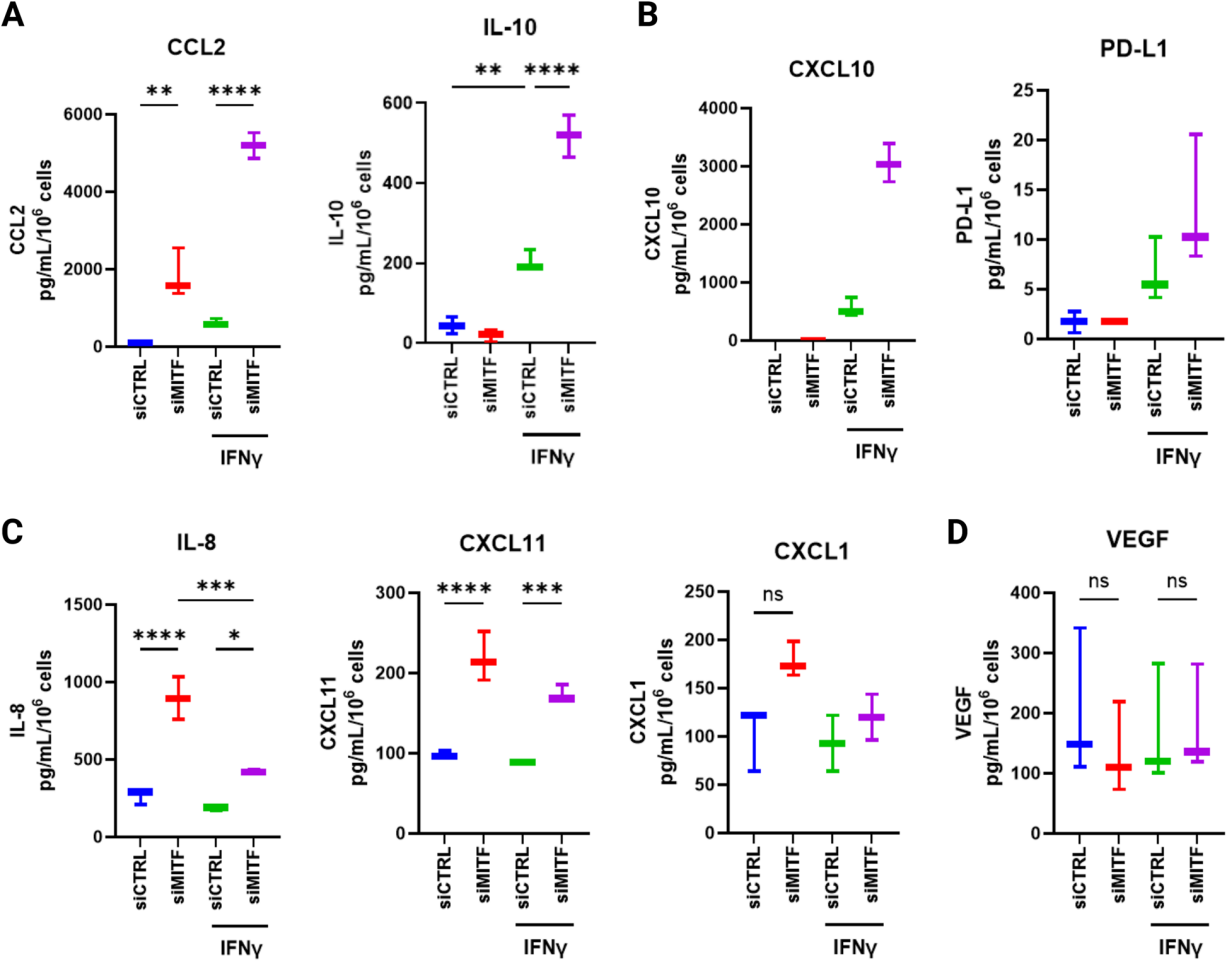
Dedifferentiated 624Mel melanoma cells stimulated with IFNγ secrete greater quantities of multiple immunomodulatory cytokines. **A** Luminex assay measuring secretion (pg/mL/10^6^ cells) of CCL2, IL-10 in 624Mel cells transfected with either siCTRL or siMITF, with or without IFNγ. **B** Luminex measurements for CXCL10 and PD-L1 secretion in 624Mel cells, same conditions as in (A). **C** Luminex measurements for IL-8, CXCL11 and CXCL1 secretion in 624Mel cells, same condition as in (A). **D** Luminex measurement for VEGF secretion in 624Mel cells, same condition as in (A). Plots indicate means along with ranges from minimum to maximum values. Statistical analysis performed by one-way ANOVA and Tukey’s multiple comparisons test, adjusted *P* value * = < 0.05, ** = < 0.01, *** = < 0.001, **** = < 0.0001 (*n *= 3, for CCL2 in the siMITF + IFNγ group, *n* = 2)

Dedifferentiated melanoma cells have been reported to secrete multiple cytokines in greater quantity, notably CCL2, CCL5 and IL-1β, either without inflammatory stimuli or when stimulated with TNFα [2, 18]. As our RNAseq data showed a synergistic transcriptional response on cytokine expression by IFNγ stimulated dedifferentiated 624Mel cells, we investigated the secretome of dedifferentiated 624Mel cells by measuring a panel of 18 cytokines and chemokines using the Luminex Discovery assay. Out of the 18 analytes, 13 were detected. CCL2 and IL-10 secretion was significantly increased (one-way ANOVA, *P* < 0.0001) in dedifferentiated 624Mel cells upon IFNγ treatment (Fig. 3A). Notably, a single value for CCL2 in the siMITF + IFNγ group measured above the assays standard curve and was thus not included in the statistical analyses. CXCL10 and PD-L1 also displayed a synergistic pattern (Fig. 3B). However, the expression values for CXCL10 and PD-L1 were extrapolated beyond the standard curves of the assay, with CXCL10 values for the siMITF + IFNγ group measuring above the standard curve whilst all values for PD-L1 measured below it. Additionally, secretion of IL-8, CXCL11 and CXCL1 was increased in dedifferentiated 624Mel cells, with IL-8 and CXCL11 being significantly increased but not CXCL1 (one-way ANOVA, *P* < 0.0001, P = 0.084 for CXCL1) (Fig. 3C). However, these analytes did not show a pattern of synergistic increase upon IFNγ treatment (Fig. 3C). Lastly, VEGF secretion was detected in all groups but did not differ significantly in any of the experimental conditions (Fig. 3D). Other detected analytes were mostly extrapolated below the standard curves of the assay (data not shown due to values falling below the lower limits of detection). These results indicate that dedifferentiation predisposes 624Mel melanoma cells to increase IFNγ-induced cytokine secretion.

### Synergistic increase in PD-L1 expression in dedifferentiated 624Mel melanoma cells upon IFNγ stimulation

**Fig. 4:**
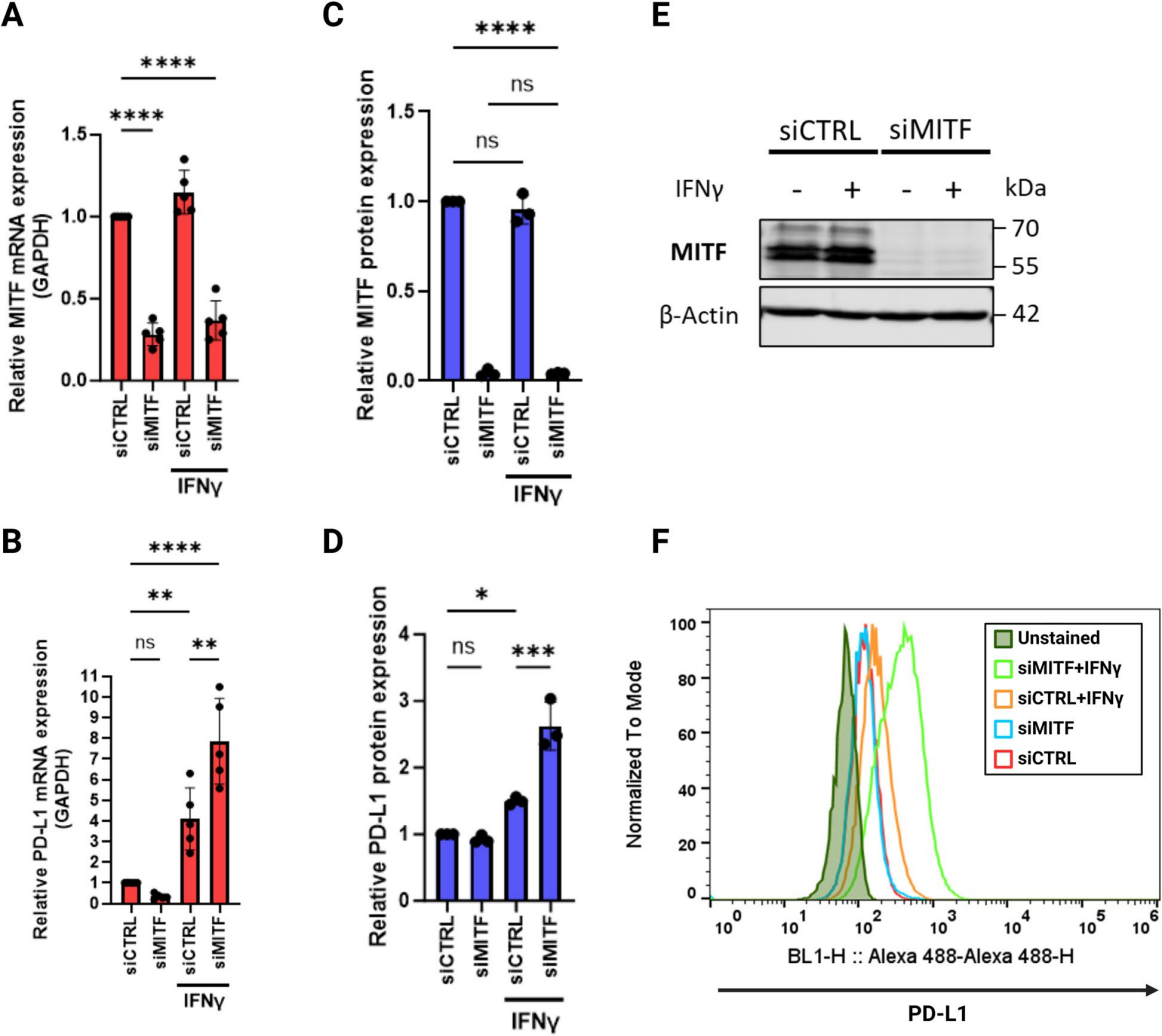
Dedifferentiated 624Mel melanoma cells express more PD-L1 upon IFNγ stimulation. Quantitative RT-PCR of MITF (**A**) and PD-L1 (**B**) mRNA expression in 624Mel cells transfected with Control siRNA (siCTRL) or siMITF, with or without 5 ng/mL IFNγ. **C**, **E** Western blotting of MITF and β-Actin as control in 624Mel cells transfected with siCTRL or siMITF, with or without 5 ng/mL IFNγ. **D**, **F** FACS analysis of PD-L1 protein expression on 624Mel cells transfected with siCTRL or siMITF, with or without 5 ng/mL IFNγ. Plots indicate mean ± standard deviations of value distributions. Statistical analysis performed by one-way ANOVA and Tukey’s multiple comparisons test, adjusted *P* value * = < 0.05, ** = < 0.01, *** = < 0.001, **** = < 0.0001 (*n* = 5 for A & B, *n* = 3 for C & D)

Since PD-L1 is a clinically relevant immunotherapy marker [45], we decided to further investigate this pattern of synergistic increase in PD-L1 expression. As shown in Fig. 4A-D, siRNA mediated MITF knockdown in 624Mel melanoma cells in combination with IFNγ (5 ng/mL) treatment significantly increased PD-L1 expression at both the RNA (Fig. 4B; one-way ANOVA, *P* < 0.01) and protein levels (Fig. 4D; one-way ANOVA, *P* < 0.001) compared to the expected increase caused by IFNγ treatment alone. Critically, without IFNγ, MITF knockdown led to a non-significant decrease in PD-L1 mRNA expression whereas protein expression remained unchanged.

**Fig. 5:**
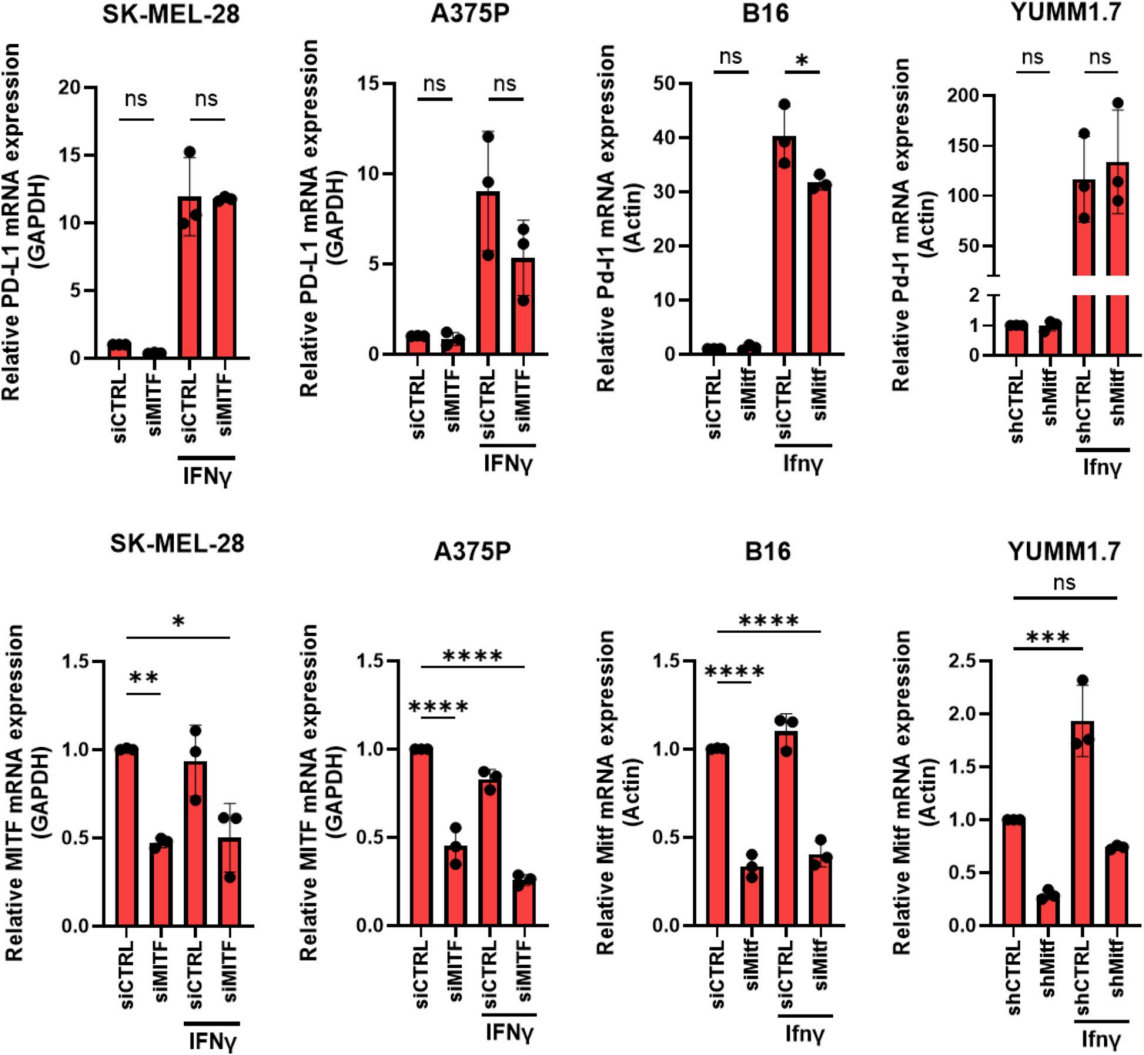
IFNγ/Ifnγ-induced PD-L1 mRNA expression is not synergistic following MITF/Mitf knockdown in SK-MEL-28, A375P, B16 and YUMM1.7 melanoma cells. Graphs showing the relative expression of MITF/Mitf and PD-L1/Pd-l1 as determined by qPCR in the indicated cell lines upon MITF/Mitf knockdown and IFNγ treatment. Plots indicate mean ± standard deviations of value distributions. Statistical analysis performed by one-way ANOVA and Tukey’s multiple comparisons test, adjusted *P* value * = < 0.05, ** = < 0.01, *** = < 0.001, **** = < 0.0001 (*n* = 3)

Next, we investigated the generalizability of this pattern of PD-L1 expression. Contrary to our expectations, when we analyzed the effects of MITF/Mitf knockdown on the IFNγ/Ifnγ-induced PD-L1/Pd-l1 mRNA expression in additional melanoma cell lines, including two mouse melanoma cell lines (B16 and YUMM1.7) for interspecies comparison, we did not observe the same effects as seen in the 624Mel melanoma cell line. In fact, MITF/Mitf knockdown followed by IFNγ/Ifnγ treatment resulted in reduced PD-L1/Pd-l1 mRNA expression in the human A375P and mouse B16 cells lines, whereas Ifnγ treatment significantly increased Mitf expression in the mouse YUMM1.7 cells (Fig. 5). Similarly, MITF knockdown in Malme-3M cells did not result in increased PD-L1 protein expression following IFNγ treatment (Supplementary Fig. S3). This clearly indicates that increased PD-L1 expression following IFNγ treatment and MITF knockdown, as well as the crosstalk between IFNγ and MITF expression in melanoma cells, is context-dependent.

### IFNγ-induced PD-L1 expression in 624Mel melanoma cells is dependent on JAK activity irrespective of differentiation state

**Fig. 6:**
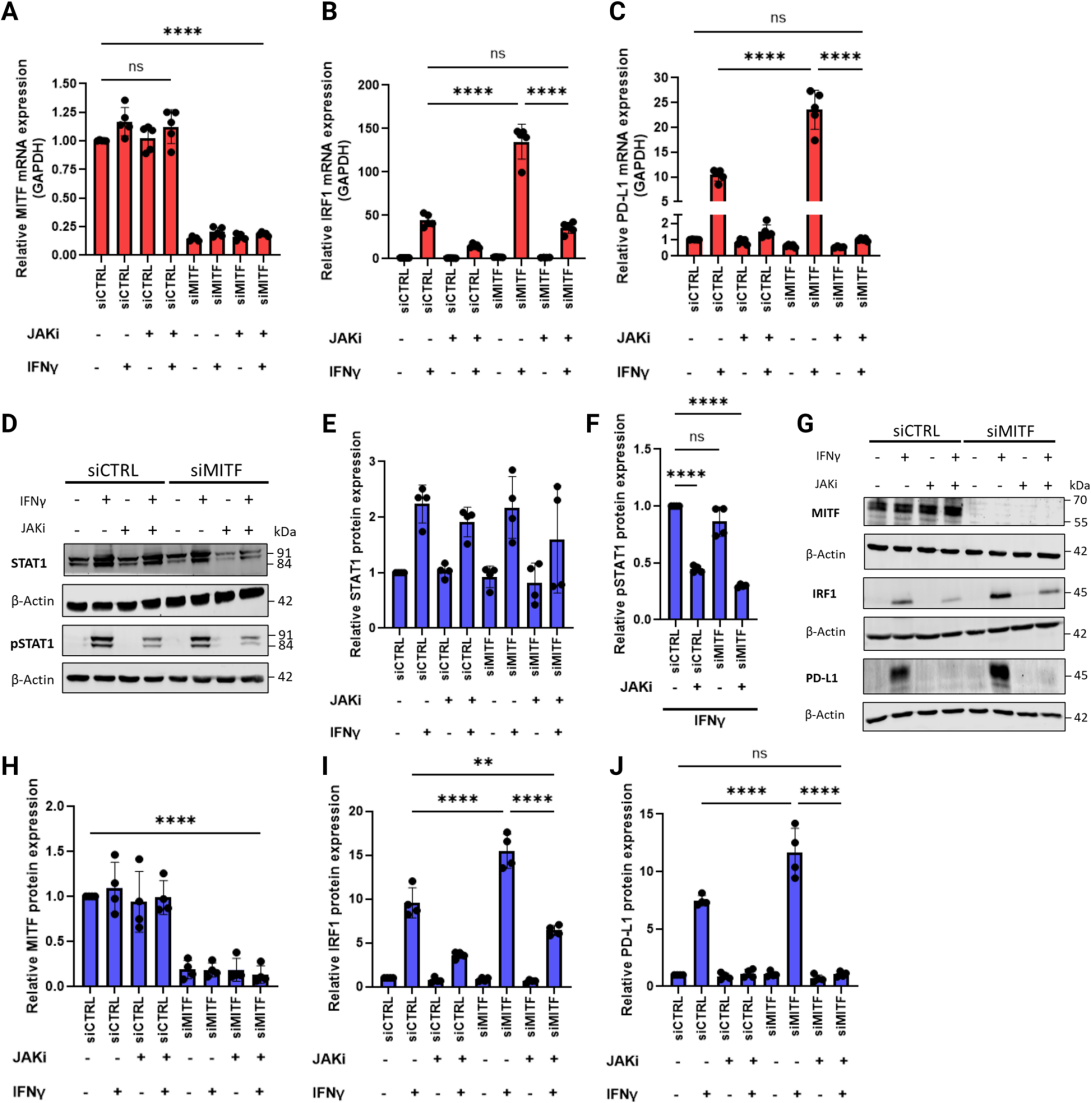
IFNγ-induced PD-L1 expression in 624Mel cells is dependent on JAK kinase activity irrespective of differentiation. **A**, **B**, **C** Quantitative RT-PCR of MITF, IRF1 and PD-L1 mRNA expression in 624Mel cells transfected with control siRNA (siCTRL) or siMITF, with or without 5 ng/mL IFNγ and/or 300 nM JAK inhibitor. **D**, **E**, **F** Western blotting of STAT1, pSTAT1 (Tyr701) and β-Actin in 624Mel cells transfected with siCTRL or siMITF, with or without 5 ng/mL IFNγ and/or 900 nM JAK inhibitor. **G**, **H**, **I**, **J** Western blotting of MITF, IRF1, PD-L1 and β-Actin in 624Mel cells transfected with siCTRL or siMITF, with or without 5 ng/mL IFNγ and/or 900 nM JAK inhibitor. Plots indicate mean ± standard deviations of value distributions. Statistical analysis performed by one-way ANOVA and Tukey’s multiple comparison test, adjusted *P*value ** = < 0.01, *** = < 0.001, **** = < 0.0001 (*n* = 5 for A-C, *n* = 4 for E-J)

Since IFNγ-induced PD-L1 expression is mediated through the JAK1/2-STAT1/3-IRF1 axis in melanoma cells [36], and since *IRF1* appeared among the most significantly affected genes in our RNA sequencing analysis (Fig. 2B), we investigated whether this non-additive increase in PD-L1 expression was dependent on the JAK-STAT1-IRF1 pathway. We began by applying a JAK inhibitor to our experiments in addition to the IFNγ treatment. The JAK kinases are immediately downstream of the IFNγ receptor complex and upon activation the JAKs phosphorylate the STAT transcription factors, inducing the expression of interferon stimulated genes (ISGs) [46–48]. As seen in Fig. 6A-J, inhibiting JAK activity significantly decreased STAT1 phosphorylation (Fig. 6D & F; one-way ANOVA, *P* < 0.0001), leading to decreased IRF1 mRNA and protein expression (Fig. 6B, G & I) and complete abrogation of IFNγ-induced PD-L1 expression in 624Mel cells, irrespective of MITF expression (Fig. 6C, G & J).

### IFNγ-induced PD-L1 expression in 624Mel melanoma cells is dependent on STAT1 irrespective of differentiation state

**Fig. 7:**
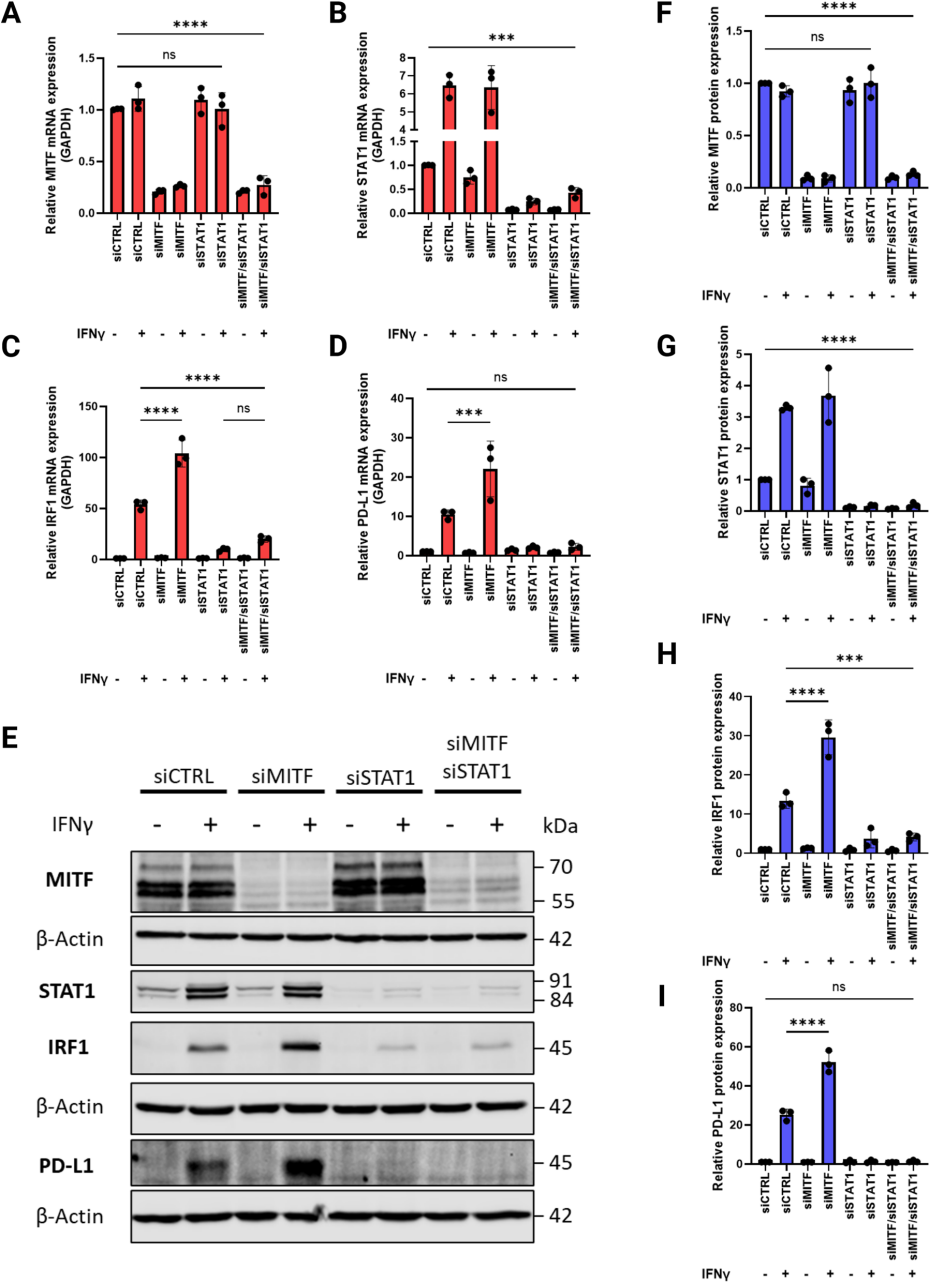
IFNγ-induced PD-L1 expression is dependent on STAT1, irrespective of differentiation status. **A**, **B**, **C**, **D** Quantitative RT-PCR of MITF, STAT1, IRF1 and PD-L1 mRNA expression in 624Mel cells transfected with siCTRL, siMITF, siSTAT1 or both siMITF and siSTAT1, with or without 5 ng/mL IFNγ. **E**, **F**, **G**, **H**, **I** Western blotting of MITF, STAT1, IRF1, PD-L1 and β-Actin in 624Mel cells transfected with siCTRL, siMITF, siSTAT1 or both siMITF and siSTAT1 with or without 5 ng/mL IFNγ. Plots indicate mean ± standard deviations of value distributions. Statistical analysis performed by one-way ANOVA and Tukey’s multiple comparisons test, adjusted *P* value ** = < 0.01, *** = < 0.001, **** = < 0.0001 (n = 3)

Since high IFNγ-induced PD-L1 expression in dedifferentiated 624Mel cells is dependent on JAK activity, we next wanted to identify which of the STAT members downstream of JAK is involved specifically. It has been reported that STAT1 and STAT3 are the main regulators of IFNγ-induced PD-L1 expression [36]. As seen in Fig. 7A-I, siRNA-mediated knockdown of STAT1 led to significantly decreased IRF1 expression (Fig. 7C & H, one-way ANOVA, *P* < 0.001) and complete abrogation of IFNγ-induced PD-L1 expression in 624Mel cells, irrespective of MITF expression (Fig. 7D & I). This is similar to the results seen when applying the JAK inhibitor (Fig. 6J). Whilst NF-κB inhibition led to reduced MITF expression in 624 Mel cells, inhibition of neither STAT3 nor NF-κB impacted PD-L1 expression in IFNγ-induced cells. (Supplementary Fig. S4).

### High IFNγ-induced PD-L1 expression in dedifferentiated 624Mel melanoma cells is dependent on IRF1

**Fig. 8:**
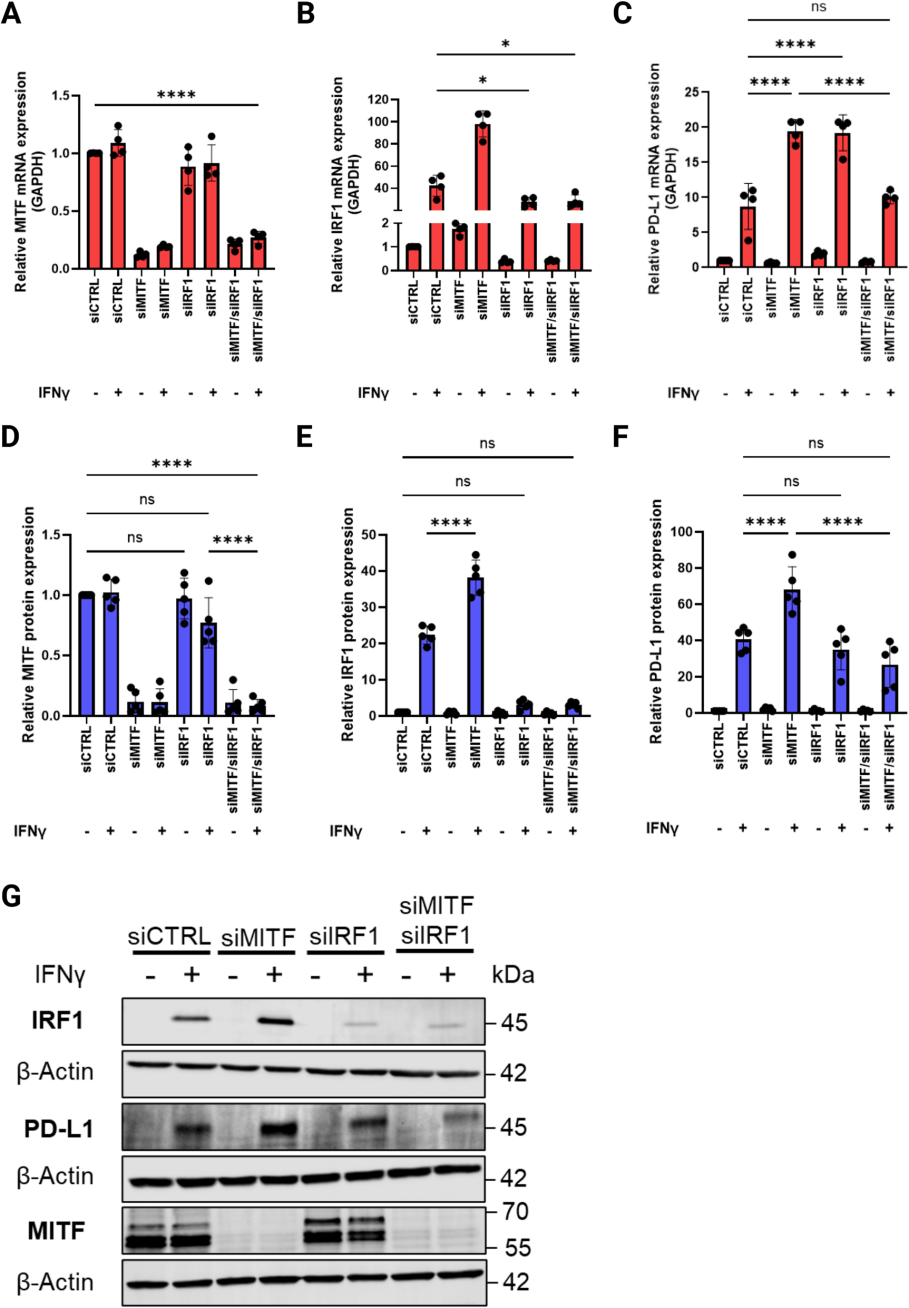
IFNγ-induced PD-L1 expression in dedifferentiated melanoma cells is dependent on IRF1**. A**, **B**, **C** Quantitative RT-PCR of MITF, IRF1 and PD-L1 mRNA expression in 624Mel cells transfected with Control siRNA (siCTRL), siMITF, siIRF1 or both siMITF and siIRF1, with or without 5 ng/mL IFNγ. **E**, **F**, **G** Western blotting of MITF, STAT1, IRF1, PD-L1 and β-Actin in 624Mel cells transfected with siCTRL, siMITF, siIRF1 or both siMITF and siIRF1, with or without 5 ng/mL IFNγ. Plots indicate mean ± standard deviations of value distributions. Statistical analysis performed by one-way ANOVA and Tukey’s multiple comparisons test, adjusted *P* value * = < 0.05, ** = < 0.01, *** = < 0.001, **** = < 0.0001 (*n* = 4 for A-C, *n* = 5 for D-G)

Knowing that 624Mel cells are dependent on STAT1 for IFNγ-induced PD-L1 expression, we next examined the role of IRF1 in mediating the high PD-L1 expression in dedifferentiated 624Mel cells. First, we observed that siRNA mediated double knockdown of MITF and IRF1 resulted in reduced mRNA levels of both factors (Fig. 8A-B & Fig. 8D-E). Furthermore, the expression of IRF1 and PD-L1 was synergistically increased in the dedifferentiated 624Mel cells upon IFNγ treatment (Fig. 8E-F). Surprisingly, IFNγ-induced PD-L1 mRNA expression was significantly increased in the siIRF1 group compared to the IFNγ treated control (Fig. 8C; one-way ANOVA, *P* < 0.0001). Notably, this increase was not observed at the protein level, as IFNγ-induced PD-L1 protein expression was not significantly affected by siIRF1 knockdown alone (Fig. 8F). However, double knockdown of MITF and IRF1 decreased IFNγ-induced PD-L1 mRNA and protein expression in the dedifferentiated 624Mel cells, resulting in similar expression levels to that of the IFNγ treated control cells (Fig. 8C & F). This indicates that IRF1 is involved in mediating the high PD-L1 expression observed in IFNγ stimulated dedifferentiated 624Mel cells.

### Patient derived melanoma cell lines recapitulate a context-dependent relationship between dedifferentiation and IFNγ responses

**Fig. 9:**
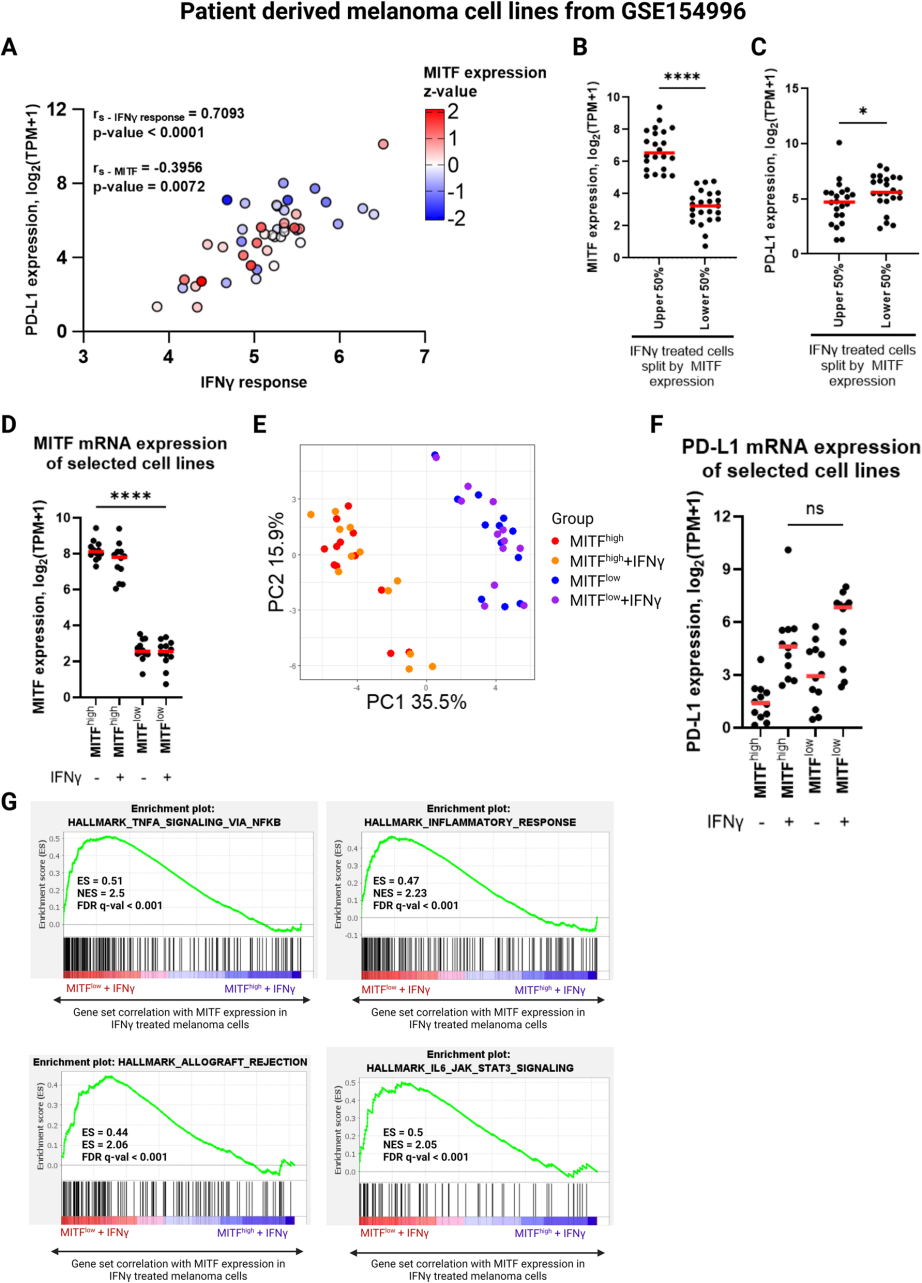
Dedifferentiated melanoma cell lines have higher IFNγ-induced PD-L1 expression and increased immune related gene expression. **A** Scatter plot and Spearman correlation analysis for association of PD-L1 mRNA expression with IFNγ response and MITF expression in 45 patient derived melanoma cell lines. **B**, **C** MITF and PD-L1 expression of IFNγ treated melanoma cell lines when divided by MITF expression. **D** The top quartile (MITF^high^) and bottom quartile (MITF^low^) melanoma cell lines ranked by MITF expression. **E** Principal component analysis of MITF^high^ and MITF^low^ melanoma cells, with or without IFNγ treatment. **F** PD-L1 expression of the MITF^high^ and MITF^low^ melanoma cell lines. **G** GSEA of immunologically associated hallmark gene sets upregulated in IFNγ treated MITF^low^ melanoma cells compared to IFNγ treated MITF^high^ melanoma cells. Lines in scatterplots represent medians. Statistical analysis performed by Spearman correlation analysis (**A**) and Mann–Whitney U test (**B**, **D** & **F**), *P* value * = < 0.05, **** = < 0.0001 (*n* = 22 for upper 50%, *n* = 23 for Lower 50%)

As our results indicate that dedifferentiation affects IFNγ-induced PD-L1 expression in a context-dependent manner in 624Mel cells and is not observed in our other melanoma cell lines (Fig. 4, Supplementary Fig. S3), we asked the question whether this trend is noticeable in a wider context of melanoma cases. Publicly available RNA sequencing data (GSE154996) consisting of 45 patient derived melanoma cell lines, either with or without 6-h treatment with 5 ng/mL IFNγ. Whilst PD-L1 mRNA expression is highly associated with the overall IFNγ response in the dataset (Fig. 9A, Spearman r_s_ = 0.703, *P* < 0.0001) there is also a weak negative association between MITF and IFNγ-induced PD-L1 mRNA expression (Fig. 9A, Spearman r_s_ = -0.3956, P = 0.0072) (Supplementary Fig. S7) [33]. Similarly, ranking these melanoma cell lines by MITF expression (Fig. 9B) revealed significantly higher PD-L1 expression among cell lines with lower MITF levels (Fig. 9C; Mann–Whitney U test, *P* < 0.05), indicating that whilst not universal, there is a tendency for higher IFNγ-induced PD-L1 expression among less differentiated melanoma cells.

To further investigate what may facilitate this difference in PD-L1 expression, we compared the top and bottom quartiles of the melanoma cell lines, ranked by MITF expression (Fig. 9D). These two groups had clearly distinct transcriptional states (Fig. 9E), with the MITF^high^ cells appearing differentiated and the MITF^low^ cells displaying a more dedifferentiated phenotype according to their expression of the Tsoi differentiation signatures (Supplementary Fig. S8).Both groups had appreciable variation in PD-L1 expression. However, most of the cells with high IFNγ-induced PD-L1 expression belonged to the MITF^low^ group (Fig. 9F; Supplementary Fig. S14A), although the difference in median PD-L1 expression did not prove statistically significant (Mann–Whitney U test, P = 0.16). Additionally, the IFNγ treated MITF^low^ cells displayed significantly higher expression of CCL2 and IL-8 (Kruskal Wallis test, *P* < 0.01) (Supplementary Fig. 14B & D). Additionally, CCLE2 mRNA expression was significantly increased in the MITF^low^ group upon IFNγ treatment (two-tailed paired Wilcoxon test, P = 0.0024) (Supplementary Fig. S14C), whilst IL-8 mRNA expression was not significantly affected by IFNγ (Supplementary figure). Similarly, the median expression of CXCL10 and CXCL11 was higher in the MITF^low^ cells, but the difference was not statistically significant (Mann–Whitney U test, P = 0.0780 for CXCL10 and P = 0.1561 for CXCL11) (Supplementary Fig. S14F). However, IL-10 was not widely expressed in neither group of melanoma cell lines (Supplementary Fig. S14G). Gene set enrichment analysis of the hallmark signature gene sets revealed that several immunological gene sets were significantly upregulated in the IFNγ treated MITF^low^ group compared to the IFNγ treated MITF^high^ group (Fig. 9G). However, the IFNγ response signature gene set was not among the most highly significant gene sets, although it appeared upregulated in the MITF^low^ group (Supplementary Fig. S9). Overall, these results indicate that dedifferentiated melanoma cells have a widely enhanced response to IFNγ compared to their differentiated counterparts, resulting in greater expression of a multitude of immunologically relevant genes that are not exclusive to the IFNγ response. This supports our findings in the 624Mel cell line (Fig. 2) and further associates high IFNγ-induced PD-L1 expression with dedifferentiated melanoma cells.

**Fig. 10:**
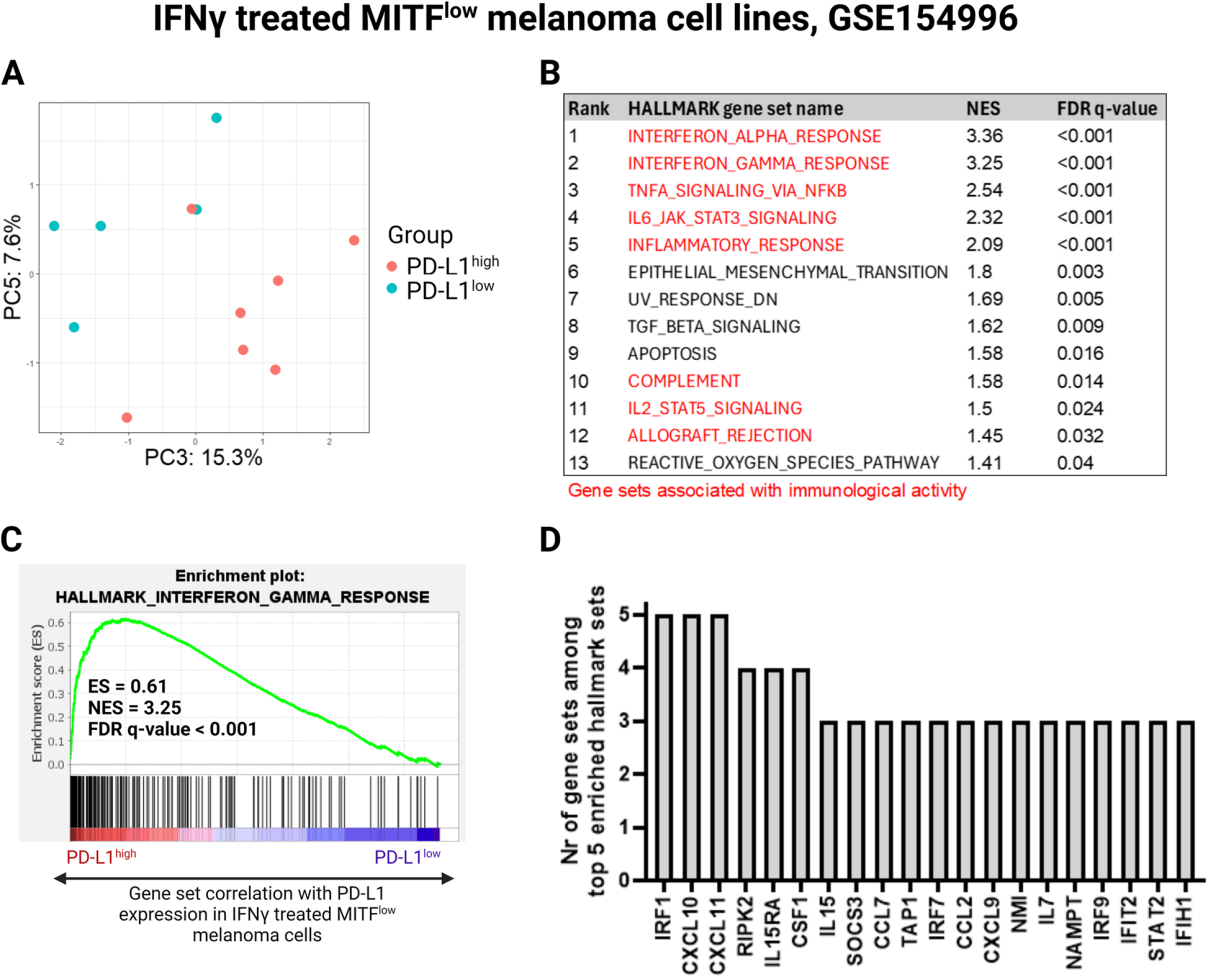
Dedifferentiated melanoma cells with high IFNγ-induced PD-L1 expression are characterized by increased inflammatory signalling that converges on IRF1 expression. **A** PCA of MITF^low^ melanoma cell by high or low IFNγ-induced PD-L1 expression, along PC3 and PC5. **B** Top significantly upregulated hallmark gene sets in the MITF^low^/PD-L1^high^ group compared to the MITF^low^/PD-L1^low^ group. **E** GSEA plot showing enrichment of the IFNγ response gene set in MITF^low^/PD-L1^high^ melanoma cells. **D** Leading edge analysis defining the core set of genes contributing to the enrichment of the top five most significantly upregulated hallmark gene sets in the MITF^low^/PD-L1^high^ group

To explain the molecular context of high PD-L1 expression in the dedifferentiated melanoma cells, we divided the MITF^low^ group into PD-L1^high^ and PD-L1^low^ based on their IFNγ-induced PD-L1 expression (Supplementary Fig. S10A) and then compared those two groups. Based on their overall mRNA expression, correlation between the individual MITF^low^ cell lines ranged from -0.38 to 0.16 and was non-significant in all cases (Supplementary Fig. S10B). However, minor inherent differences in the transcriptional states of the PD-L1^high^ and PD-L1^low^ groups were detected (Fig. 10A). Furthermore, gene set enrichment analysis revealed that the PD-L1^high^ cells have significantly upregulated immunological gene signatures (Fig. 10B), with the top five most highly upregulated hallmark gene sets all belonging to inflammatory signaling, one of which being the IFNγ response signature gene set (Fig. 10C). To identify key genes impacting the PD-L1^high^ group’s gene set signature profile, we conducted a leading-edge subset analysis [42] on the top five most significant gene sets. We identified a core set of 20 genes present in at least three leading-edge subsets (Fig. 10D). Notably, *IRF1* was common to all subsets, indicating its strong influence on the gene set signature profile of the PD-L1^high^ group. Altogether, these results indicate that dedifferentiated melanoma cells tend to be more responsive to IFNγ stimulation, and that high IFNγ-induced PD-L1 expression among dedifferentiated melanoma cells is associated with a broad enhancement of inflammatory signaling where IRF1 expression is a central contributing factor.

A similar comparison within the MITF^high^ group (Supplementary Fig. S12A) revealed that high PD-L1 expression among the MITF^high^ cells was also associated with enrichment of multiple inflammatory gene sets compared to MITF^high^ cells with low PD-L1 expression (Supplementary Fig. S12B). The MITF^high^/PD-L1^high^ cells were also enriched for the Tsoi neural-crest like gene set compared to the MITF^high^/PD-L1^low^ cells, which themselves showed a melanocytic transcriptomic profile (Supplementary Fig. S12C). Lastly, the PD-L1^high^ groups among both MITF^high^ and MITF^low^ cells shared the “STTTCRNTTT_IRF_Q6” gene set as the top upregulated transcription factor target gene set compared to their PD-L1^low^ counterparts (Supplementary Fig. S12D). This is an IRF1 target gene set, consisting of genes containing at least one occurrence of the STTTCRNTTT motif in proximity of their transcription start site [49]. Together, this indicates that whilst PD-L1 expression varies both among MITF^low^ and MITF^high^ cells, high IFNγ-induced PD-L1 expression is generally associated with enhanced inflammatory gene expression (Fig. 10B, Supplementary Fig. S12B). Furthermore, among the MITF^high^ cells specifically, high PD-L1 expression associates with distinct sub-phenotypes that possess features of dedifferentiation as indicated by a more neural-crest like transcriptional profile compared to MITF^high^/PD-L1^low^ cells (Supplementary Fig. S12C).

## Discussion

Dedifferentiation and IFNγ stimulation can each exert contrasting effects on melanoma progression based on the cellular and/or therapeutic context [8, 27]. Furthermore, dedifferentiation can render melanoma cells hypersensitive to the inflammatory cytokine TNFα, causing increased expression of immunomodulatory cytokines [2]. Given that melanoma cells commonly dedifferentiate in response to inflammation [6], and that IFNγ is both vital for effective anti-tumor immune responses and known to induce expression of immunosuppressive proteins in cancer cells [27, 36], it is important to characterize how dedifferentiation modulates the IFNγ response of melanoma.

Our results demonstrate that following dedifferentiation the IFNγ response of melanoma cells gets conditioned, leading to a non-additive increase in IFNγ-induced expression of a subset of genes implicated in adaptive immunity, compared to differentiated melanoma cells. Furthermore, this results in higher PD-L1 expression, mediated through the JAK-STAT1-IRF1 axis, and higher secretion of CCL2, IL-10, CXCL10 and PD-L1 (Fig. 3A-B) although the specific regulatory mechanisms behind the increased cytokine secretions are unknown. However, we only observe this in the 624Mel cell line. Four other melanoma cell lines do not increase PD-L1 mRNA expression following MITF knockdown and IFNγ treatment (Fig. 5). These results indicate that there exists a molecular context which links dedifferentiation to IFNγ sensitivity in melanoma. However, the details of this context remain unclear and require further investigation. It is plausible that melanoma cells at varying stages of differentiation will show different responses to the combination of MITF knockdown and IFNγ stimulation. Indeed, the human melanoma cell lines tested in this study have varying baseline levels of MITF (Supplementary Fig. S6). This indicates that their baseline differentiation status is also different, and that the MITF knockdown applied in our experiments may, by extension, lead to varying degrees of dedifferentiation. Similarly, variations in the efficiency of the MITF knockdown itself (Fig. 5) could also partly explain the varying results observed in our melanoma cell lines. Additionally, our melanoma cell lines also possess different mutational backgrounds according to the Cellosauros database (Supplementary Fig. S13) [50]. This is relevant, as other factors such as p53 are known to affect IFNγ-induced PD-L1 expression in melanoma [51]. By extension, varying p53 levels or activity might influence the extent to which dedifferentiation modulates IFNγ-induced PD-L1 expression in different melanoma cell lines. Mutations in JAK/STAT pathway components could similarly affect the interplay between IFNγ signaling and dedifferentiation, as they can affect the general responsiveness of melanoma cells to IFNγ [33]. For instance, out of the melanoma cell lines used in the present study, the SK-MEL-28 cell line is reported to harbor missense mutations in the *JAK2* and *JAK3* genes, according to the Cancer Cell Line Encyclopedia (CCLE, https://depmap.org/portal) [52, 53]. To which extent these mutations affect IFNγ responsiveness is unclear, although our results indicate that they do not abrogate IFNγ-induced PD-L1 RNA expression (Fig. 5). Further studies deciphering the exact molecular context that facilitates this mechanism would help determine its prevalence in melanoma patients and therefore it would help determine its potential clinical relevance.

Observing increased IFNγ-induced PD-L1 expression in the dedifferentiated 624Mel cells is partly in line with previous studies where SOX10 knockdown, which reduces MITF expression in melanoma cells, led to increased IFNγ-induced PD-L1 expression in three out of four tested melanoma cell lines[21]. Similarly, Subhadarshini et al. (2023) recently developed a dynamic model of phenotypic plasticity and IFNγ signaling in melanoma which predicted a synergistic relationship between dedifferentiation and IFNγ induced PD-L1 mRNA expression [37]. This model associates the transcription factors SOX10, MITF, JUN, ZEB1 and SOX9 with both basal and IFNγ-induced PD-L1 expression, through their regulation of melanoma differentiation. This indicates that dedifferentiated melanoma cells are more likely to display high PD-L1 mRNA expression than differentiated ones [37]. Our results support these findings, as our MITF knockdown experiments produced similar results for PD-L1 mRNA expression (Fig. 4B). However, to our knowledge, this is the first study to directly link MITF expression alone with the IFNγ response in melanoma cells.

Previous studies have demonstrated that IFNγ-induced PD-L1 expression is reliant on JAK1/JAK2 activity, and that PD-L1 promoter activity and subsequent expression is mainly influenced by STAT1/STAT3 and IRF1 in IFNγ treated melanoma cells [33, 36]. Our results are in most part consistent with these studies. However, IRF1 knockdown caused an unexpected increase in PD-L1 mRNA expression and did not significantly affect PD-L1 protein expression in the differentiated 624Mel cells (Fig. 8C & F). This is unexpected, as IRF1 is the main transcription factor driving PD-L1 promoter function in melanoma cells [36], and thus IRF1 knockdown would be anticipated to drastically decrease PD-L1 expression in melanoma cells, irrespective of differentiation state. Nonetheless, the IRF1 knockdown employed in this study clearly abrogates the increase in IFNγ-induced PD-L1 expression seen in the dedifferentiated 624Mel cells (Fig. 8F), indicating that this increase is indeed dependent on the JAK-STAT1-IRF1 axis.

We see indications that dedifferentiated melanoma cells secrete greater quantities of several cytokines that have previously been implicated in melanoma. Increased and synergistic CCL2 expression and secretion have been demonstrated in dedifferentiated melanoma cells and linked to senescence induction in melanoma cells in vitro and myeloid cell recruitment in melanoma tumors in vivo [2, 18]. Furthermore, MITF knockdown leads to decreased CXCL10 expression and a subsequent decrease in immune cell infiltration in B16F10 allografts [54]. Likewise, small fold changes in IL-8 and IL-10 mRNA expression following dedifferentiation in melanoma cells have been reported [18]. However, to our knowledge, this is the first study reporting a synergistic increase in IFNγ-induced secretion of CCL2, IL-10 and CXCL10 following dedifferentiation. Whilst the Luminex values for PD-L1 portrayed the same pattern observed in our other assays of PD-L1 expression (for example, Fig. 4D), we still cannot conclude that PD-L1 was being secreted, as it was extrapolated below the standard curve of the Luminex assay. Increased secretion of IL-8 and CXCL11 following MITF knockdown alone are a novel finding in melanoma. Pretreatment serum levels of CXCL11 have been linked to poor overall survival in patients with metastatic melanoma treated with anti-CTLA-4 ICI therapy [55]. IL-10 has been implicated in driving tumor associated macrophage (TAM) polarization and has been linked to increased myeloid derived suppressor cell (MDSC) infiltration in melanoma [56, 57]. Lastly, IL-8 has been described as a potential autocrine growth factor in melanoma, supporting proliferation and conferring metastatic potential to melanoma cells in vitro and in vivo [58, 59]. Given the complex and often pluripotent effects that cytokines exert in the TME, it is difficult to extrapolate these in vitro observations to the *in* vivo environment. Furthermore, since IL-10 does not seem widely expressed across different melanoma cell lines (Supplementary Fig. S14G), the increased secretion noted in the 624Mel cell line needs to be considered as an exception.

Analysis of the GSE154996 data set suggests that dedifferentiated melanoma cells actually show altered responses to IFNγ, characterized by enhanced inflammatory signaling (Fig. 9). This is consistent with what we observed in the dedifferentiated 624Mel cells (Fig. 2). Furthermore, the results suggest that a subgroup of dedifferentiated melanoma cells expresses increased levels of PD-L1, CCL2 and IL-8 in response to IFNγ, with IRF1 expression contributing highly to the upregulated inflammatory signaling responses of PD-L1^high^ expressing cells (Fig. 10, Supplementary Fig. S10, Supplementary Fig. S14). This supports our idea of a context-dependent relationship between differentiation levels and PD-L1 expression in melanoma cells and indicates that this molecular context exists in melanoma patients. However, it remains unclear which specific factors facilitate this mechanism. Comparison of the IFNγ treated MITF^high^ and MITF^low^ groups with regards to transcription factor target (TFT) gene sets from MSigDB revealed significant increase of multiple gene sets associated with the SRF, AP-1 and NF-κB transcription factors (Supplementary Fig. S9). This molecular profile has been associated with the MITF^low^ (or dedifferentiated) melanoma cell state [2]. Similarly, multiple TFT gene sets associated with STAT and IRF transcription factors appeared upregulated in the dedifferentiated PD-L1^high^ cells compared to PD-L1^low^ cells (Supplementary Fig. S11). However, only one of those gene sets was significantly enriched. This was an IRF1 target gene set (STTTCRNTTT_IRF_Q6), which implicates increased IRF1 activity with high PD-L1 expression in dedifferentiated melanoma cells, similar to what we observe in the 624Mel cell line. Additionally, the main genes contributing to the observed TFT gene set profile in the MITF^low^/PD-L1^high^ cells included *NRP1* and *IRF2* (Supplementary Fig. S11). NRP1 is a transmembrane glycoprotein that has been shown to affect IFNγ signaling in endothelial cells [60], and is furthermore negatively regulated by MITF in melanoma cells [16]. However, whether NRP1 affects IFNγ signaling in melanoma cells is unknown.

Our work has several limitations. First, the molecular results all derive from monocultures of melanoma cell lines, making it impossible to draw any direct conclusions about the biological significance of the increased PD-L1 expression following dedifferentiation nor that of the observed cytokines. It would require either in vivo studies using immunocompetent models or sophisticated in vitro assays utilizing co-cultures of melanoma cells and cytotoxic immune cells to properly test whether enhanced inflammatory gene expression in dedifferentiated melanomas facilitates immune evasion. Second, our in vitro results mainly rely on a single cell line, which limits their generalizability. And whilst the *in-silico* analysis of the GSE154996 dataset provided support for our in vitro results, it was not fully consistent with the 624Mel cell line. Third, despite our results, the molecular context linking dedifferentiation and IFNγ signaling remains incompletely characterized. Further studies on which factors facilitate the transcriptional states presented here and their associated epigenetic context would help identify both the proper models for researching the phenomenon further as well as its prevalence among melanoma patients.

Overall, whilst not revealing a major unique facilitator of these contextually altered IFNγ responses in dedifferentiated melanoma cells, our analysis shows that this phenomenon is likely to exist in a wider context of melanoma cells, and that it is mostly associated with the upregulation of inflammatory signaling as well as IRF1 expression and activity following IFNγ stimulation. Our results thus provide a rationale that differentiation may serve as proxy for IFNγ responsiveness in melanomas. Furthermore, targeting differentiation could help shape melanoma immunogenicity by modulating the IFNγ signaling of melanoma cells.

## Conclusions

In summary, our results show that melanoma dedifferentiation influences IFNγ signaling in a context-dependent manner. Dedifferentiation enhances the expression of immunomodulatory genes in response to IFNγ, increases PD-L1 protein expression, and promotes secretion of multiple cytokines. These findings suggest that targeting differentiation could potentially modulate IFNγ signaling in melanoma.

## Supplementary Information

Supplementary Material 1. Table S1. qPCR primers used for the publication.

Supplementary Material 2. Fig. S1 GSEA of siCTRL+IFNγ and siMITF+IFNγ 624Mel cells for the melanoma differentiation signatures generated by Tsoi et al(8), using z values derived from log_2_(TPM + 1) transformed expression values from our 624Mel RNA sequencing data.

Supplementary Material 3. Fig. S2 Additional biological replicates underlying results shown in Figure 3. A) Western blotting for MITF. B) FACS for PD-L1, all biological replicates, including secondary only control run (upper left).

Supplementary Material 4. Fig. S3 Combination of MITF knockdown and IFNγ treatment does not result in increased PD-L1 protein expression in Malme-3M melanoma cells. (A, B, C) Western blotting of MITF, PD-L1 and β-Actin in Malme-3M cells transfected with siCTRL or siMITF, with or without 5 ng/mL IFNγ. Plots indicate mean +/- standard deviations of value distributions. Statistical analysis performed by one-way ANOVA and Tukey’s multiple comparisons test, adjusted P value ** = < 0.01, **** =< 0.0001 (n = 3).

Supplementary Material 5. Fig. S4 Preliminary results of the effect of NF-κB inhibition (QNZ) and STAT3 inhibition (Stattic) on IFNγ-induced PD-L1 expression in 624Mel cells. Quantitative RT-PCR of MITF (A), IRF1 (B) and PD-L1 (C) mRNA expression in siCTRL and siMITF 624Mel cells treated with IFNγ and QNZ or Stattic (n = 1). Plots indicate mean +/- standard deviations of value distributions of three technical replicates. 

Supplementary Material 6. Fig. S5 Additional biological replicates underlying the results shown in Figures 6, 7 and 8. A) Western blotting for STAT1, pSTAT1, MITF, IRF1 and PD-L1 using Actin as loading control, corresponding to Figure 6. B) Western blotting for MITF, STAT1 IRF1 and PD-l1 using Actin as loading control, corresponding to Figure 7. C) Western blotting for IRF1, PD-L1 and MITF using Actin as loading control, corresponding to Figure 8.

Supplementary Material 7. Fig. S6 Baseline MITF protein expression in the human melanoma cell lines used in the study, with or without 5 ng/mL IFNγ. Western blotting for MITF using Actin as loading control (left) and relative expression levels of each cell line corrected for loading (right) (n = 1).

Supplementary Material 8. Fig. S7 Simple linear regression analysis of the association between MITF expression and either basal PD-L1 expression (left) or IFNγ-induced PD-L1 expression in 45 patient derived melanoma cell lines form the GSE 154996 data set.

Supplementary Material 9. Fig. S8 (A) MITF expression of the untreated wild-type melanoma cell lines from the GSE154996 data set, blue dots indicate the cell lines selected for the MITF^low^ group whereas red dots indicate the MITF^high^ group. (B) GSEA comparing the MITF^high^ and MITF^low^ groups, with and without IFNγ treatment, with regards to the Tsoi melanoma differentiation signature gene sets.

Supplementary Material 10. Fig. S9 GSEA results showing the top 20 and top 28 upregulated hallmark (left) and transcription factor target gene (right) sets, respectively, in the IFNγ treated MITF^low^ group compared to the IFNγ treated MITF^high^ group. Coloured TFT gene sets are associated with transcription factors previously linked to the MITF^low^ melanoma cell state.

Supplementary Material 11. Fig. S10 (A) Graph and table showing the categorization of MITF^low^ melanoma cells into either PD-L1^high^ or PD-L1^low^. (B) Pearson correlation matrix comparing the selected IFNγ treated MITF^low^ melanoma cell lines based on overall mRNA expression, adjusting for multiple testing with Bonferroni correction. (C) PD-L1 expression in the different PD-L1 groups of MITF^low^ cells, statistical analysis performed by Statistical analysis performed by Mann-Whitney U test, P value ** =< 0.01 (n = 5 for PD-L1^low^, n = 7 for PD-L1^high^). (D) Principal component analysis (same analysis as presented in figure 10B) comparing MITF^low^ cells based on their PD-L1 expression, showing no apparent segregation or clustering of the two groups along PC1 and PC2.

Supplementary Material 12. Fig. S11 (A) Top 20 upregulated gene transcription factor target gene sets in the PD-L1^high^ group when compared to the PD-L1^low^ group. (B) Overlap of the top leading-edge subsets of the top ten gene sets presented in (A). (C) Most commonly shared genes among the leading-edge subsets in question, colour gradient depicts a genes contribution to the respective subset.

Supplementary Material 13. Fig. S12 (A) MITF (left) and PD-L1 expression (right) among the MITFhigh melanoma cell group after categorizing by PD-L1 expression, statistical analysis performed by Mann-Whitney U test, P value ** = < 0.01 (n = 8 for PD-L1^low^, n = 4 for PD-L1^high^). (B) Top 10 hallmark gene sets upregulated in the MITF^high^/PD-L1^high^ cells compared to the MITF^high^/PD-L1^low^ cells. (C) Tsoi differentiation signature gene set enrichment in the MITF^high^/PD-L1^high^ cells compared to the MITF^high^/PD-L1^low^ cells. (D) Enrichment plots of the “STTTCRNTTT_IRF_Q6” TFT gene set regulation in MITF^high^/PD-L1^high^ cells compared to the MITF^high^/PD-L1^low^ cells (left) and in MITF^low^/PD-L1^high^ cells compared to the MITF^low^/PD-L1^low^ cells (right).

Supplementary Material 14. Fig. S13 Mutational status of the melanoma cell lines used in this study, according to the Cellosaurus database. The mouse melanoma cell line B16 has no sequence variations listed in the database.

Supplementary Material 15. Fig. S14 Comparison of *IRF1*,*CCL2, CXCL10, CXCL11, IL-8 and* *CD274* gene expression across the MITF^high^ and MITF^low^ melanoma cell lines from the GSE15449 dataset. (A) Heatmaps depicting overall gene expression across the two groups, either untreated (upper heatmap) or with IFNγ treatment (lower heatmap). (B) CCL2 mRNA expression across all conditions. (C) Paired comparison of CCL2 mRNA expression in the MITF^low^ melanoma cell lines. (D) IL-8 mRNA expression across all conditions. (E) Paired comparison of CCL2 mRNA expression in the MITF^low^ melanoma cell lines. (F) Comparison of mRNA expression of CXCL10, CXCL11 and IRF1 across all conditions. (G) IL-10 mRNA expression in every individual cell line belonging to either the MITF^high^ or MITF^low^ group, with or without IFNγ treatment. Lines in scatterplots represent medians. Statistical analysis performed by Kruskal-Wallis and Dunn’s multiple comparisons test (B & E), two-tailed paired Wilcoxon test (C & E) or Mann-Whitney U test (F), adjusted *P* value ** = < 0.01, **** = < 0.0001 (*n *= 12).

Supplementary Material 16. Uncropped WB figures.

## Data Availability

The RNA-sequencing data for this publication are accessible through GEO Series accession number GSE283655 (https://www.ncbi.nlm.nih.gov/geo/query/acc.cgi?acc=GSE283655).

## Abbreviations

AP-1: Activator protein 1
CIITA: Class II major histocompatibility complex transactivator
CCL2: C–C motif chemokine ligand 2
CCL5: C–C motif chemokine ligand 5
CCL27: C–C motif chemokine ligand 27
CXCL1: CXC motif chemokine ligand 1
CXCL2: CXC motif chemokine ligand 2
CXCL9: CXC motif chemokine ligand 9
CXCL10: CXC motif chemokine ligand 10
CXCL11: CXC motif chemokine ligand 11
DCT: Dopachrome tautomerase
EDTA: Ethylenediaminetetraacetic acid
FACS: Fluorescence activated cell sorting
FDR: False discovery rate
FPKM: Fragments per kilobase of transcript per million mapped reads
GAPDH: Glyceraldehyde 3-phosphatase dehydrogenase
GSEA: Gene set enrichment analysis
HLA-E: Major histocompatibility complex class 1 E
HLA-C: Major histocompatibility complex class 1 C
IDO1: Indoleamine 2,3-dioxygenase 1
IFNβ: Interferon beta
IFNγ: Interferon gamma
IL-2: Interleukin 2
IL-7: Interleukin 7
IL-8: Interleukin 8
IL-10: Interleukin 10
IL-21: Interleukin 21
IL-27: Interleukin 27
IRF1: Interferon regulatory factor 1
IRF2: Interferon regulatory factor 2
ISGs: Interferon stimulated genes
JAK: Janus kinase
MDSC: Myeloid derived suppressor cell
MITF: Microphthalmia-associated transcription factor
MLANA: Melanoma antigen recognized by T cells
MSigDB: Molecular signatures database
NF-κB: Nuclear factor kappa-B
NRP1: Neuropilin 1
PCA: Principal component analysis
PD-L1: Programmed death-ligand 1
PMEL: Premelanosome protein
qPCR: Quantitative polymerase chain reaction
shRNA: Short hairpin RNA
siRNA: Short interfering RNA
SRF: Serum response factor
STAT1: Signal transducer and activator of transcription 1
STAT3: Signal transducer and activator of transcription 3
TAM: Tumor associated macrophage
TFT: Transcription factor target
TME: Tumor microenvironment
TNFα: Tumor necrosis factor alpha
TPM: Transcripts per million
TRPM1: Transient receptor potential cation channel subfamily M member 1.
VEGF: Vascular endothelial growth factor.
