# Supplementary figures and images for: Differentiation status determines the effects of IFNγ on the expression of PD-L1 and immunomodulatory genes in melanoma
Source: Cell Commun Signal. 2024 Dec 31;22:618. doi: 10.1186/s12964-024-01963-6 (PMC11687009; doi:10.1186/s12964-024-01963-6)

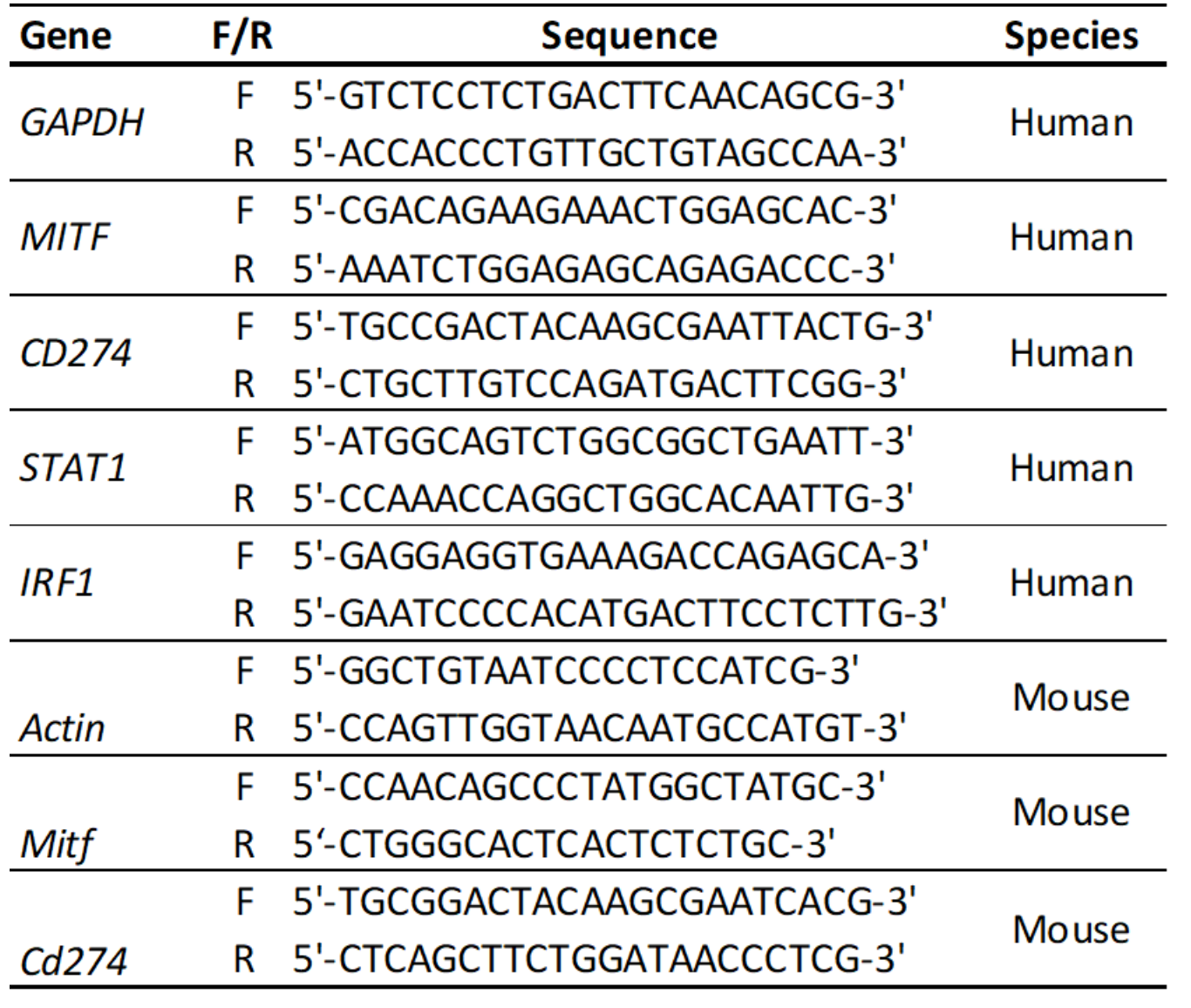

Supplement: Supplementary file 1 — Supplementary Material 1. Table S1. qPCR primers used for the publication. [file 12964_2024_1963_MOESM1_ESM.png]

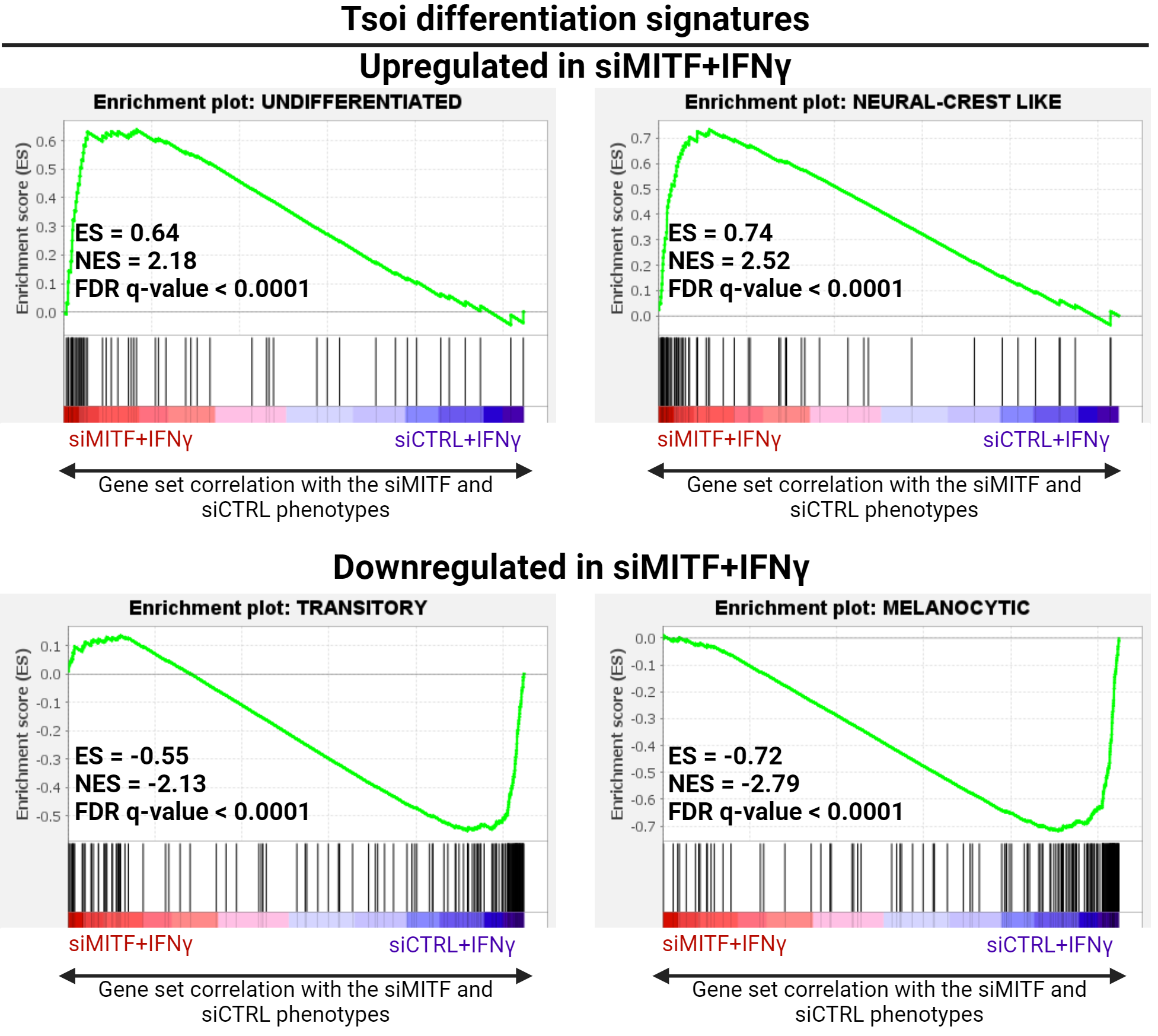

Supplement: Supplementary file 2 — Supplementary Material 2. Fig. S1 GSEA of siCTRL+IFNγ and siMITF+IFNγ 624Mel cells for the melanoma differentiation signatures generated by Tsoi et al(8), using z values derived from log2(TPM + 1) transformed expression values from our 624Mel RNA sequencing data. [file 12964_2024_1963_MOESM2_ESM.png]

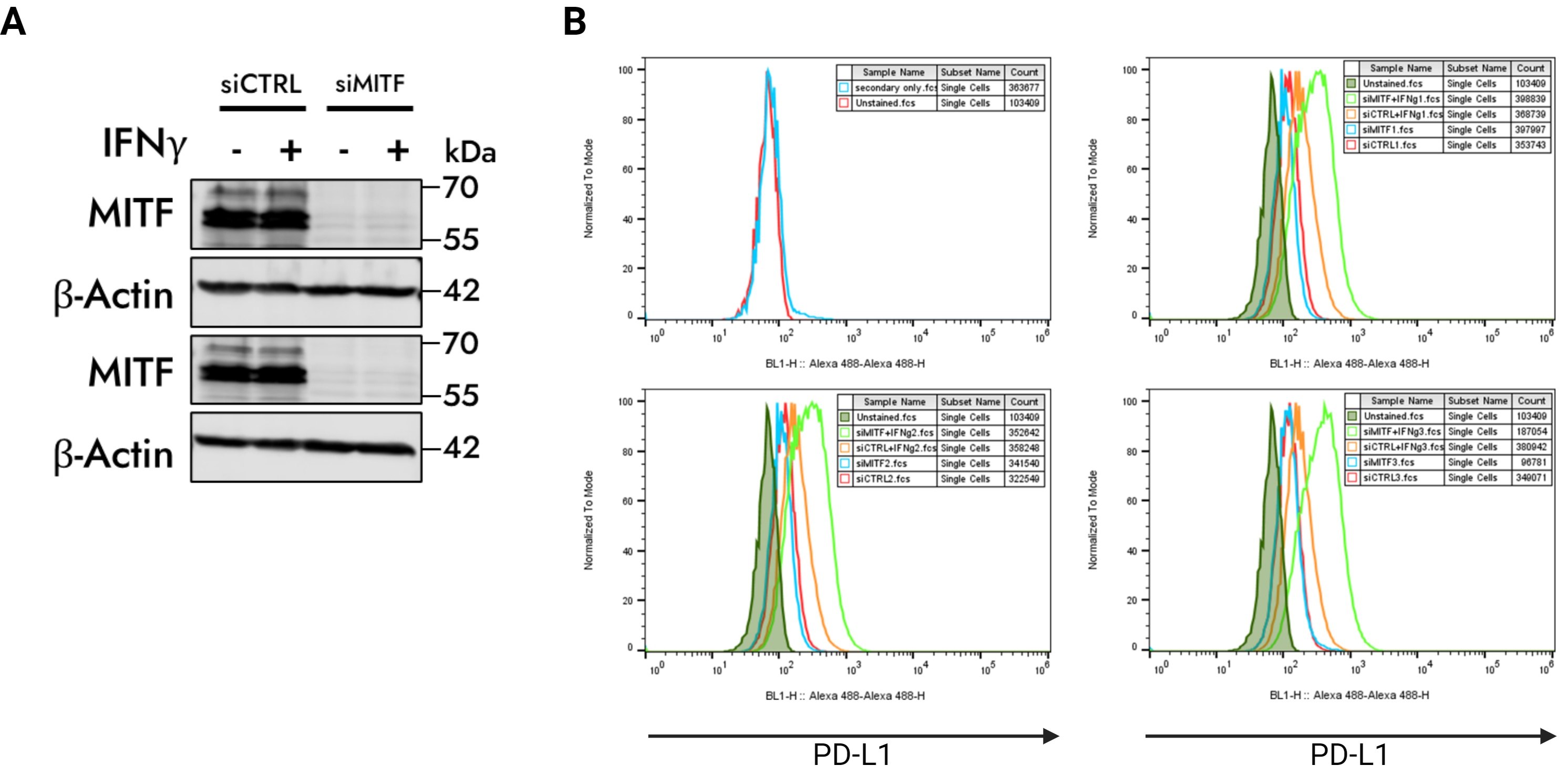

Supplement: Supplementary file 3 — Supplementary Material 3. Fig. S2 Additional biological replicates underlying results shown in Figure 3. A) Western blotting for MITF. B) FACS for PD-L1, all biological replicates, including secondary only control run (upper left). [file 12964_2024_1963_MOESM3_ESM.png]

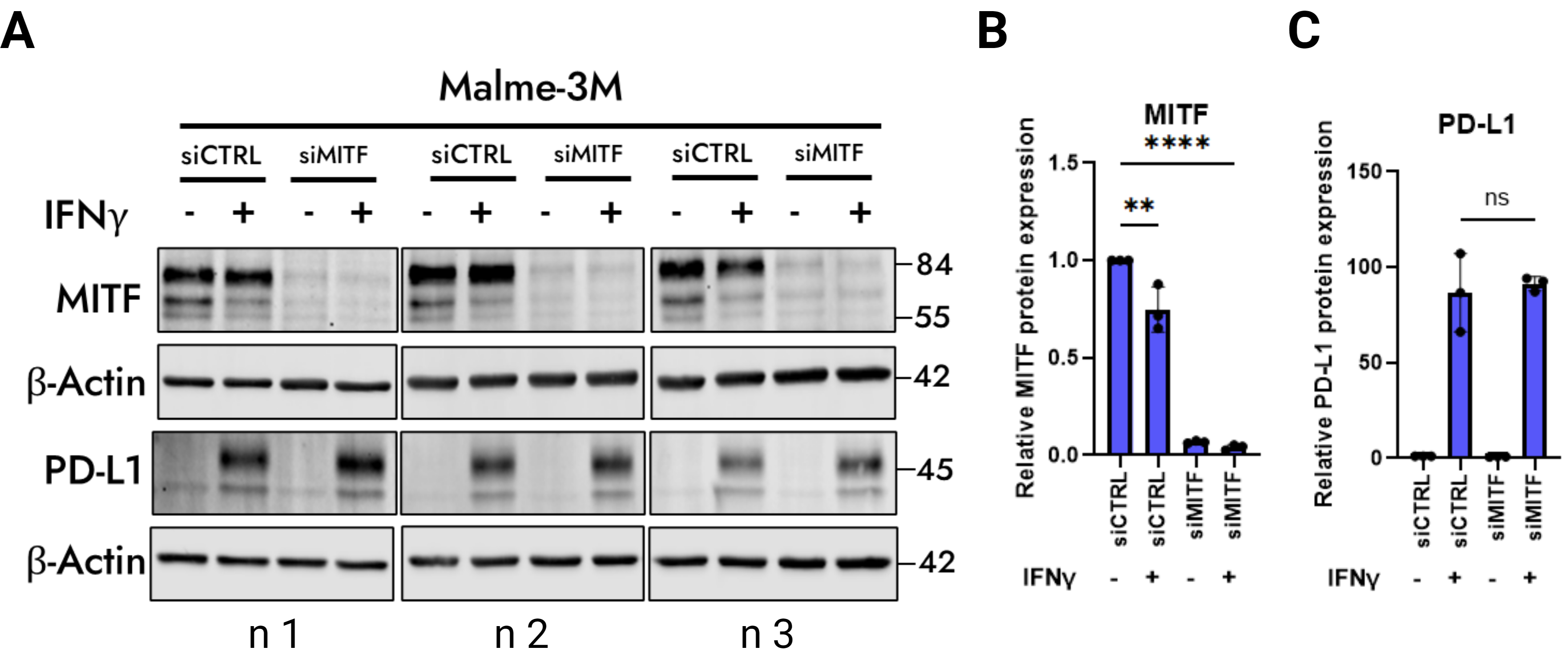

Supplement: Supplementary file 4 — Supplementary Material 4. Fig. S3 Combination of MITF knockdown and IFNγ treatment does not result in increased PD-L1 protein expression in Malme-3M melanoma cells. (A, B, C) Western blotting of MITF, PD-L1 and β-Actin in Malme-3M cells transfected with siCTRL or siMITF, with or without 5 ng/mL IFNγ. Plots indicate mean +/- standard deviations of value distributions. Statistical analysis performed by one-way ANOVA and Tukey’s multiple comparisons test, adjusted P value ** = < 0.01, **** =< 0.0001 (n = 3). [file 12964_2024_1963_MOESM4_ESM.png]

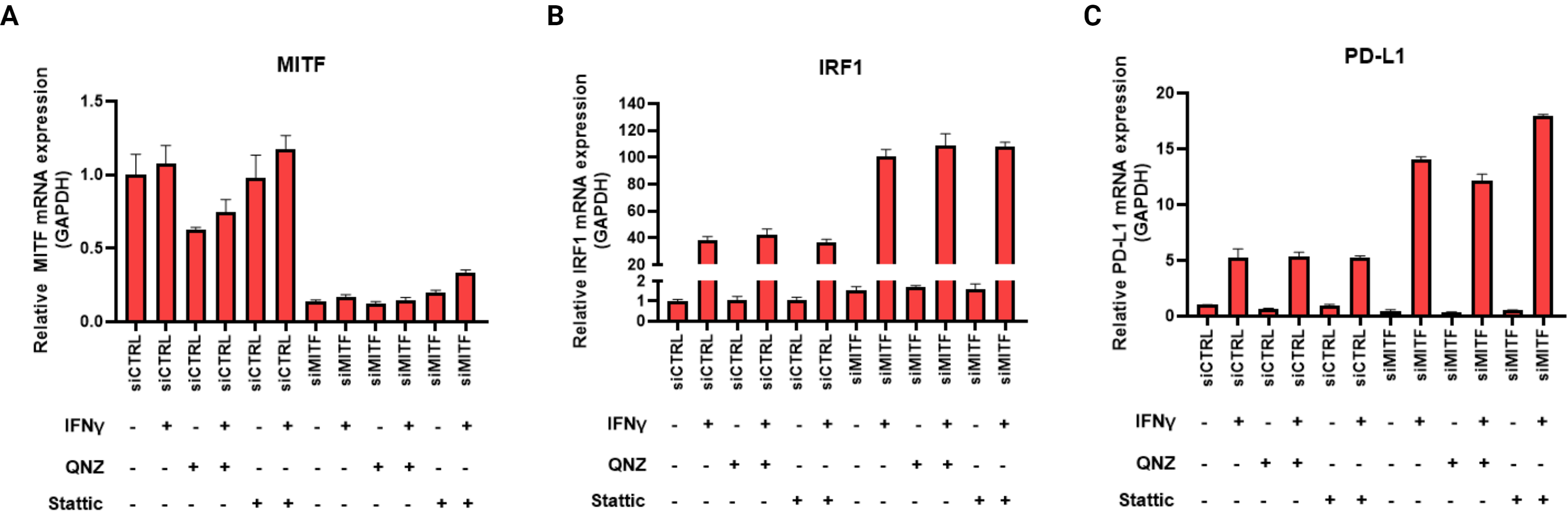

Supplement: Supplementary file 5 — Supplementary Material 5. Fig. S4 Preliminary results of the effect of NF-κB inhibition (QNZ) and STAT3 inhibition (Stattic) on IFNγ-induced PD-L1 expression in 624Mel cells. Quantitative RT-PCR of MITF (A), IRF1 (B) and PD-L1 (C) mRNA expression in siCTRL and siMITF 624Mel cells treated with IFNγ and QNZ or Stattic (n = 1). Plots indicate mean +/- standard deviations of value distributions of three technical replicates. [file 12964_2024_1963_MOESM5_ESM.png]

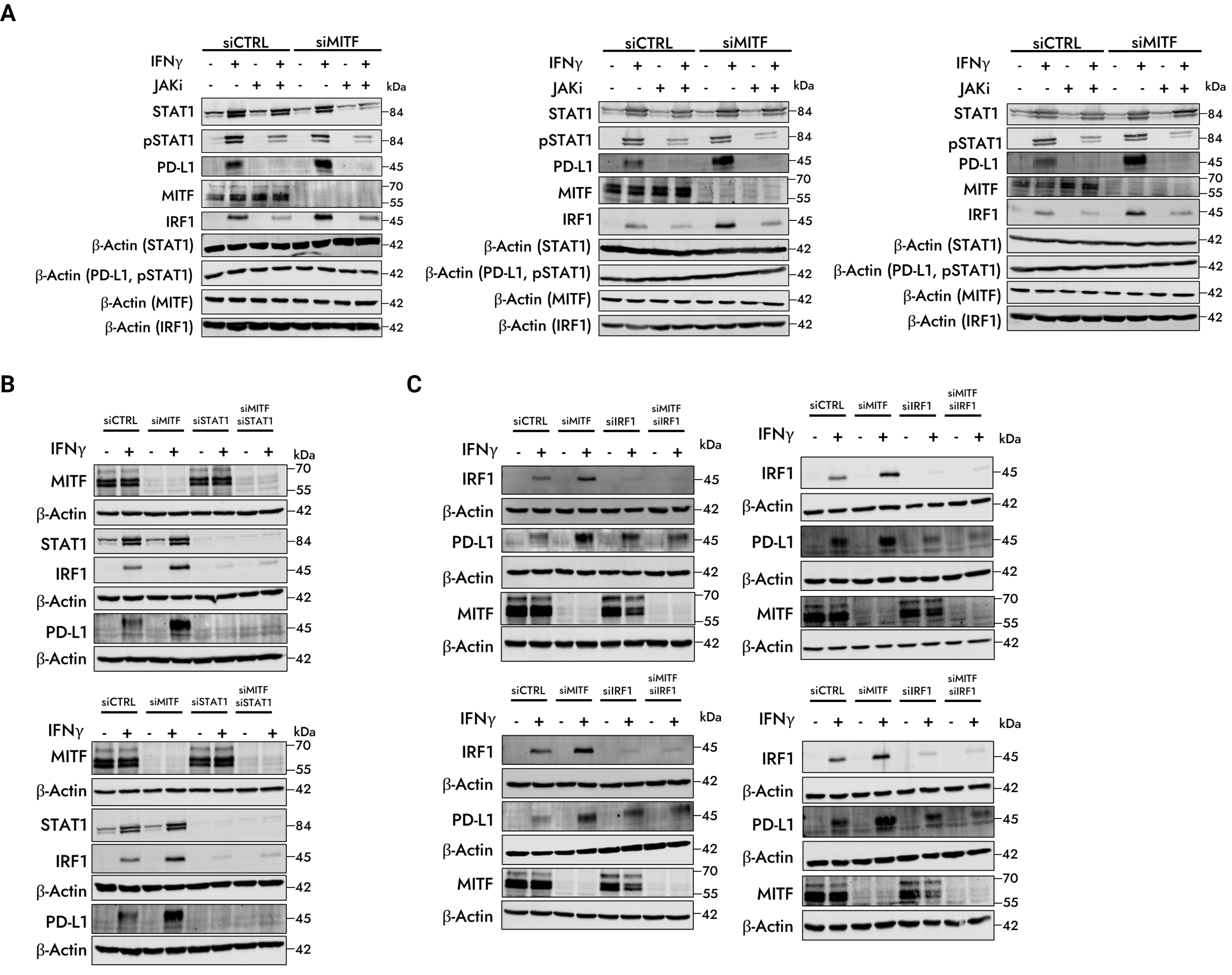

Supplement: Supplementary file 6 — Supplementary Material 6. Fig. S5 Additional biological replicates underlying the results shown in Figures 6, 7 and 8. A) Western blotting for STAT1, pSTAT1, MITF, IRF1 and PD-L1 using Actin as loading control, corresponding to Figure 6. B) Western blotting for MITF, STAT1 IRF1 and PD-l1 using Actin as loading control, corresponding to Figure 7. C) Western blotting for IRF1, PD-L1 and MITF using Actin as loading control, corresponding to Figure 8. [file 12964_2024_1963_MOESM6_ESM.png]

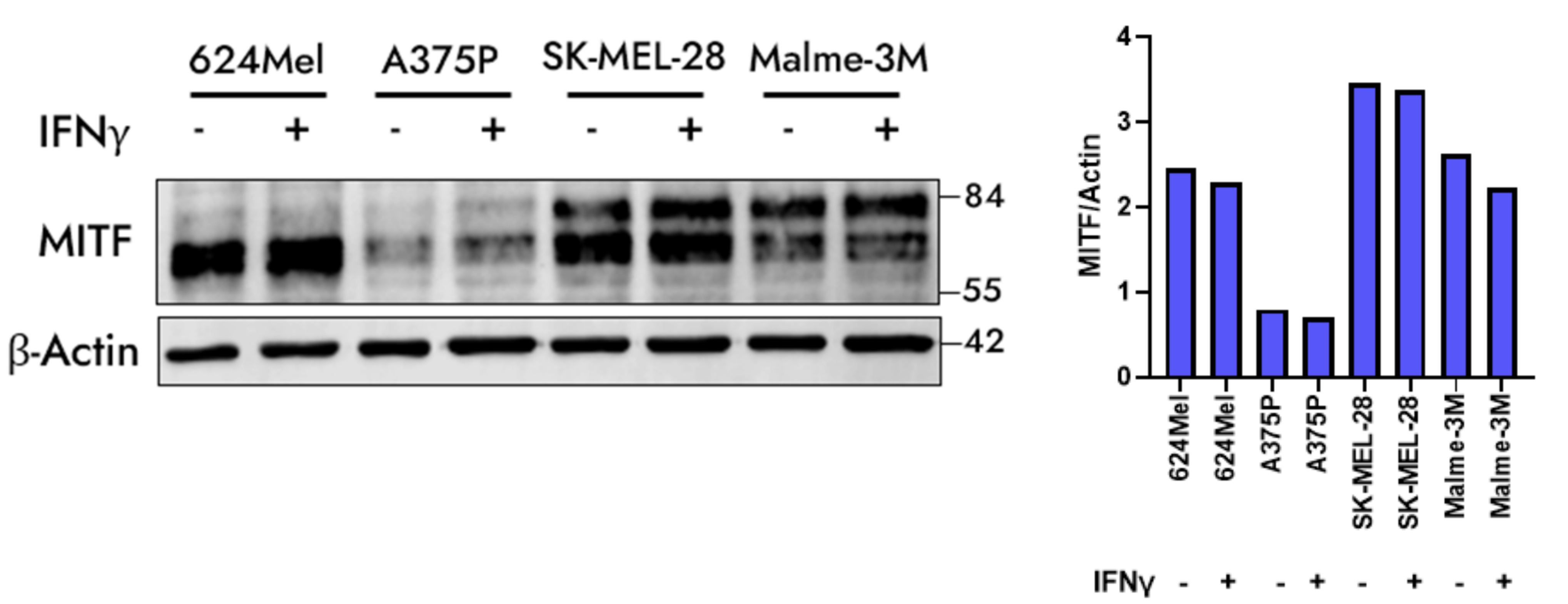

Supplement: Supplementary file 7 — Supplementary Material 7. Fig. S6 Baseline MITF protein expression in the human melanoma cell lines used in the study, with or without 5 ng/mL IFNγ. Western blotting for MITF using Actin as loading control (left) and relative expression levels of each cell line corrected for loading (right) (n = 1). [file 12964_2024_1963_MOESM7_ESM.png]

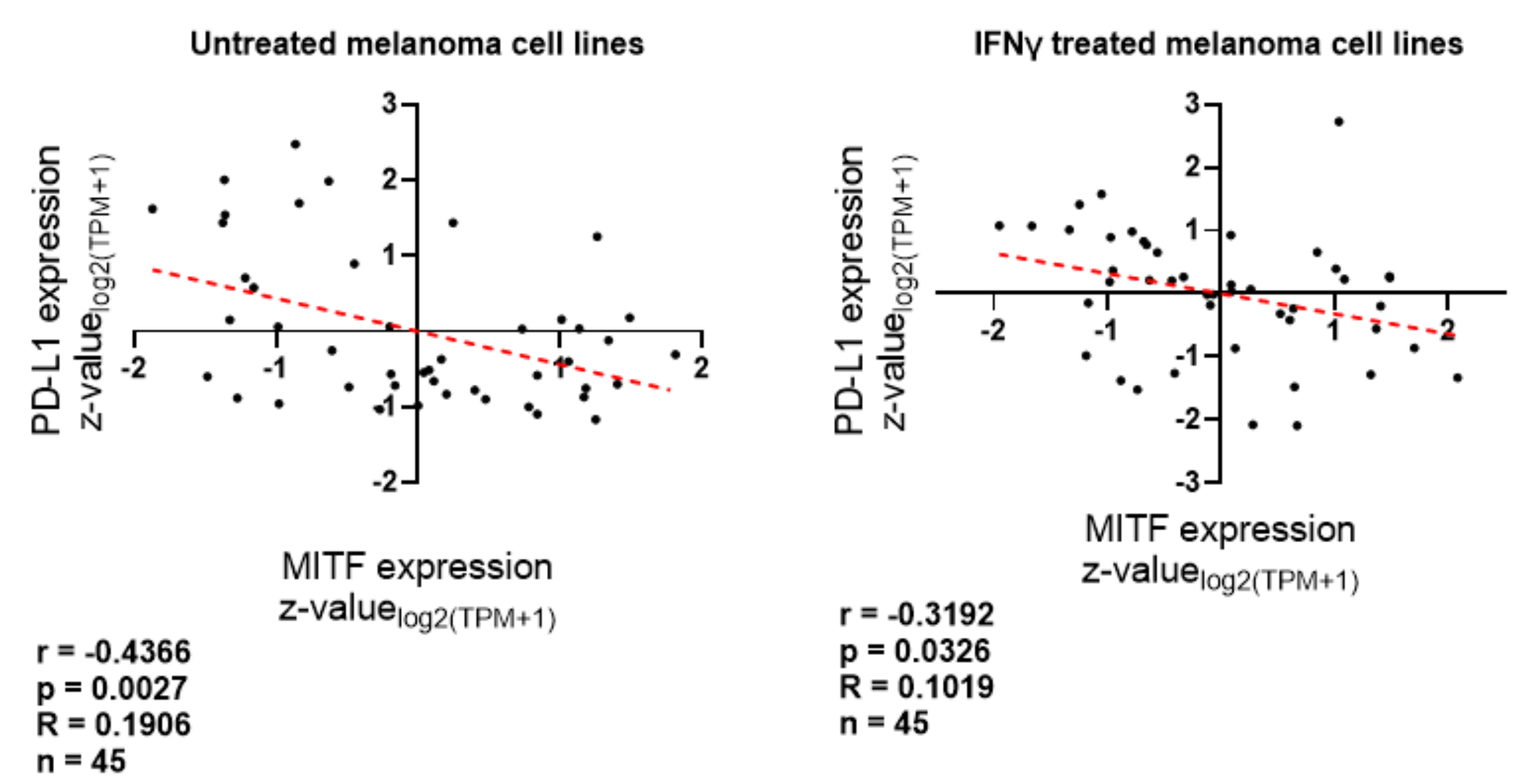

Supplement: Supplementary file 8 — Supplementary Material 8. Fig. S7 Simple linear regression analysis of the association between MITF expression and either basal PD-L1 expression (left) or IFNγ-induced PD-L1 expression in 45 patient derived melanoma cell lines form the GSE 154996 data set. [file 12964_2024_1963_MOESM8_ESM.png]

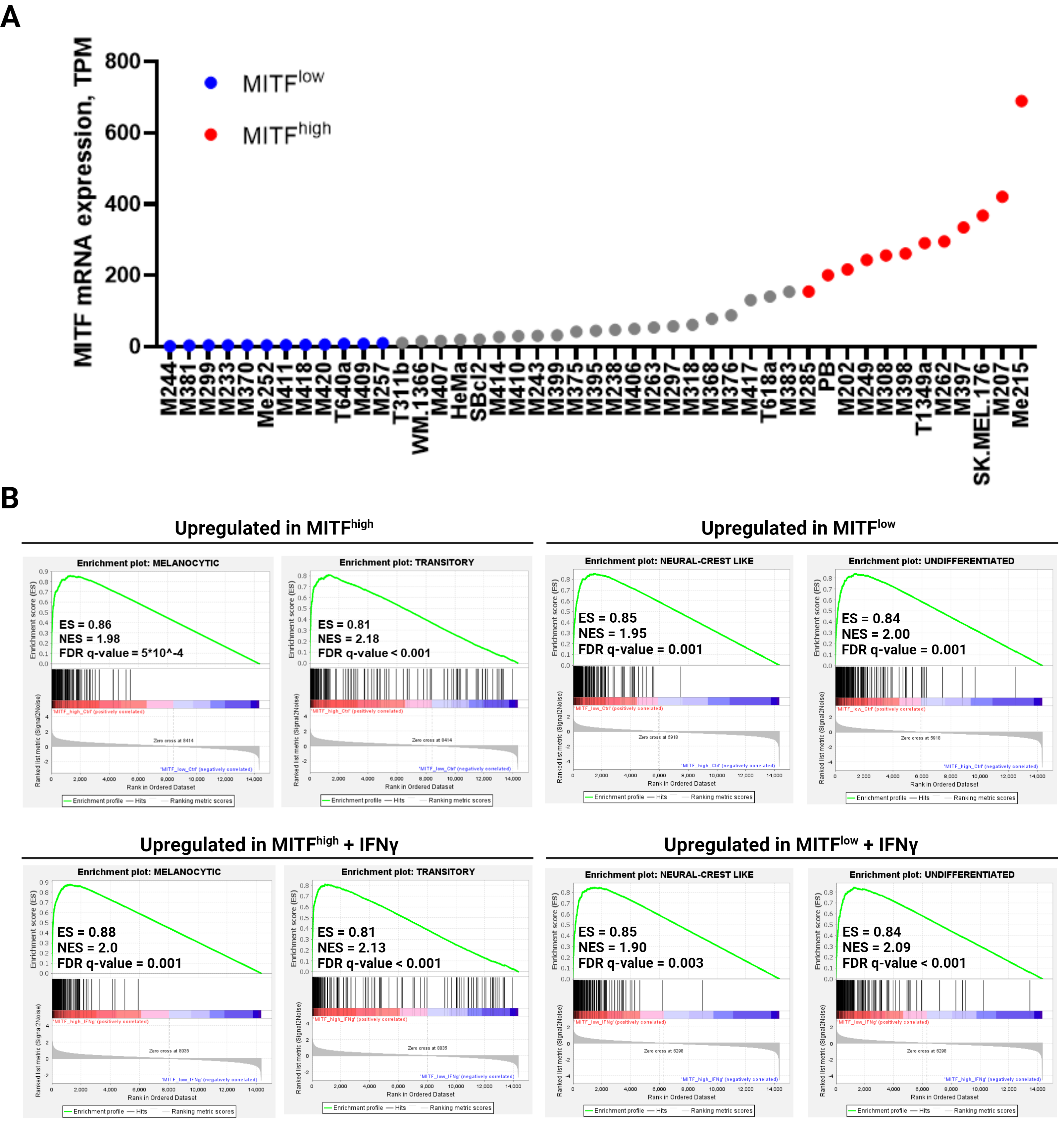

Supplement: Supplementary file 9 — Supplementary Material 9. Fig. S8 (A) MITF expression of the untreated wild-type melanoma cell lines from the GSE154996 data set, blue dots indicate the cell lines selected for the MITFlow group whereas red dots indicate the MITFhigh group. (B) GSEA comparing the MITFhigh and MITFlow groups, with and without IFNγ treatment, with regards to the Tsoi melanoma differentiation signature gene sets. [file 12964_2024_1963_MOESM9_ESM.png]

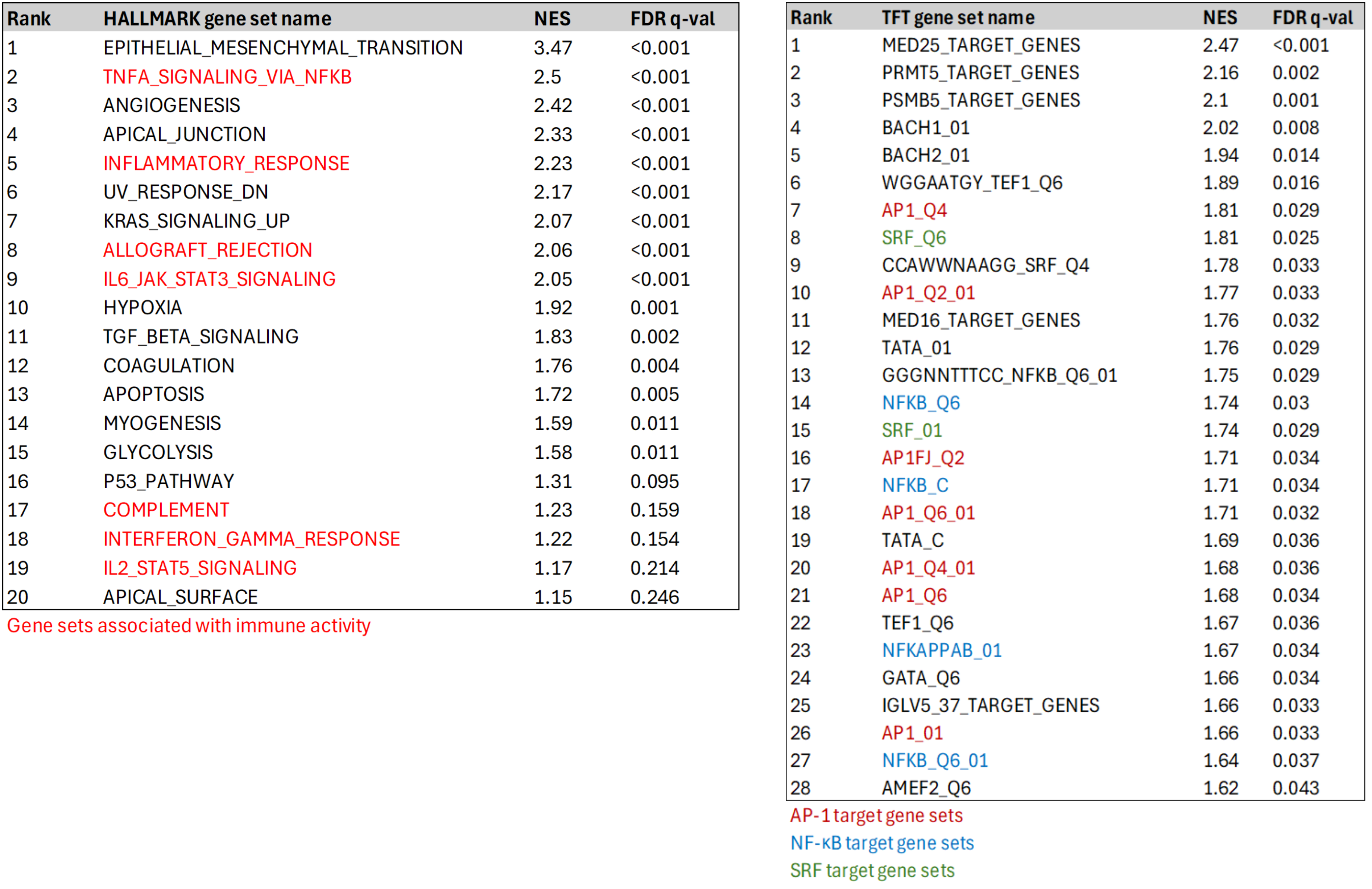

Supplement: Supplementary file 10 — Supplementary Material 10. Fig. S9 GSEA results showing the top 20 and top 28 upregulated hallmark (left) and transcription factor target gene (right) sets, respectively, in the IFNγ treated MITFlow group compared to the IFNγ treated MITFhigh group. Coloured TFT gene sets are associated with transcription factors previously linked to the MITFlow melanoma cell state. [file 12964_2024_1963_MOESM10_ESM.png]

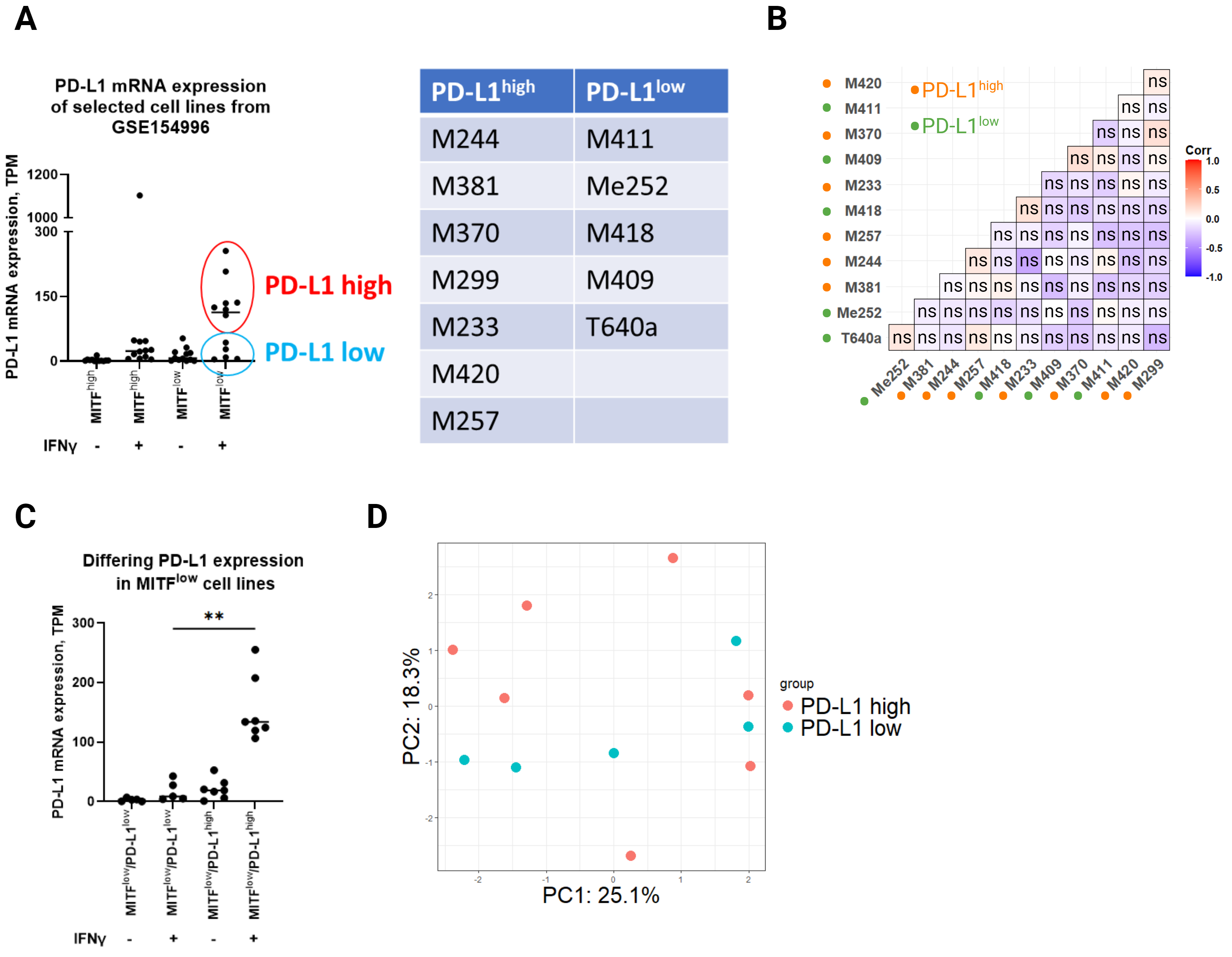

Supplement: Supplementary file 11 — Supplementary Material 11. Fig. S10 (A) Graph and table showing the categorization of MITFlow melanoma cells into either PD-L1high or PD-L1low. (B) Pearson correlation matrix comparing the selected IFNγ treated MITFlow melanoma cell lines based on overall mRNA expression, adjusting for multiple testing with Bonferroni correction. (C) PD-L1 expression in the different PD-L1 groups of MITFlow cells, statistical analysis performed by Statistical analysis performed by Mann-Whitney U test, P value ** =< 0.01 (n = 5 for PD-L1low, n = 7 for PD-L1high). (D) Principal component analysis (same analysis as presented in figure 10B) comparing MITFlow cells based on their PD-L1 expression, showing no apparent segregation or clustering of the two groups along PC1 and PC2. [file 12964_2024_1963_MOESM11_ESM.png]

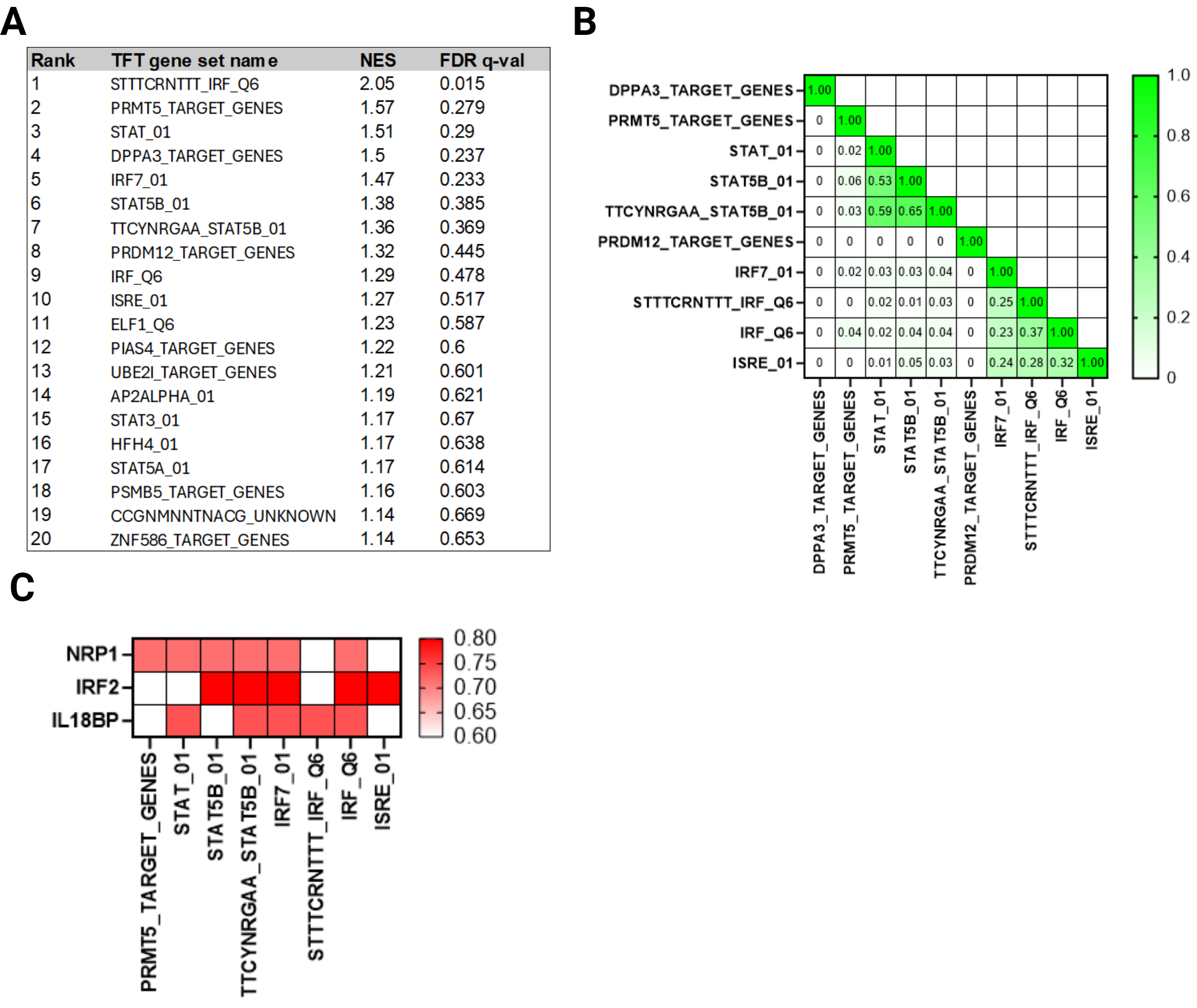

Supplement: Supplementary file 12 — Supplementary Material 12. Fig. S11 (A) Top 20 upregulated gene transcription factor target gene sets in the PD-L1high group when compared to the PD-L1low group. (B) Overlap of the top leading-edge subsets of the top ten gene sets presented in (A). (C) Most commonly shared genes among the leading-edge subsets in question, colour gradient depicts a genes contribution to the respective subset. [file 12964_2024_1963_MOESM12_ESM.png]

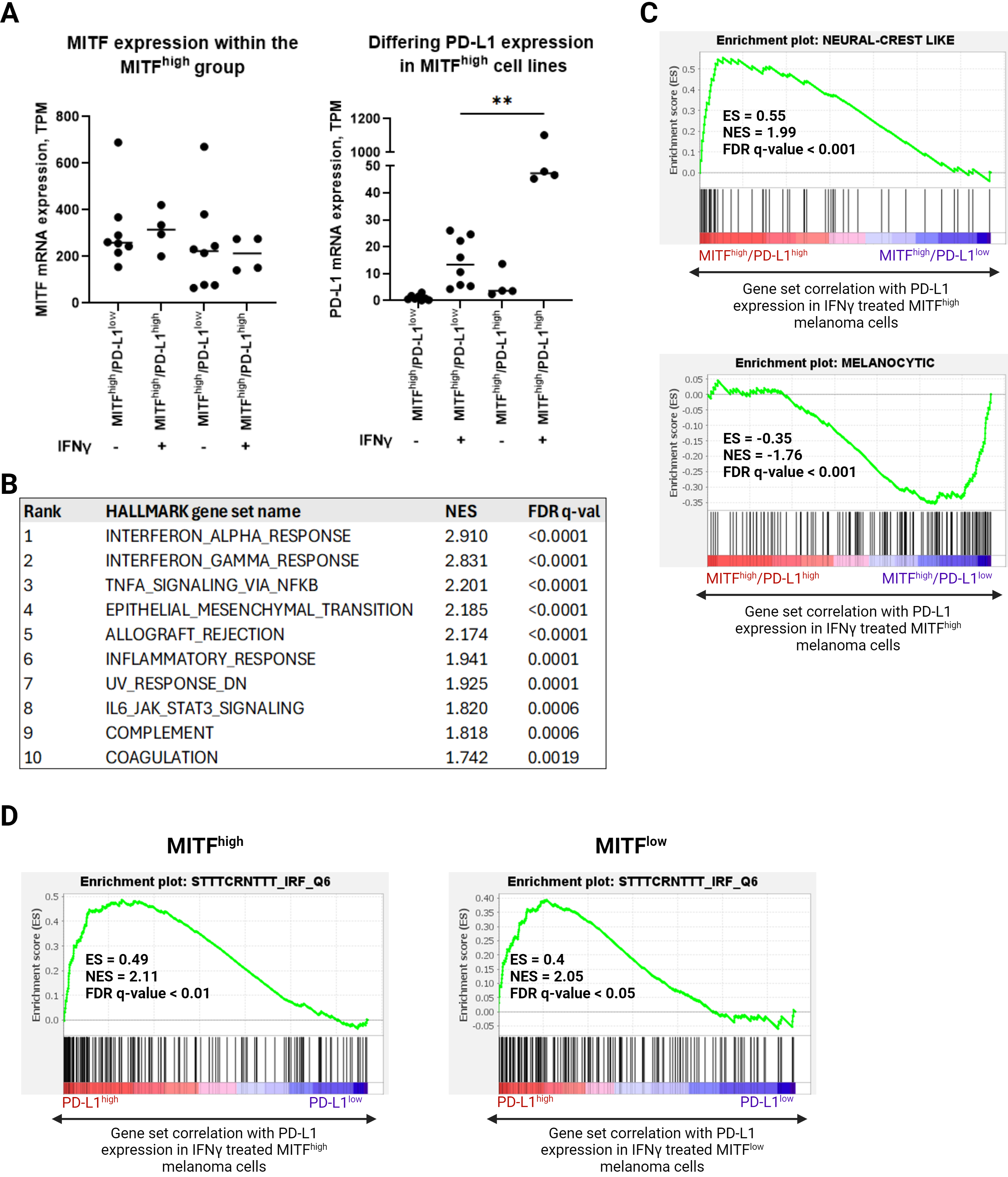

Supplement: Supplementary file 13 — Supplementary Material 13. Fig. S12 (A) MITF (left) and PD-L1 expression (right) among the MITFhigh melanoma cell group after categorizing by PD-L1 expression, statistical analysis performed by Mann-Whitney U test, P value ** = < 0.01 (n = 8 for PD-L1low, n = 4 for PD-L1high). (B) Top 10 hallmark gene sets upregulated in the MITFhigh/PD-L1high cells compared to the MITFhigh/PD-L1low cells. (C) Tsoi differentiation signature gene set enrichment in the MITFhigh/PD-L1high cells compared to the MITFhigh/PD-L1low cells. (D) Enrichment plots of the “STTTCRNTTT_IRF_Q6” TFT gene set regulation in MITFhigh/PD-L1high cells compared to the MITFhigh/PD-L1low cells (left) and in MITFlow/PD-L1high cells compared to the MITFlow/PD-L1low cells (right). [file 12964_2024_1963_MOESM13_ESM.png]

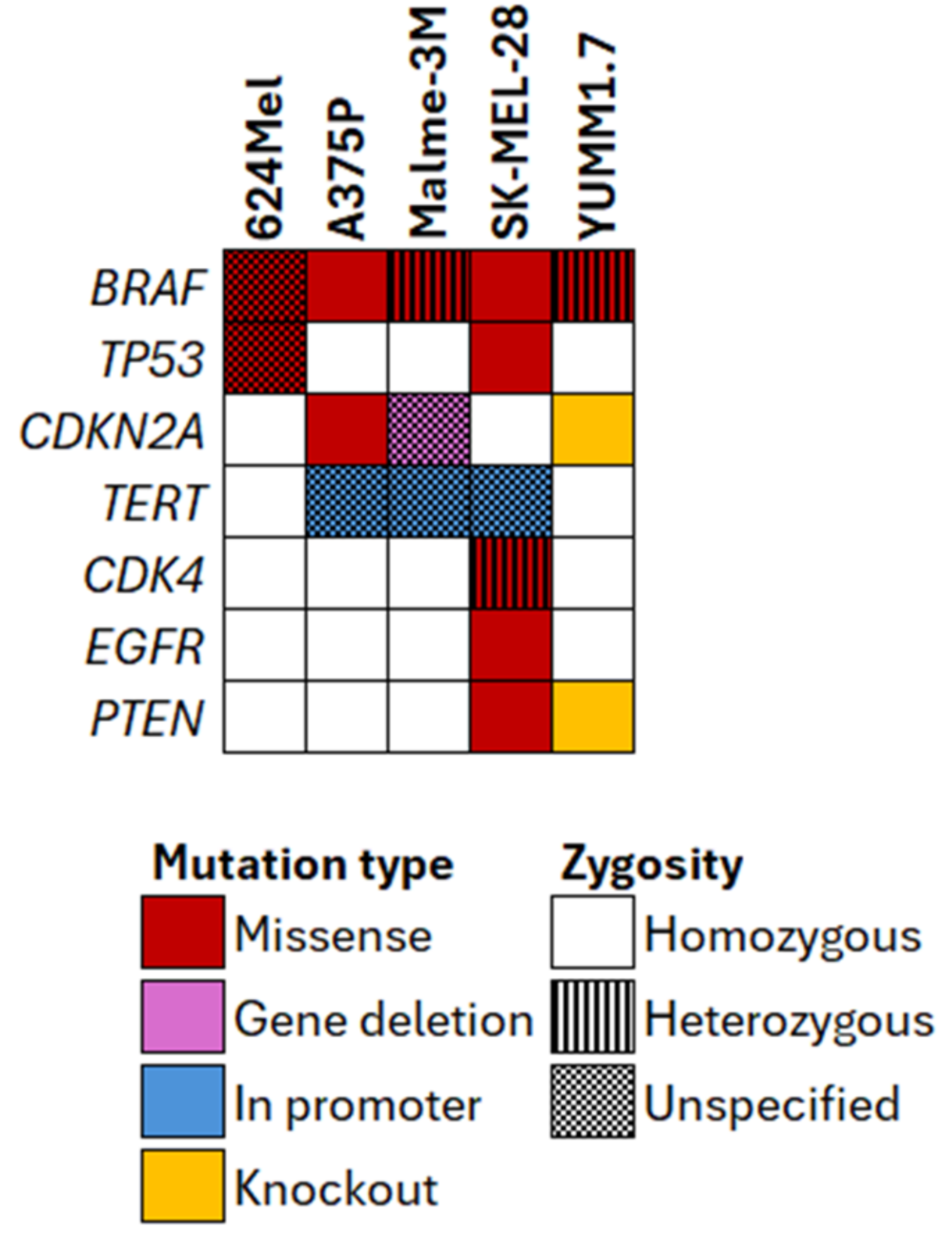

Supplement: Supplementary file 14 — Supplementary Material 14. Fig. S13 Mutational status of the melanoma cell lines used in this study, according to the Cellosaurus database. The mouse melanoma cell line B16 has no sequence variations listed in the database. [file 12964_2024_1963_MOESM14_ESM.png]

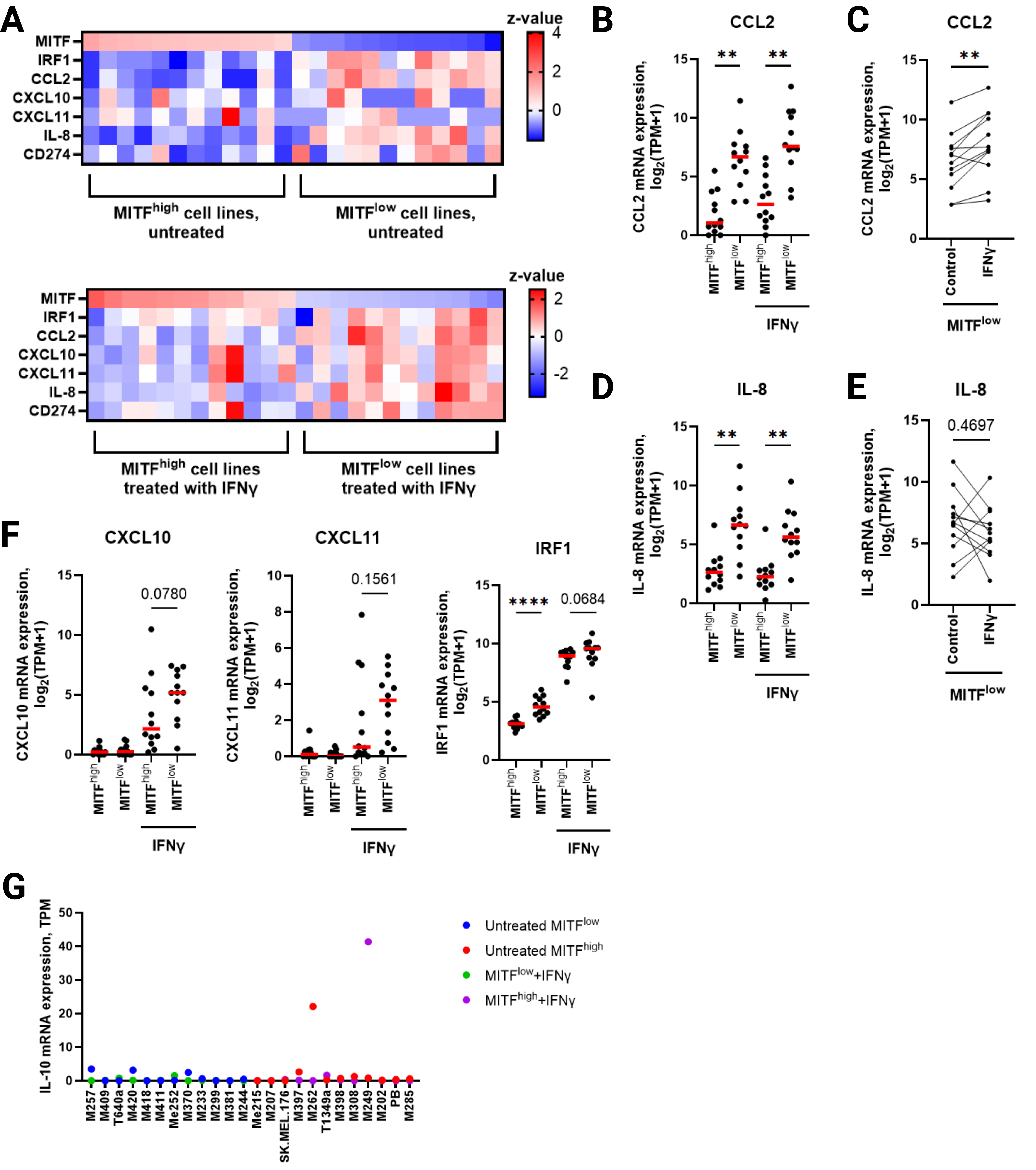

Supplement: Supplementary file 15 — Supplementary Material 15. Fig. S14 Comparison of IRF1,CCL2, CXCL10, CXCL11, IL-8 and CD274 gene expression across the MITFhigh and MITFlow melanoma cell lines from the GSE15449 dataset. (A) Heatmaps depicting overall gene expression across the two groups, either untreated (upper heatmap) or with IFNγ treatment (lower heatmap). (B) CCL2 mRNA expression across all conditions. (C) Paired comparison of CCL2 mRNA expression in the MITFlow melanoma cell lines. (D) IL-8 mRNA expression across all conditions. (E) Paired comparison of CCL2 mRNA expression in the MITFlow melanoma cell lines. (F) Comparison of mRNA expression of CXCL10, CXCL11 and IRF1 across all conditions. (G) IL-10 mRNA expression in every individual cell line belonging to either the MITFhigh or MITFlow group, with or without IFNγ treatment. Lines in scatterplots represent medians. Statistical analysis performed by Kruskal-Wallis and Dunn’s multiple comparisons test (B & E), two-tailed paired Wilcoxon test (C & E) or Mann-Whitney U test (F), adjusted P value ** = < 0.01, **** = < 0.0001 (n = 12). [file 12964_2024_1963_MOESM15_ESM.png]
