# Supplementary material for: Differentiation status determines the effects of IFNγ on the expression of PD-L1 and immunomodulatory genes in melanoma
Source: Cell Commun Signal. 2024 Dec 31;22:618. doi: 10.1186/s12964-024-01963-6 (PMC11687009; doi:10.1186/s12964-024-01963-6)
Supplement: Supplementary file 16 — Supplementary Material 16. Uncropped WB figures. [file 12964_2024_1963_MOESM16_ESM.pptx]

## Slide 1
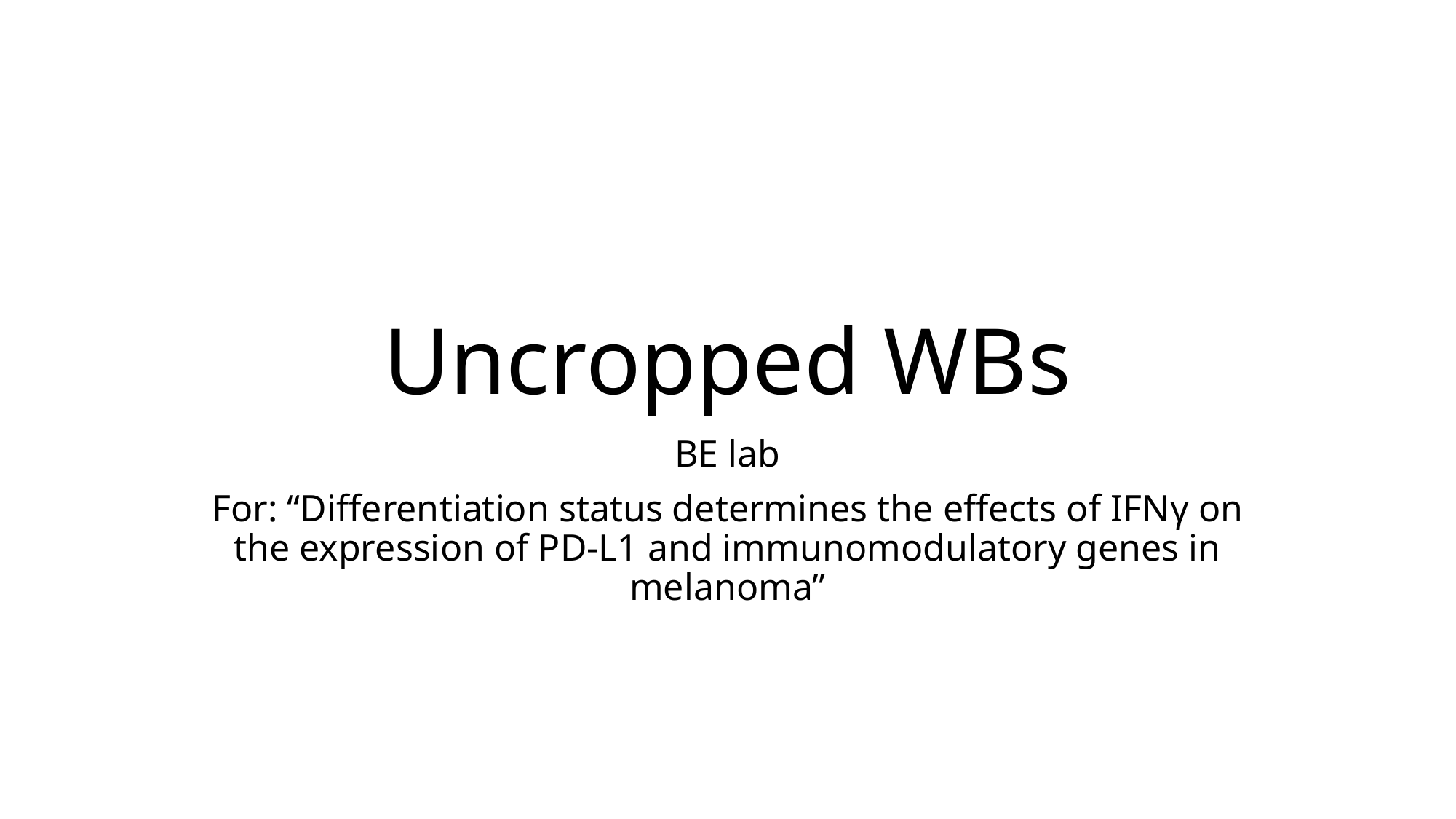

# Uncropped WBs
BE lab
For: “Differentiation status determines the effects of IFNγ on the expression of PD-L1 and immunomodulatory genes in melanoma”

## Slide 2
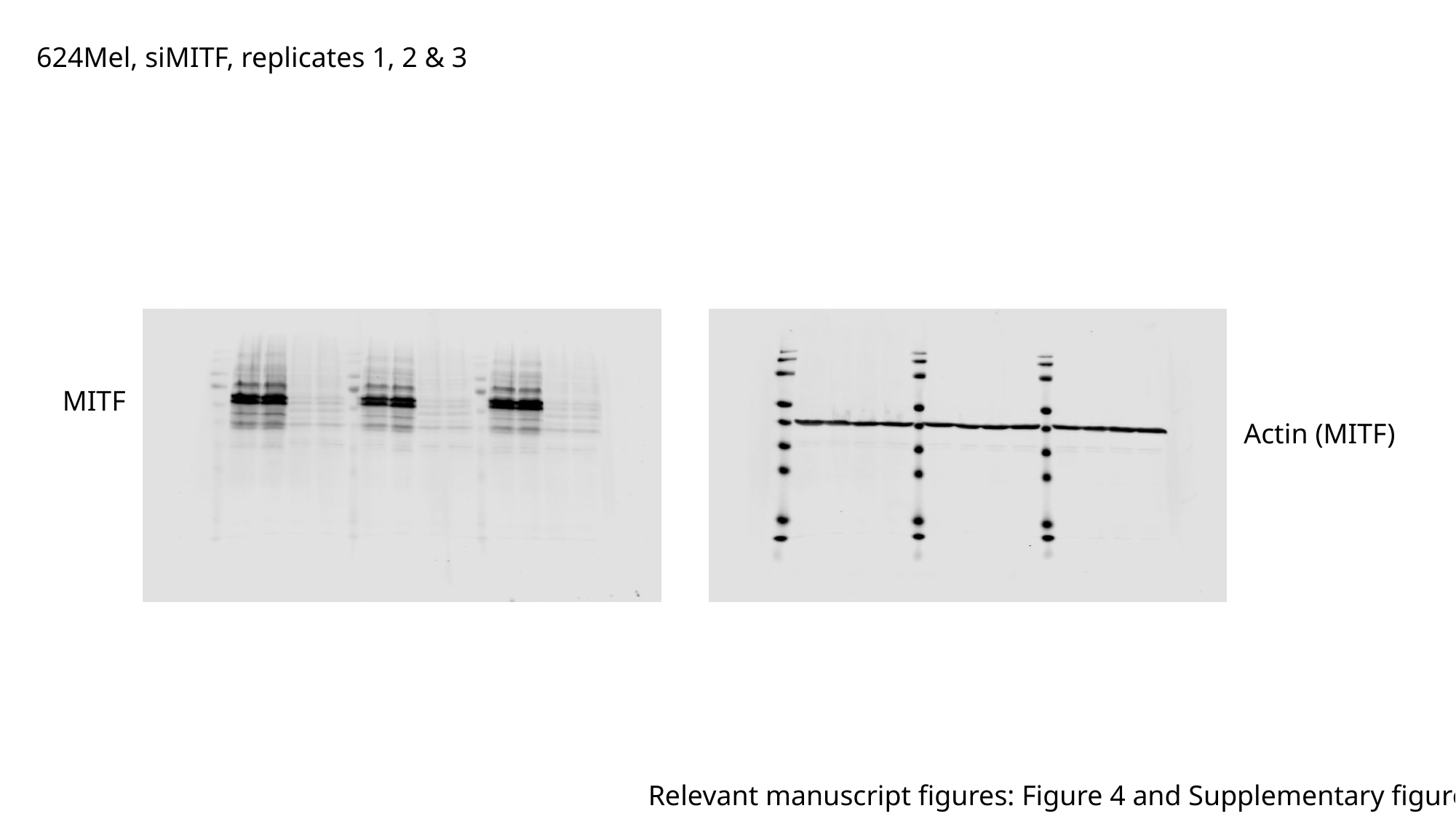

624Mel, siMITF, replicates 1, 2 & 3
MITF
Actin (MITF)
Relevant manuscript figures: Figure 4 and Supplementary figure 2

## Slide 3
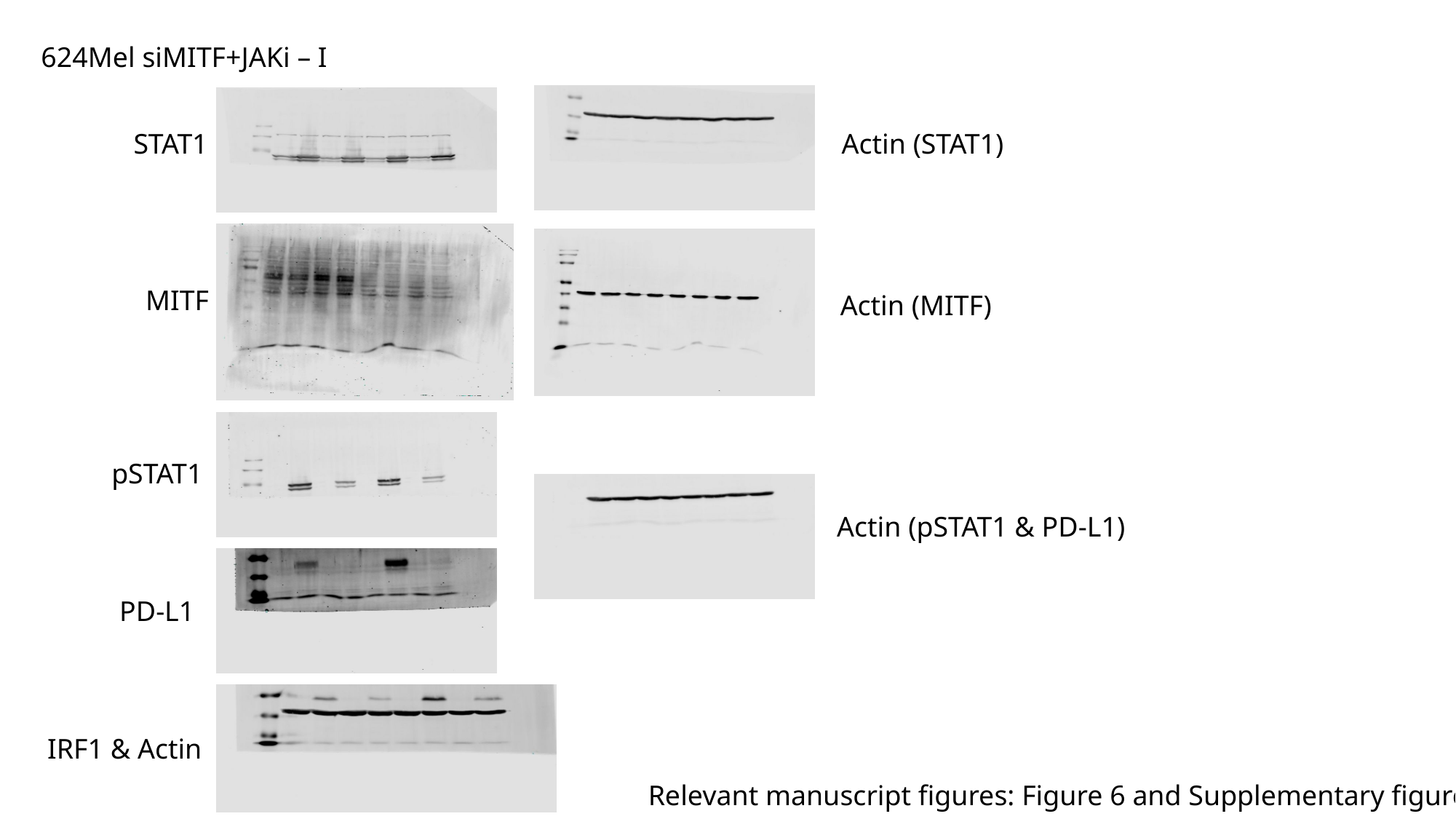

624Mel siMITF+JAKi – I
STAT1
Actin (STAT1)
MITF
Actin (MITF)
pSTAT1
Actin (pSTAT1 & PD-L1)
PD-L1
IRF1 & Actin
Relevant manuscript figures: Figure 6 and Supplementary figure 5

## Slide 4
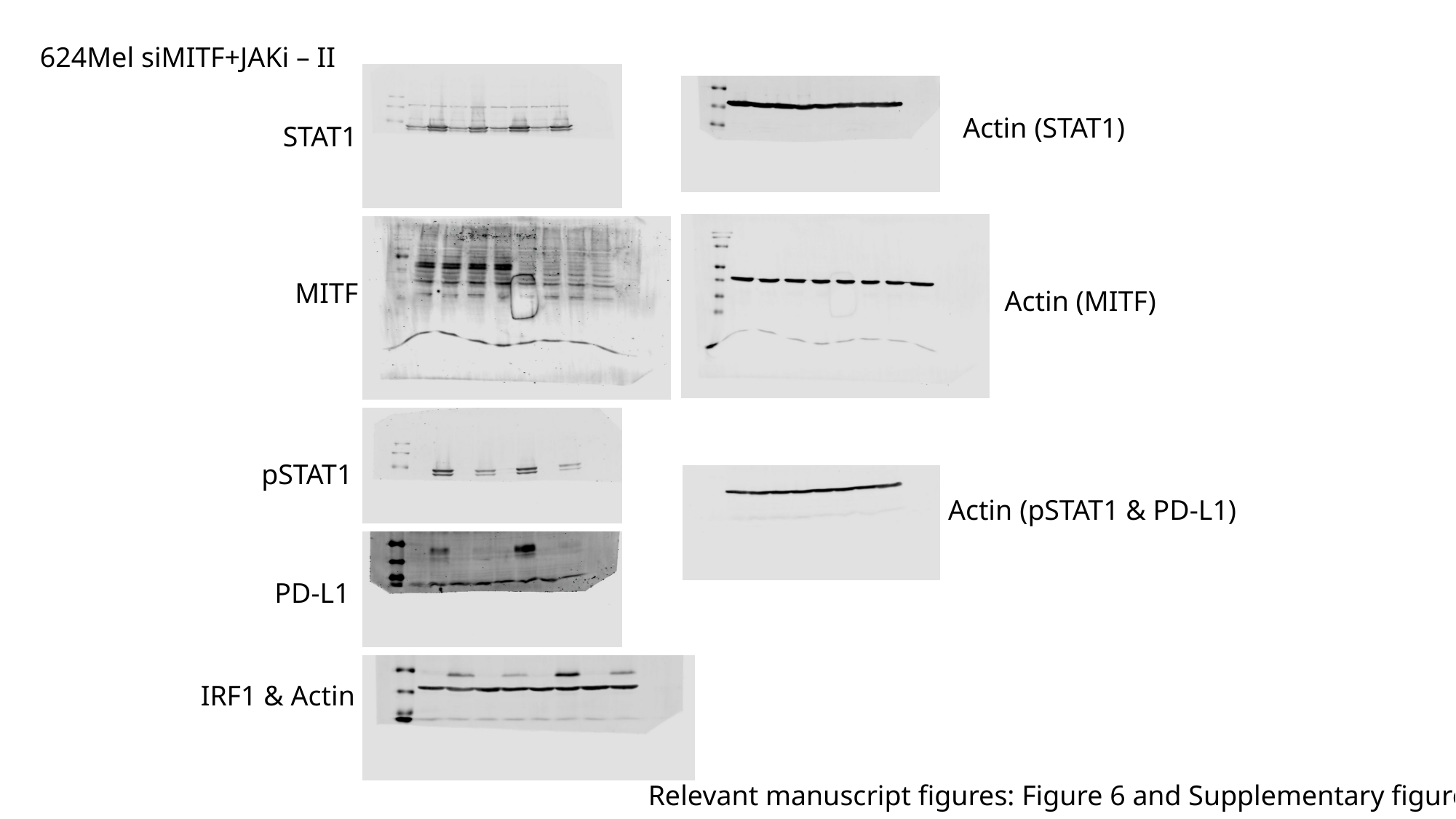

624Mel siMITF+JAKi – II
Actin (STAT1)
STAT1
MITF
Actin (MITF)
pSTAT1
Actin (pSTAT1 & PD-L1)
PD-L1
IRF1 & Actin
Relevant manuscript figures: Figure 6 and Supplementary figure 5

## Slide 5
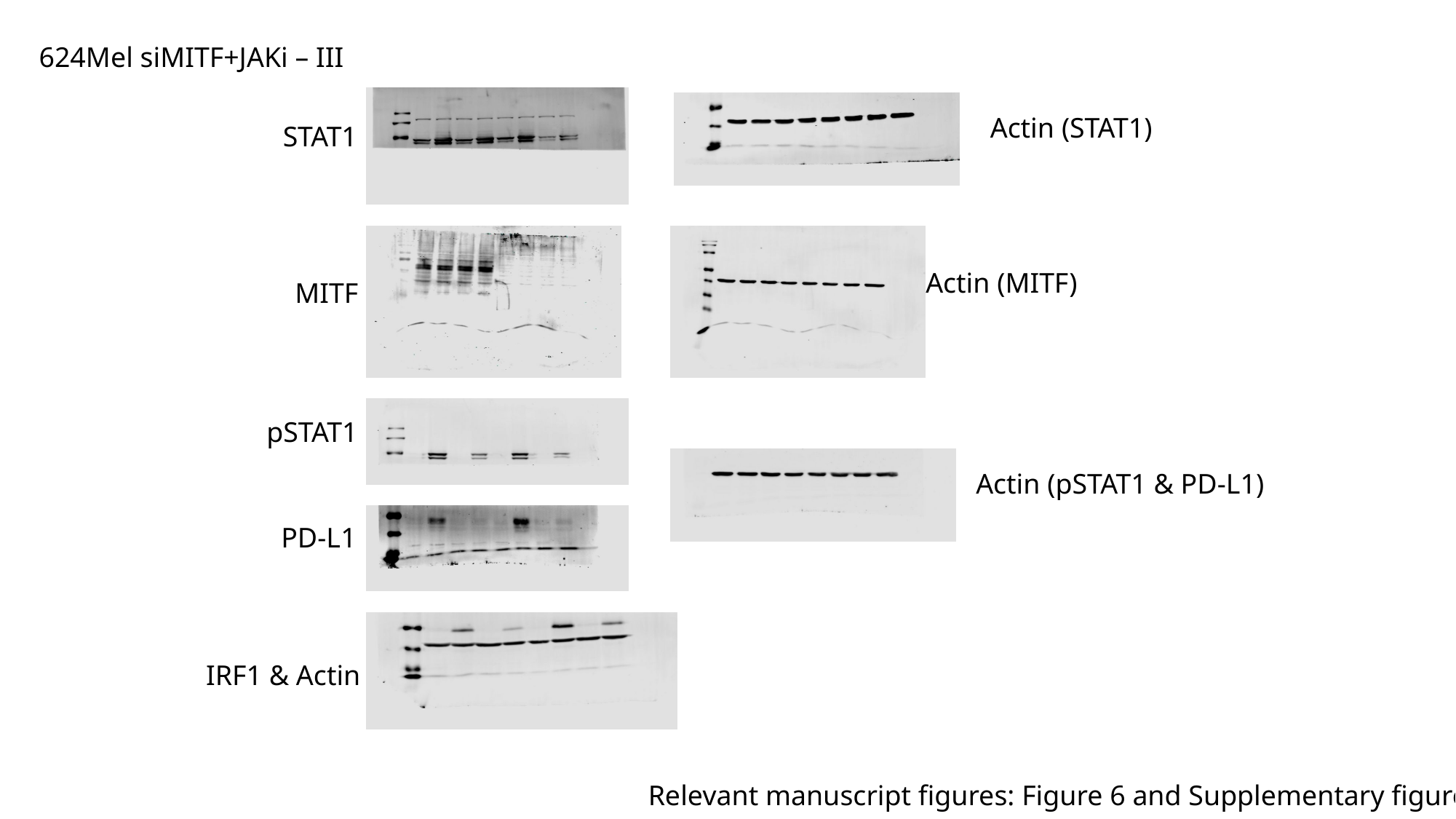

624Mel siMITF+JAKi – III
Actin (STAT1)
STAT1
Actin (MITF)
MITF
pSTAT1
Actin (pSTAT1 & PD-L1)
PD-L1
IRF1 & Actin
Relevant manuscript figures: Figure 6 and Supplementary figure 5

## Slide 6
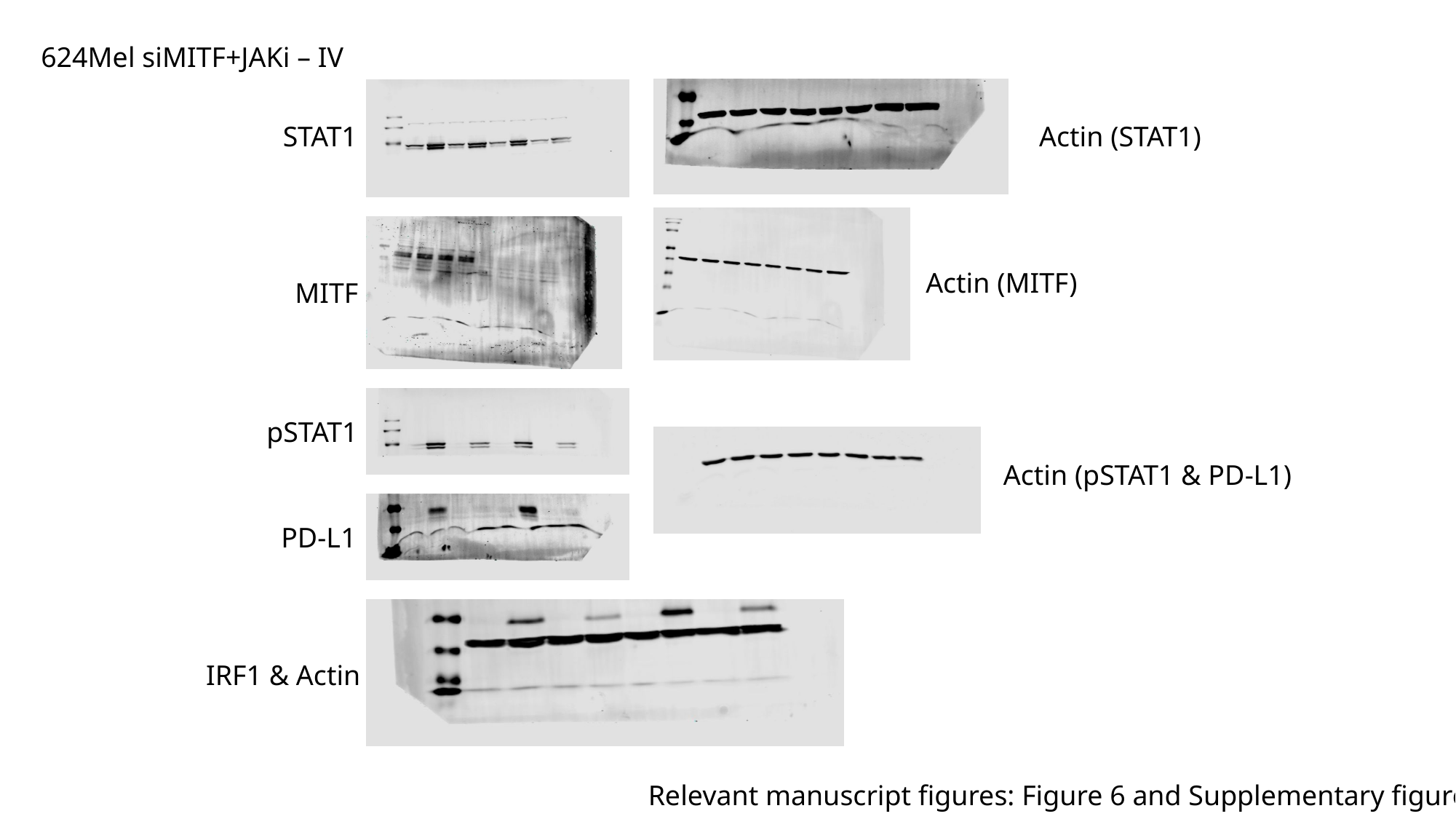

624Mel siMITF+JAKi – IV
STAT1
Actin (STAT1)
Actin (MITF)
MITF
pSTAT1
Actin (pSTAT1 & PD-L1)
PD-L1
IRF1 & Actin
Relevant manuscript figures: Figure 6 and Supplementary figure 5

## Slide 7
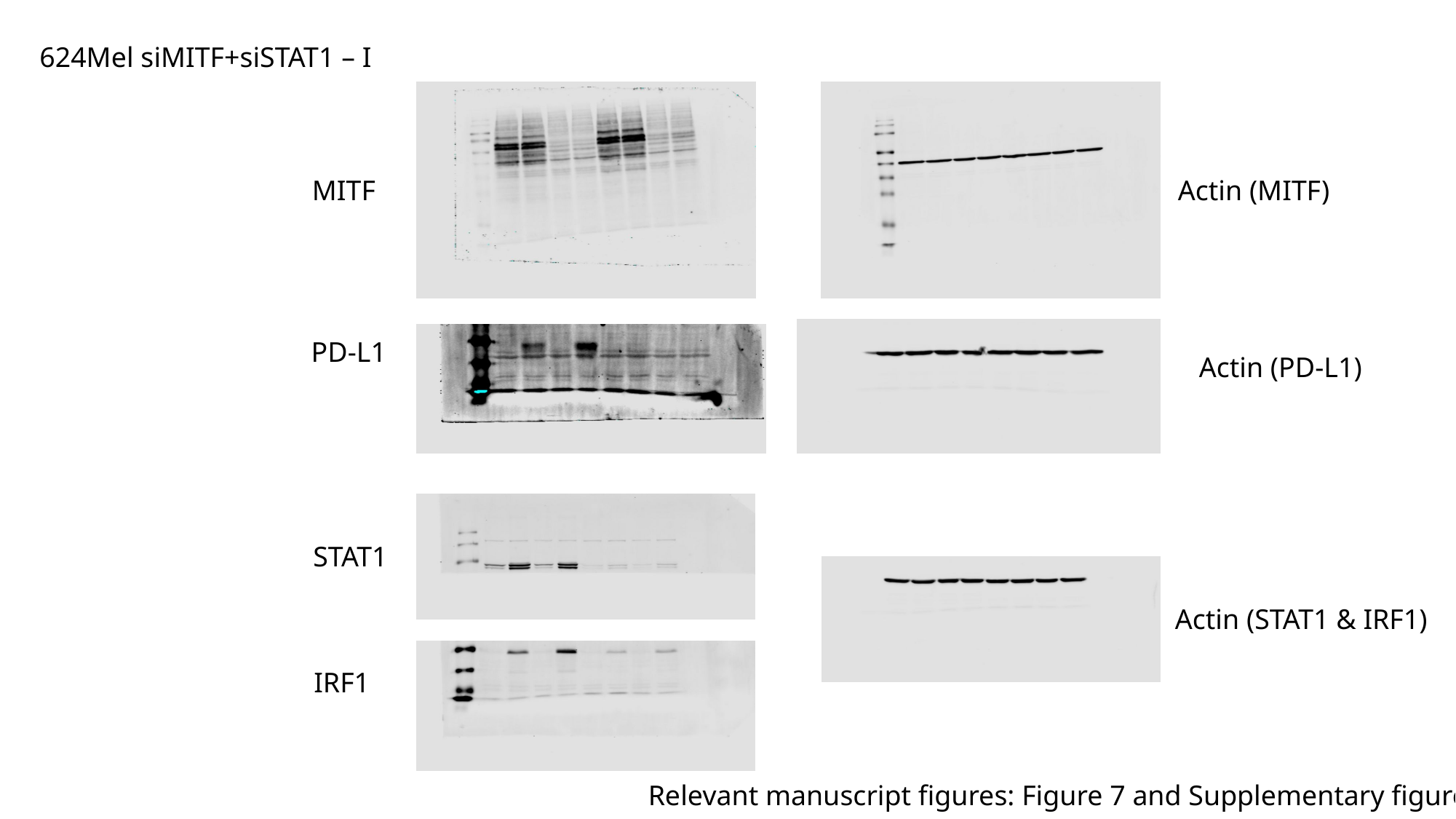

624Mel siMITF+siSTAT1 – I
MITF
Actin (MITF)
PD-L1
Actin (PD-L1)
STAT1
Actin (STAT1 & IRF1)
IRF1
Relevant manuscript figures: Figure 7 and Supplementary figure 5

## Slide 8
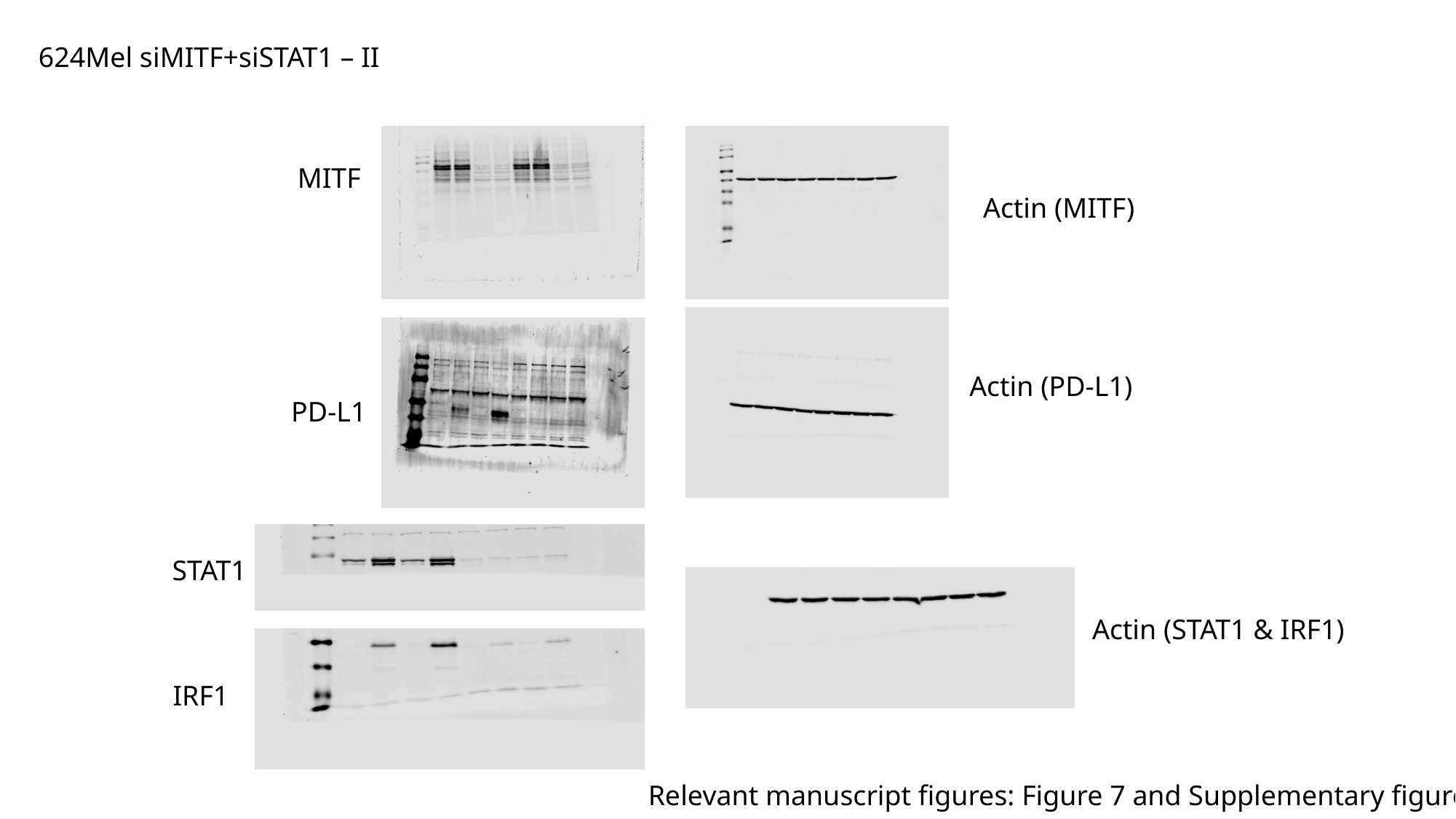

624Mel siMITF+siSTAT1 – II
MITF
Actin (MITF)
Actin (PD-L1)
PD-L1
STAT1
Actin (STAT1 & IRF1)
IRF1
Relevant manuscript figures: Figure 7 and Supplementary figure 5

## Slide 9
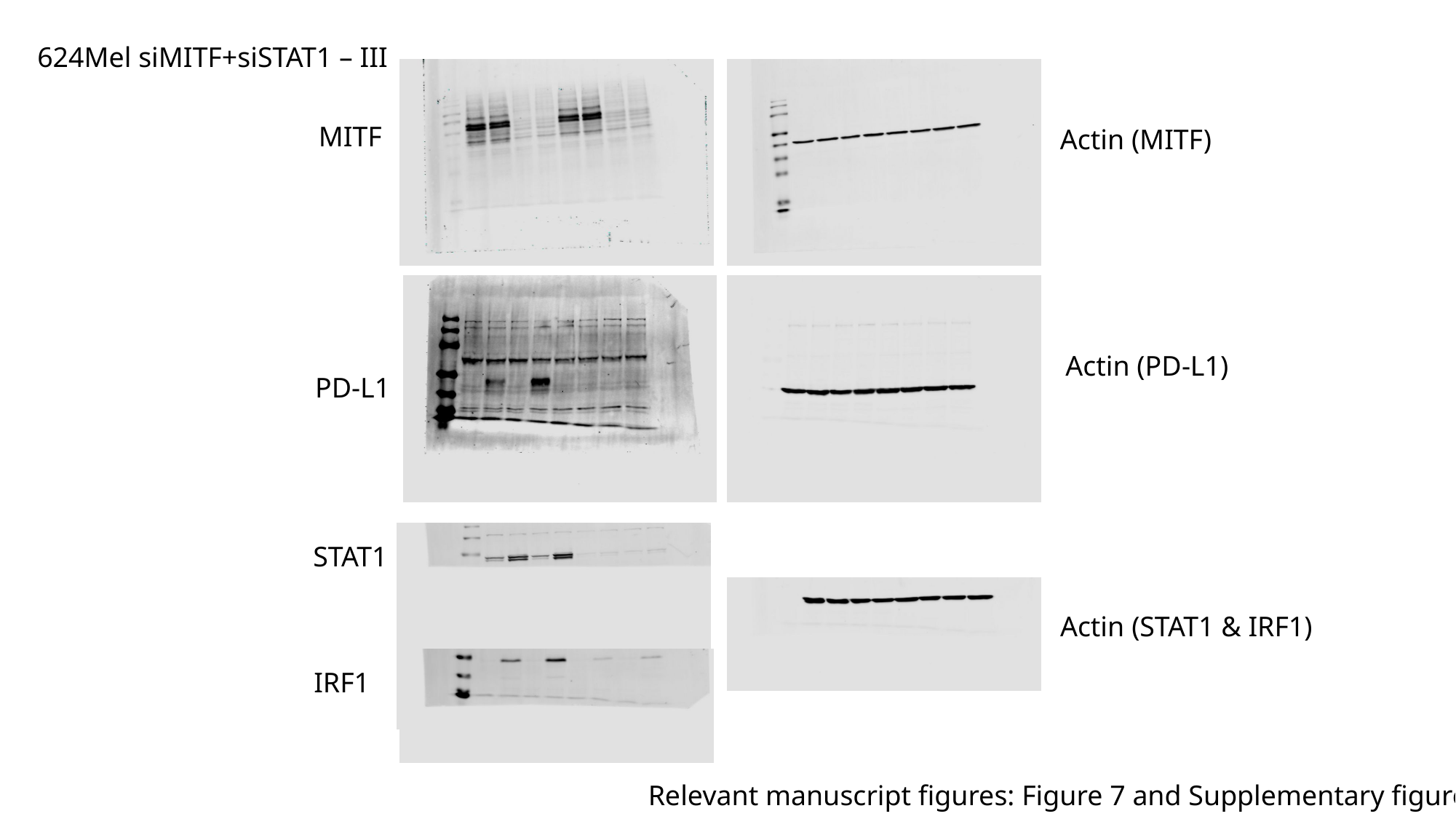

624Mel siMITF+siSTAT1 – III
MITF
Actin (MITF)
Actin (PD-L1)
PD-L1
STAT1
Actin (STAT1 & IRF1)
IRF1
Relevant manuscript figures: Figure 7 and Supplementary figure 5

## Slide 10
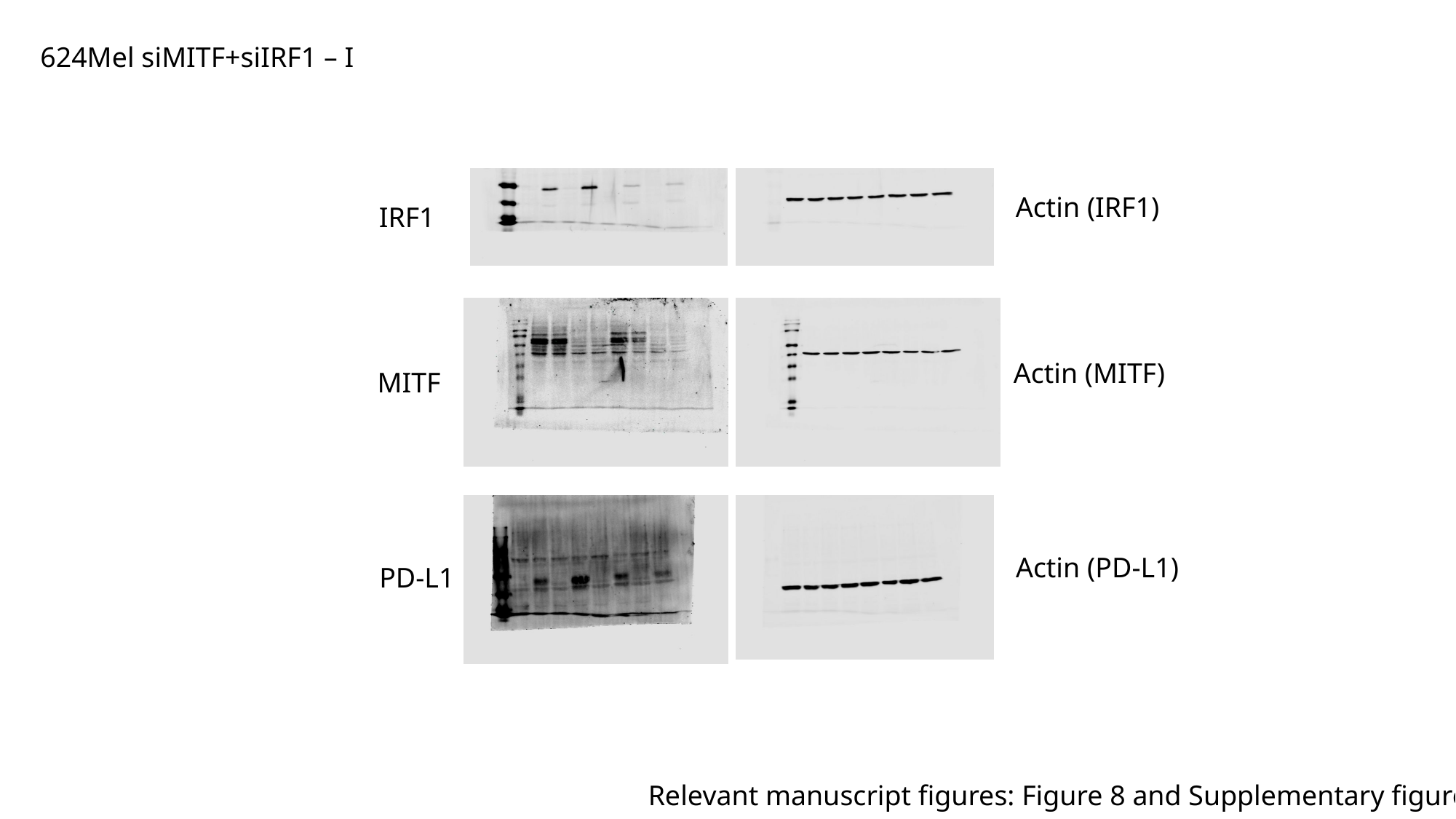

624Mel siMITF+siIRF1 – I
Actin (IRF1)
IRF1
Actin (MITF)
MITF
Actin (PD-L1)
PD-L1
Relevant manuscript figures: Figure 8 and Supplementary figure 5

## Slide 11
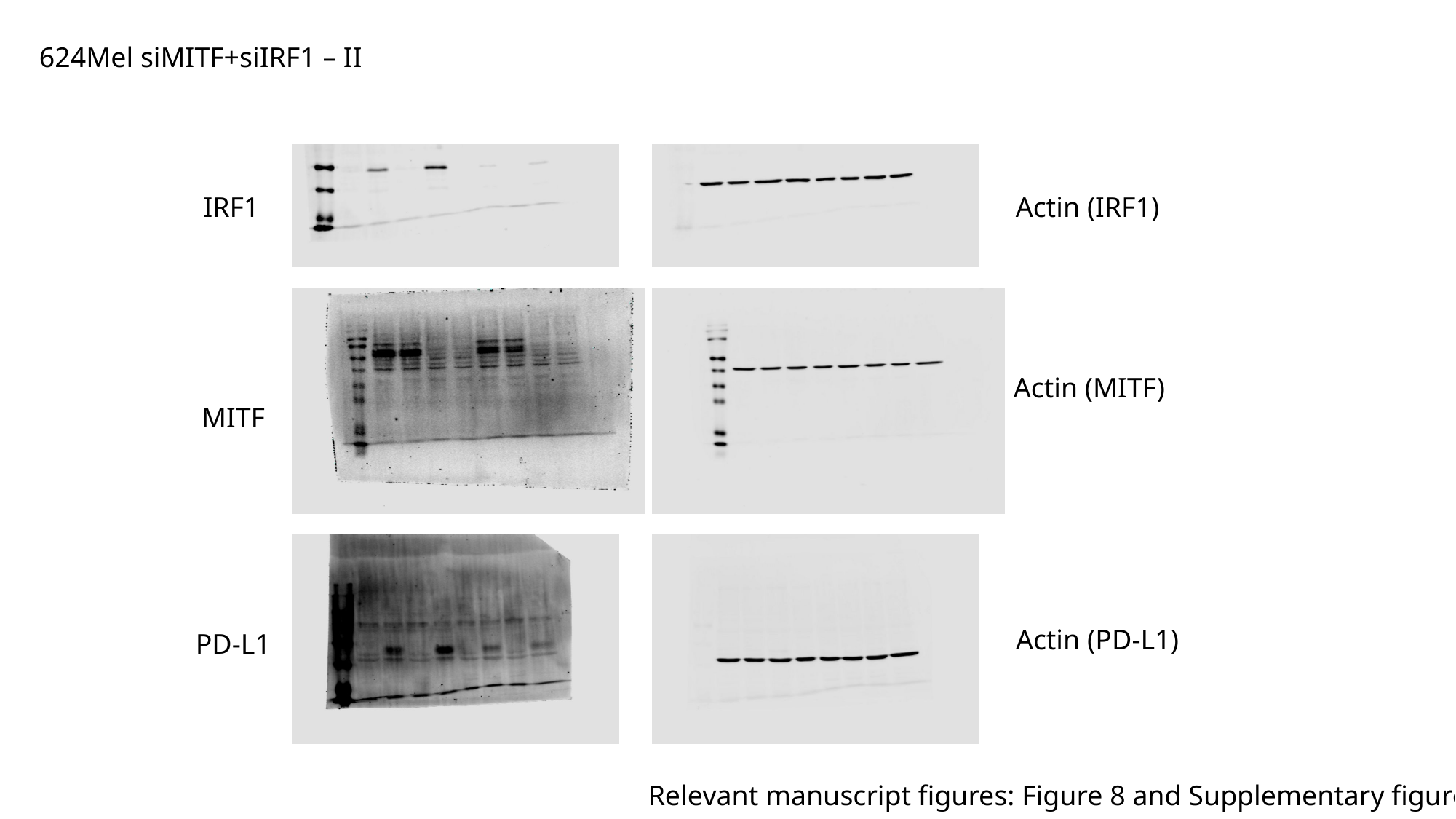

624Mel siMITF+siIRF1 – II
IRF1
Actin (IRF1)
Actin (MITF)
MITF
Actin (PD-L1)
PD-L1
Relevant manuscript figures: Figure 8 and Supplementary figure 5

## Slide 12
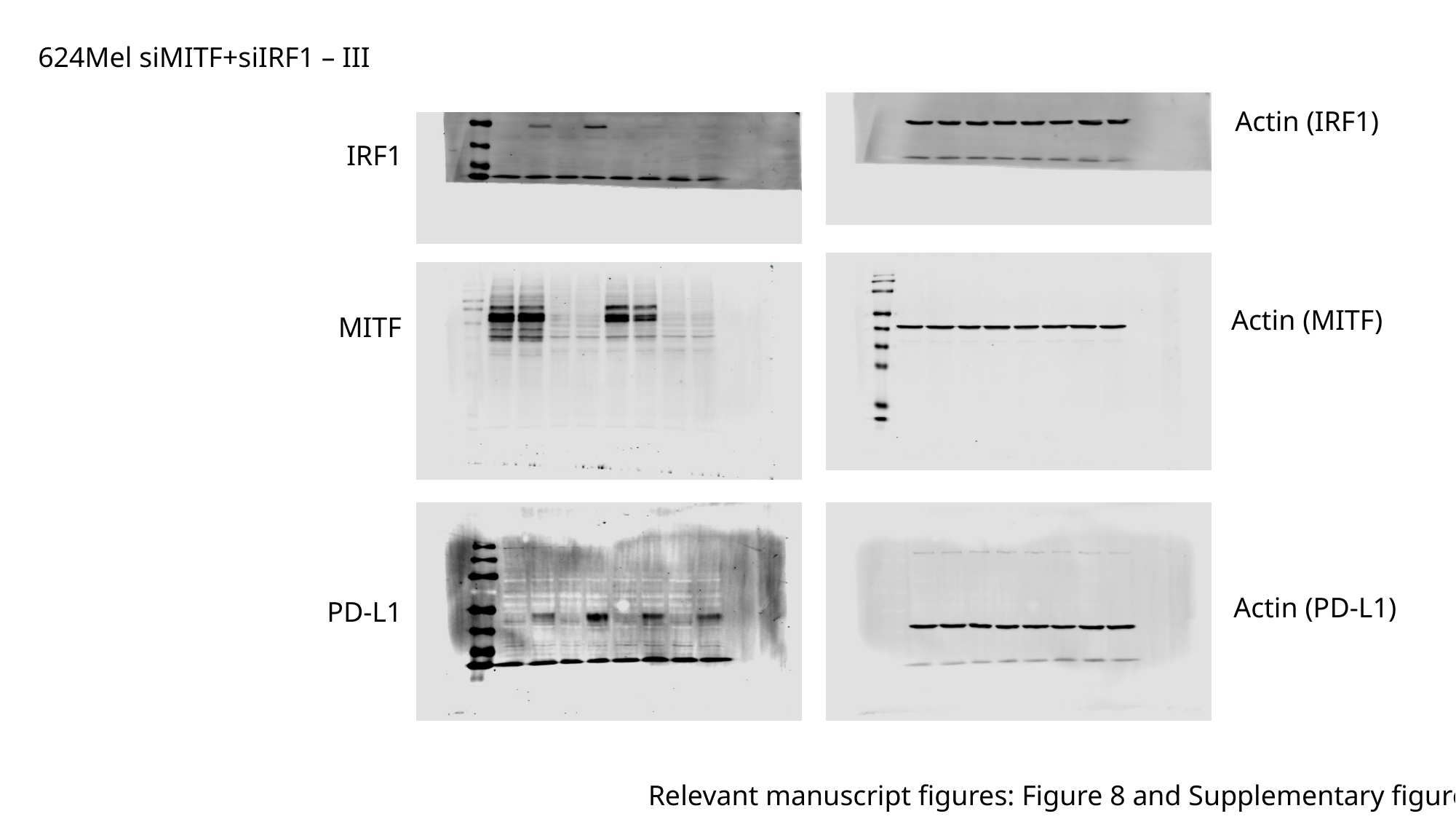

624Mel siMITF+siIRF1 – III
Actin (IRF1)
IRF1
Actin (MITF)
MITF
Actin (PD-L1)
PD-L1
Relevant manuscript figures: Figure 8 and Supplementary figure 5

## Slide 13
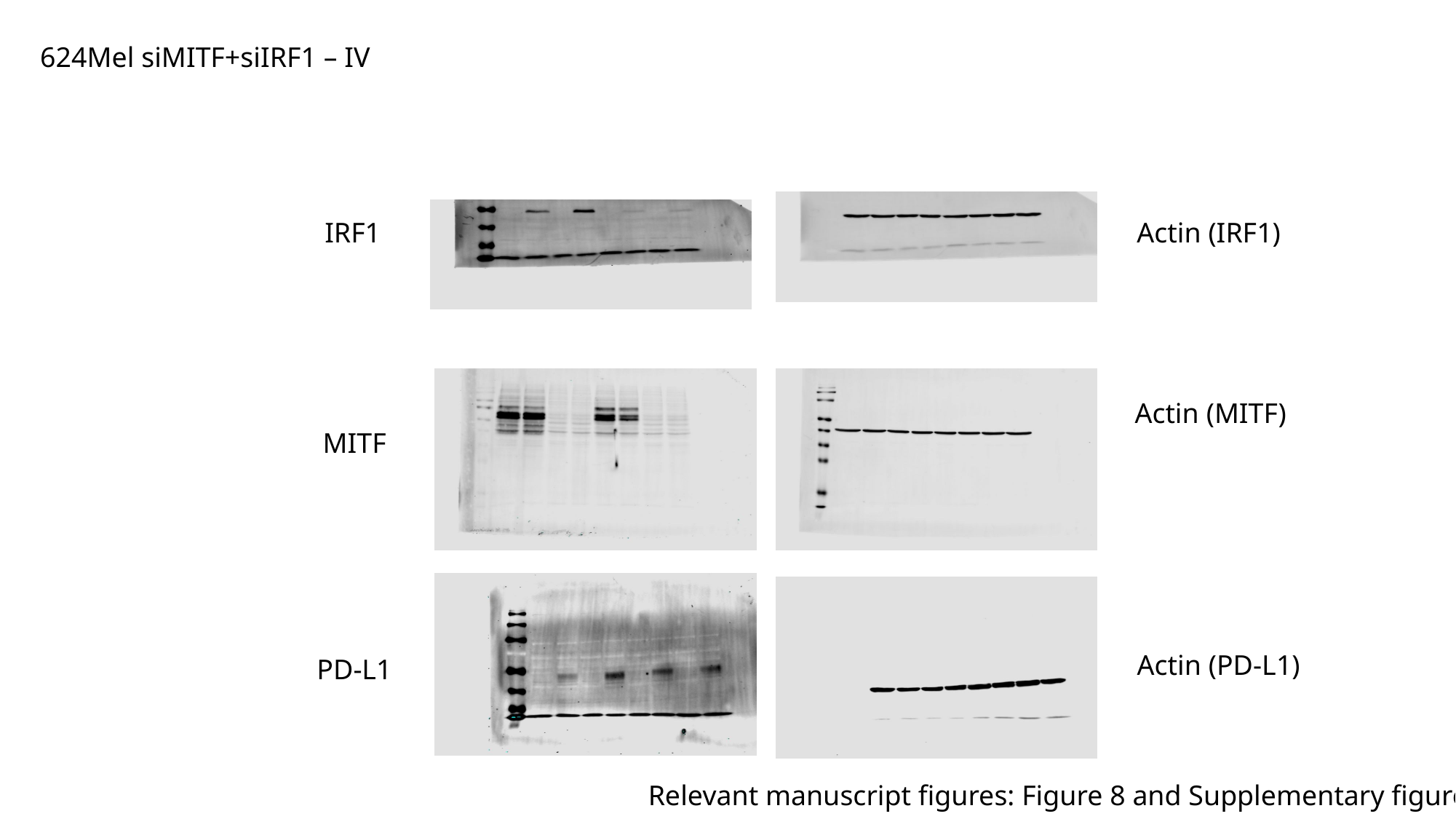

624Mel siMITF+siIRF1 – IV
IRF1
Actin (IRF1)
Actin (MITF)
MITF
Actin (PD-L1)
PD-L1
Relevant manuscript figures: Figure 8 and Supplementary figure 5

## Slide 14
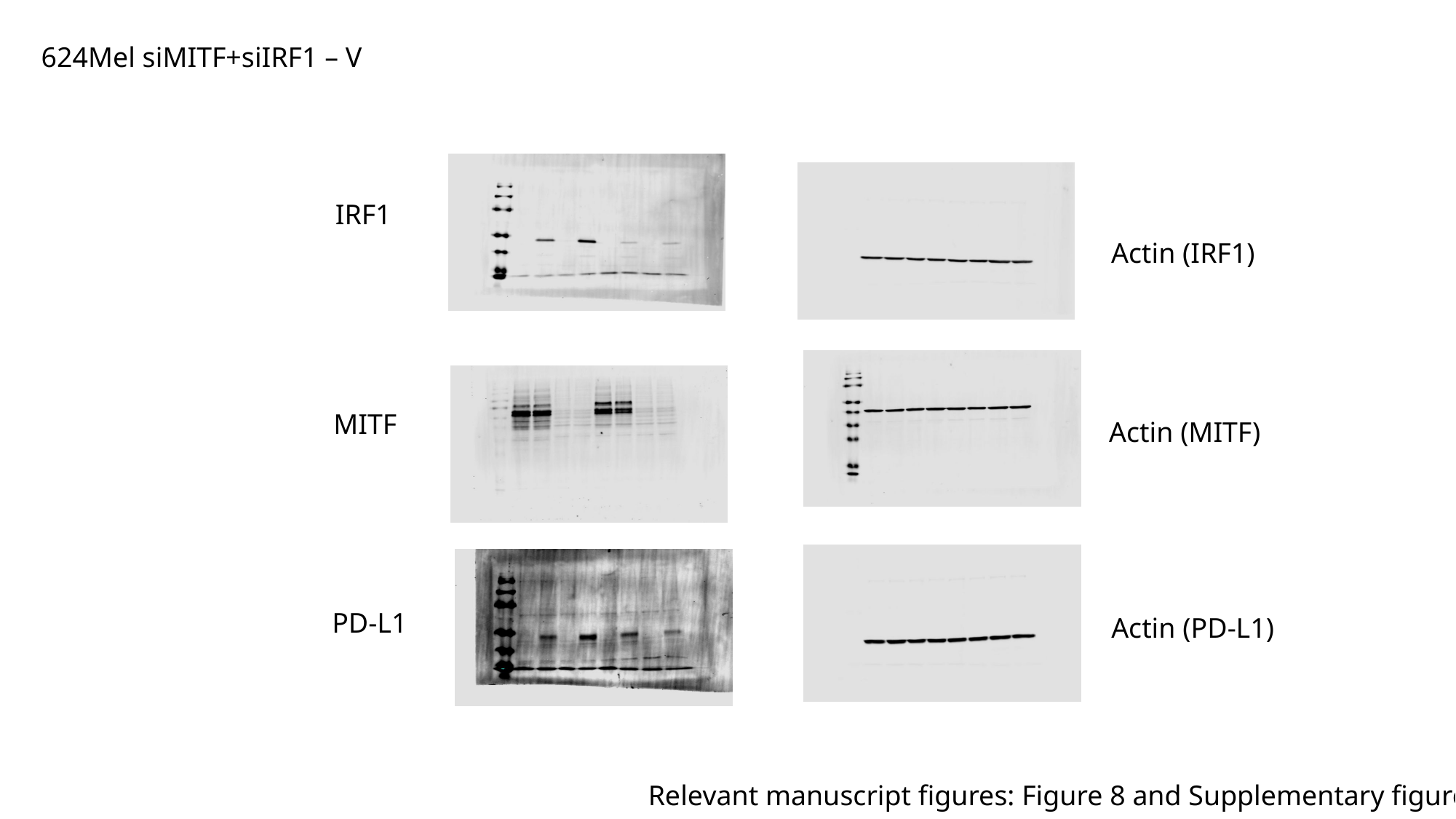

624Mel siMITF+siIRF1 – V
IRF1
Actin (IRF1)
MITF
Actin (MITF)
PD-L1
Actin (PD-L1)
Relevant manuscript figures: Figure 8 and Supplementary figure 5

## Slide 15
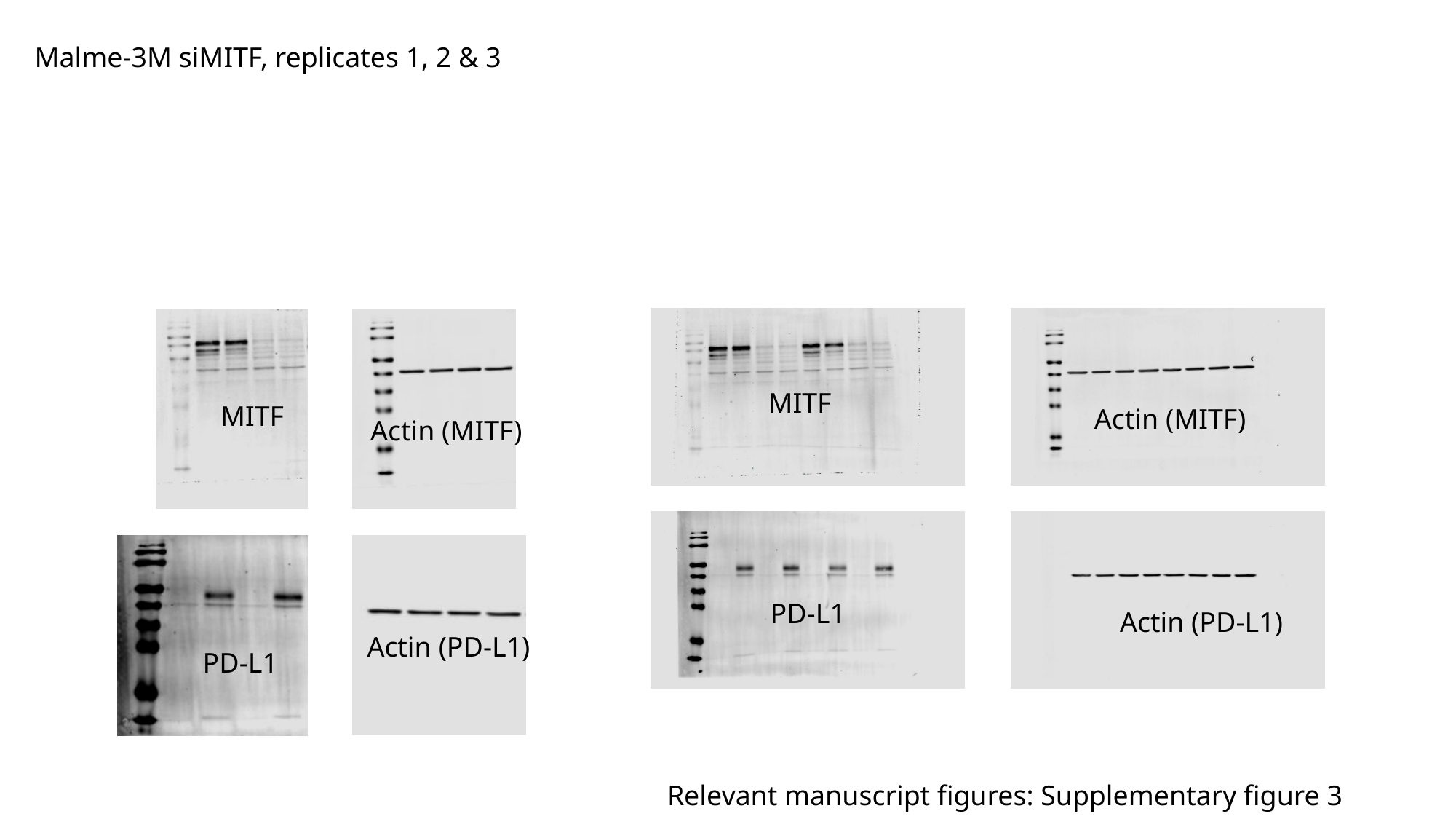

Malme-3M siMITF, replicates 1, 2 & 3
MITF
MITF
Actin (MITF)
Actin (MITF)
PD-L1
Actin (PD-L1)
Actin (PD-L1)
PD-L1
Relevant manuscript figures: Supplementary figure 3

## Slide 16
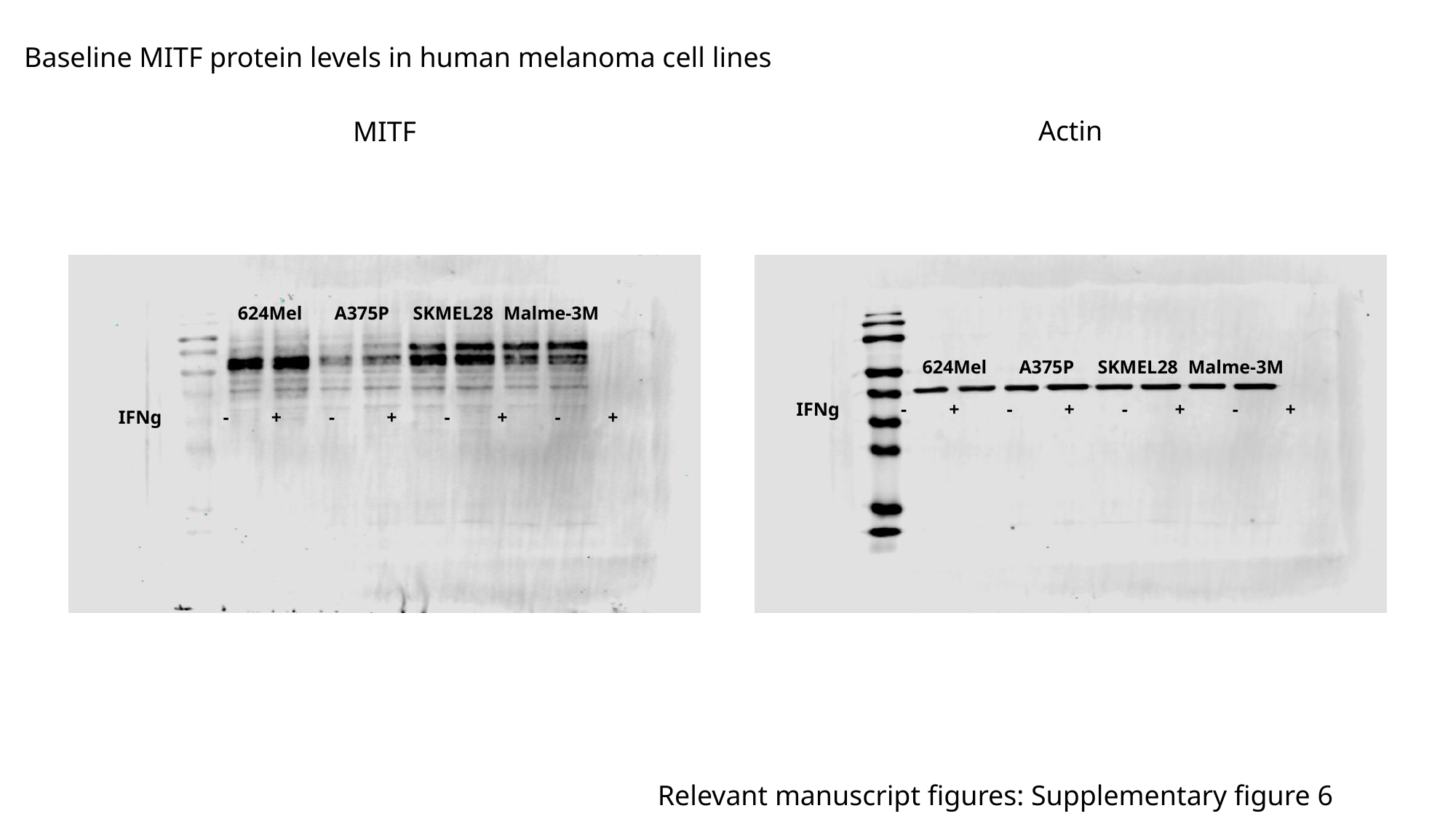

Baseline MITF protein levels in human melanoma cell lines
Actin
MITF
Malme-3M
A375P
SKMEL28
624Mel
Malme-3M
A375P
SKMEL28
624Mel
IFNg - + - + - + - +
IFNg - + - + - + - +
Relevant manuscript figures: Supplementary figure 6
